# Ten Americas: a systematic analysis of life expectancy disparities in the USA

**DOI:** 10.1016/S0140-6736(24)01495-8

**Published:** 2024-12-07

**Authors:** Laura Dwyer-Lindgren, Mathew M Baumann, Zhuochen Li, Yekaterina O Kelly, Chris Schmidt, Chloe Searchinger, Wichada La Motte-Kerr, Thomas J Bollyky, Ali H Mokdad, Christopher JL Murray

**Affiliations:** a Institute for Health Metrics and Evaluation, University of Washington, Seattle, WA, USA; b Department of Health Metrics Sciences, University of Washington, Seattle, WA, USA; c Council on Foreign Relations, Washington, DC, USA

## Abstract

**Background:**

Nearly two decades ago, the Eight Americas study offered a novel lens for examining health inequities in the USA by partitioning the US population into eight groups based on geography, race, urbanicity, income per capita, and homicide rate. That study found gaps of 12·8 years for females and 15·4 years for males in life expectancy in 2001 across these eight groups. In this study, we aimed to update and expand the original Eight Americas study, examining trends in life expectancy from 2000 to 2021 for ten Americas (analogues to the original eight, plus two additional groups comprising the US Latino population), by year, sex, and age group.

**Methods:**

In this systematic analysis, we defined ten mutually exclusive and collectively exhaustive Americas comprising the entire US population, starting with all combinations of county and race and ethnicity, and assigning each to one of the ten Americas based on race and ethnicity and a variable combination of geographical location, metropolitan status, income, and Black–White residential segregation. We adjusted deaths from the National Vital Statistics System to account for misreporting of race and ethnicity on death certificates. We then tabulated deaths from the National Vital Statistics System and population estimates from the US Census Bureau and the National Center for Health Statistics from Jan 1, 2000, to Dec 31, 2021, by America, year, sex, and age, and calculated age-specific mortality rates in each of these strata. Finally, we constructed abridged life tables for each America, year, and sex, and extracted life expectancy at birth, partial life expectancy within five age groups (0–4, 5–24, 25–44, 45–64, and 65–84 years), and remaining life expectancy at age 85 years.

**Findings:**

We defined the ten Americas as: America 1—Asian individuals; America 2—Latino individuals in other counties; America 3—White (majority), Asian, and American Indian or Alaska Native (AIAN) individuals in other counties; America 4—White individuals in non-metropolitan and low-income Northlands; America 5—Latino individuals in the Southwest; America 6—Black individuals in other counties; America 7—Black individuals in highly segregated metropolitan areas; America 8—White individuals in low-income Appalachia and Lower Mississippi Valley; America 9—Black individuals in the non-metropolitan and low-income South; and America 10—AIAN individuals in the West. Large disparities in life expectancy between the Americas were apparent throughout the study period but grew more substantial over time, particularly during the first 2 years of the COVID-19 pandemic. In 2000, life expectancy ranged 12·6 years (95% uncertainty interval 12·2–13·1), from 70·5 years (70·3–70·7) for America 9 to 83·1 years (82·7–83·5) for America 1. The gap between Americas with the lowest and highest life expectancies increased to 13·9 years (12·6–15·2) in 2010, 15·8 years (14·4–17·1) in 2019, 18·9 years (17·7–20·2) in 2020, and 20·4 years (19·0–21·8) in 2021. The trends over time in life expectancy varied by America, leading to changes in the ordering of the Americas over this time period. America 10 was the only America to experience substantial declines in life expectancy from 2000 to 2019, and experienced the largest declines from 2019 to 2021. The three Black Americas (Americas 6, 7, and 9) all experienced relatively large increases in life expectancy before 2020, and thus all three had higher life expectancy than America 10 by 2006, despite starting at a lower level in 2000. By 2010, the increase in America 6 was sufficient to also overtake America 8, which had a relatively flat trend from 2000 to 2019. America 5 had relatively similar life expectancy to Americas 3 and 4 in 2000, but a faster rate of increase in life expectancy from 2000 to 2019, and thus higher life expectancy in 2019; however, America 5 experienced a much larger decline in 2020, reversing this advantage. In some cases, these trends varied substantially by sex and age group. There were also large differences in income and educational attainment among the ten Americas, but the patterns in these variables differed from each other and from the patterns in life expectancy in some notable ways. For example, America 3 had the highest income in most years, and the highest proportion of high-school graduates in all years, but was ranked fourth or fifth in life expectancy before 2020.

**Interpretation:**

Our analysis confirms the continued existence of different Americas within the USA. One's life expectancy varies dramatically depending on where one lives, the economic conditions in that location, and one's racial and ethnic identity. This gulf was large at the beginning of the century, only grew larger over the first two decades, and was dramatically exacerbated by the COVID-19 pandemic. These results underscore the vital need to reduce the massive inequity in longevity in the USA, as well as the benefits of detailed analyses of the interacting drivers of health disparities to fully understand the nature of the problem. Such analyses make targeted action possible—local planning and national prioritisation and resource allocation—to address the root causes of poor health for those most disadvantaged so that all Americans can live long, healthy lives, regardless of where they live and their race, ethnicity, or income.

**Funding:**

State of Washington, Bloomberg Philanthropies, Bill & Melinda Gates Foundation.

## Introduction

Nearly two decades ago, a team of researchers published the Eight Americas study, an examination of inequities in US life expectancy.^1^ Although the racial, ethnic, and income disparities in US longevity have long been documented,^2^, ^3^, ^4^, ^5^ the Eight Americas study broke new ground with its insight that US health inequities were best understood as the product of a combination of inter-related contextual factors. The study partitioned the US population into eight groups based on race, geographical region, urbanicity, income per capita, and homicide rates, in the hope of identifying more effective interventions. In 2001, the gap in life expectancy between Americas was 12·8 years for females and 15·4 years for males. The mortality disparities between the Americas were shown to be especially large for young (age 15–44 years) and middle-aged (age 45–59 years) adults, particularly for males. These differences were so stark, the study coauthors concluded, it was as if the highlighted groups resided in separate Americas instead of one.

**Research in context**

**Evidence before this study**

In 2006, the Eight Americas study examined inequities in US health and life expectancy using a novel framing that simultaneously considered a constellation of interrelated contextual factors, ranging from place to race and ethnicity, among others. This study partitioned the US population into eight groups based on race, geographical region, urbanicity, income per capita, and homicide rate, and in doing so, found gaps in life expectancy in 2001 across these eight Americas of more than a decade (12·8 years for females and 15·4 years for males). Other research both before and since this landmark study has consistently found inequalities in life expectancy in the USA, including by geographical location, urbanicity, race and ethnicity, income, educational attainment, and other dimensions. Although some other studies have considered two of these dimensions simultaneously (eg, how life expectancy varies by geographical location and by race and ethnicity)—and typically find further inequalities and complex interactions—studies examining the intersection of more than two of these dimensions remain rare, due to small numbers and other data challenges. We searched PubMed from database inception to Aug 23, 2024, using the search string (“United States”) AND (“life expectancy”) AND (“inequality” OR “disparity”) AND (“geography” OR “region” OR “state” OR “county”) AND (“race” OR “ethnicity”) AND (“urban” OR “rural”) AND (“socioeconomic” OR “income” OR “education”) for studies examining disparities in life expectancy in the USA by geographic location, race and ethnicity, urban and rural status, and socioeconomic status. We identified only two studies aside from the Eight Americas study that stratified life expectancy by at least three of these dimensions simultaneously (in both cases by race, a measure of rural–urban status, and a measure of socioeconomic status). Moreover, although detailed analysis of interacting dimensions of health disparity is crucial for fully understanding the nature of the problem and, in the case of detailed geographical studies, is also useful for local planning and decision making, the sheer volume of results can make it difficult to interpret and act upon them at a national scale.

**Added value of this study**

This study updates and expands the original Eight Americas study, providing insights into the size and evolution of US inequalities in longevity in the 21st century, including the first 2 years of the COVID-19 pandemic. Whereas the original Eight Americas study was only able to consider race and not Latino ethnicity, this update adds two new Americas that comprise the US Latino population, bringing the total up to ten. We also refine the methodological approach to incorporate new data sources and more nuanced adjustment for misclassification of race and ethnicity on death certificates, and to assess uncertainty for all estimated quantities.

**Implications of all the available evidence**

Disparities in life expectancy between the Americas were already large at the turn of the century, but grew even larger during the 2000s and 2010s and larger still during the first 2 years of the COVID-19 pandemic. The trajectory of life expectancy—both before and during the pandemic—varied among Americas, leading to several changes in ordering of the Americas between 2000 and 2021. Differences in income and educational attainment are likely to explain some of the differences observed in life expectancy but are clearly not the sole driving force. In addition to the disturbing sizes of the disparities, several other alarming trends have emerged. From 2000 to 2019, White American individuals in low-income counties in Appalachia and the Lower Mississippi Valley (America 8) experienced no increase in life expectancy, whereas American Indian or Alaska Native individuals in the West (America 10) experienced a substantial decline. The other eight Americas experienced increases in life expectancy from 2000 to 2010 (notably in the three Black Americas [6, 7, and 9], which had the lowest life expectancy in 2000); however, there were minimal further improvements from 2010 to 2019. Life expectancy declined in all ten Americas in 2020, and rebounded partly in only three Americas in 2021, with the Americas that were worst off before the pandemic taking some of the largest overall losses from 2019 to 2021. We must take collective action to invest in equitable health care, education, and employment opportunities, and to challenge the systemic barriers that create and perpetuate these inequities.

20 years later, US life expectancy has shifted in the wrong direction, falling further behind that of most other peer wealthy nations.^6^, ^7^, ^8^, ^9^ There is evidence that the growing geographical divides in health and longevity within the USA—among regions,^10^ states,^11^, ^12^, ^13^ and counties^3^, ^4^, ^14^—have been one driver in the overall stalling of progress in US life expectancy in recent years.^15^ The role of socioeconomic disparities is also well documented, with substantial gradients in US life expectancy by income^4^ and education,^16^, ^17^ as is the role of systemic racism, which drives large racial and ethnic disparities in life expectancy.^18^, ^19^ The dramatically unequal impact of the COVID-19 pandemic in the USA^20^ has dimmed the one bright spot in US life expectancy—that racial and ethnic disparities had appeared to be shrinking in the first decade of the 21st century.^5^

In this study, we examine disparities in US longevity by updating and expanding the original Eight Americas study, estimating trends in life expectancy from 2000 to 2021 for ten Americas (including analogues to the original eight groups, plus two additional groups comprising the US Hispanic or Latino [hereafter Latino] population), by year, sex, and age group. This grouping of the US population based on county and race and ethnicity is by no means the only division that could be used for understanding the large inequalities in US life expectancy. Disparities observed along any one dimension (eg, race and ethnicity, socioeconomic status, or geographical location) are typically large, yet examining how life expectancy varies along multiple dimensions simultaneously reveals the even larger health disparities that exist in American society. For example, in 2019, life expectancy varied by 12·6 years among five racial and ethnic groups nationally; by 27·2 years among counties; and by a massive 36·3 years when considering county and racial and ethnic group simultaneously.^3^ Similarly, a study focusing on life expectancy at age 40 years from 2001 to 2014 found a gap of 7 years between the bottom and top income quartiles nationally, which widened to 10·6 years when comparing the bottom and top income quartiles across the 100 most populous counties.^4^

Although these types of analyses examining detailed geographical patterns simultaneously with other dimensions of inequality are crucial for better understanding the nuanced interplay between place-based and other types of inequities, they are difficult to expand beyond two dimensions, as the size of each stratum becomes increasingly small, posing difficulties for reliably estimating life expectancy. Moreover, although this level of detail is useful and necessary for local planning purposes, at an aggregate level, having results for hundreds or thousands of strata can be difficult to interpret and act upon. Revisiting the Eight Americas study framework now provides important updated insights into the inter-related drivers of mortality disparities, including during the COVID-19 pandemic.

## Methods

### Definition of the ten Americas

We defined ten mutually exclusive and collectively exhaustive Americas comprising the entire US population, starting with all combinations of county and race and ethnicity, and assigning each county and race and ethnicity combination to one of the ten Americas based on race and ethnicity and a variable combination of geographical location, metropolitan status, income, and Black–White residential segregation.

The definitions of the ten Americas are presented in table 1 and visualised in the appendix (pp 4–5). We describe them here alphabetically by race and ethnicity, but for convenience, the Americas are numbered based on their life expectancy in 2021. Specific regions within the definitions are defined below. America 10 was comprised of non-Latino American Indian or Alaska Native (AIAN) individuals living in the West (not including the Pacific Coast). America 1 comprised the combined non-Latino Asian and Native Hawaiian or Pacific Islander (NHPI) population living in counties where the NHPI population was less than 30% of the total Asian and NHPI population in 2020. It was not possible to fully distinguish Asian and NHPI individuals in the deaths data before 2011, and so this approach was used to ensure that America 1 predominantly represents the Asian population, which has distinctly different health outcomes compared with the NHPI population. The non-Latino Black population was included in Americas 6, 7, and 9. America 7 comprised Black individuals living in highly segregated, high-population metropolitan centres. America 9 comprised Black individuals living in non-metropolitan, low-income counties in the Lower Mississippi Valley or the Deep South. America 6 comprised Black populations that were not a part of Americas 7 or 9. The Latino population (of all races) was included in Americas 2 and 5. Latino populations living in the Southwest were included in America 5, whereas Latino populations in the rest of the USA were included in America 2. Americas 4 and 8 both comprised non-Latino White populations living in low-income counties. America 4 was limited to non-metropolitan counties in the Northlands, whereas America 8 was limited to counties in Appalachia and the Lower Mississippi Valley. America 3 comprised Asian and NHPI populations that were not a part of America 1, White populations that were not a part of America 4 or 8, and AIAN populations that were not a part of America 10.

**Table 1:** Definitions of the ten Americas

	**Definition**	Number of counties*	**Population in 2021 (millions)**	**Proportion of total national population, 2021 (n=332·64 million)**	**Proportion of racial and ethnic group population, 2021**
America 1: Asian	The combined Asian and NHPI population living in counties where the NHPI population was <30% of the total Asian and NHPI population in 2020	2803 (Asian)	20·67	6·21%	95·06% (Asian, n=21·75 million)
America 2: Latino | Other counties	The Latino population not included in America 5	2777 (Latino)	46·17	13·88%	73·70% (Latino, n=62·65 million)
America 3: White (majority), Asian, AIAN | Other counties	The combined Asian and NHPI population not included in America 1, the White population not included in Americas 4 and 8, and the AIAN population not included in America 10	2381 (AIAN); 340 (Asian and NHPI); 2662 (White)	1·47 (AIAN); 1·07 (Asian and NHPI); 190·66 (White)	0·44% (AIAN); 0·32% (Asian and NHPI); 57·32% (White)	52·60% (AIAN, n=2·79 million); 4·94% (Asian and NHPI, n=21·75 million); 94·72% (White), n=201·29 million
America 4: White | Non-metropolitan and low-income Northlands	The White population living in non-metropolitan counties in Iowa, Minnesota, Montana, Nebraska, North Dakota, and South Dakota where the income per capita among the White population was less than $32 363† in 2020	47 (White)	0·29	0·09%	0·14% (White, n=201·29 million)
America 5: Latino | Southwest	The Latino population living in counties in Arizona, Colorado, New Mexico, and Texas	366 (Latino)	16·47	4·95%	26·30% (Latino, n=62·65 million)
America 6: Black | Other counties	The Black population not included in Americas 7 and 9	2700 (Black)	31·78	9·55%	71·95% (Black, n=44·17 million)
America 7: Black | Highly segregated metropolitan areas	The Black population living in highly segregated, high-population metropolitan counties	88 (Black)	10·26	3·08%	23·23% (Black, n=44·17 million)
America 8: White | Low-income Appalachia and Lower Mississippi Valley	The White population living in counties in Appalachia and the Lower Mississippi Valley where the income per capita among the White population was less than $32 363† in 2020	434 (White)	10·34	3·11%	5·14% (White, n=201·29 million)
America 9: Black | Non-metropolitan and low-income South	The Black population living in non-metropolitan counties in the Lower Mississippi Valley or the Deep South where the income per capita among the Black population was less than $32 363† in 2020	355 (Black)	2·13	0·64%	4·82% (Black, n=44·17 million)
America 10: AIAN | West	The AIAN population living in counties in Arizona, Colorado, Idaho, Kansas, Minnesota, Montana, Nebraska, Nevada, New Mexico, North Dakota, Oklahoma, South Dakota, Utah, and Wyoming	762 (AIAN)	1·32	0·40%	47·40% (AIAN, n=2·79 million)

AIAN=American Indian or Alaska Native. NHPI=Native Hawaiian or Pacific Islander.

* Indicates the number of counties where residents of the specified racial and ethnic group are included in a given America. Some county boundaries changed during the study period. To create historically stable geographical units for analysis, a small number of counties were combined. This reduced the number of units analysed from 3143 counties to 3110 counties or groups of counties.

† US$32 363 was the median all-race income per capita among all counties.

For the purpose of applying these definitions, a county was considered high-population metropolitan if the county had a US Department of Agriculture 2023 Rural–Urban Continuum Code^21^ of 1 (counties in metropolitan areas of 1 million population or more); non-metropolitan if the county had a non-metropolitan Rural–Urban Continuum Code (codes 4–9); highly segregated if the county had a Black–White dissimilarity index of 60 or higher for 2018–2022;^22^, ^23^ and low-income if the county's income per capita in 2020 for the specific racial and ethnic group considered was below the median county income per capita for all racial and ethnic groups combined (ie, US$32 363). The West (not including the Pacific Coast, as in the Eight Americas study)^1^ was defined as counties in Arizona, Colorado, Idaho, Kansas, Minnesota, Montana, Nebraska, Nevada, New Mexico, North Dakota, Oklahoma, South Dakota, Utah, and Wyoming. Northlands was defined as counties in Iowa, Minnesota, North Dakota, South Dakota, Nebraska, or Montana, as in the Eight Americas study.^1^ The Lower Mississippi Valley was defined as counties designated by the National Park Service as part of the Lower Mississippi Delta Region.^24^ The Deep South was defined as counties in South Carolina, Georgia, Alabama, Mississippi, or Louisiana. Appalachia was defined as counties served by the Appalachian Regional Commission.^25^ Finally, the Southwest was defined as counties in Arizona, Colorado, New Mexico, and Texas; previous research has identified this region as having relatively low life expectancy among the Latino population (median county life expectancy 79·3 years [IQR 77·5–81·8] in this region *vs* 84·8 years [82·6–86·8] elsewhere).^3^

These definitions differed from the original Eight Americas definitions in several key ways. First, the original Eight Americas study did not consider Latino ethnicity, so Latino Americans were grouped into one of the Americas based on their race; most Latino Americans self-report as White (92·7% in 2000),^26^ so this population was likely to have been predominantly included in America 3 in the original study. Second, we used updated data for 2020 to identify low-income counties for the purpose of defining the ten Americas used in this study, whereas the original study based the America definitions on data from 1990. Third, we replaced urban (based on population size) with high-population metropolitan and rural (based on population density) with non-metropolitan, based on 2023 US Department of Agriculture Rural–Urban Continuum Codes. Fourth, for defining America 7, we replaced high-risk, as identified by high homicide rate, with highly segregated, based on a large (and growing) literature on the role of racial segregation as a determinant of health outcomes.^27^, ^28^, ^29^ Fifth, we refined the definitions of regional areas (eg, Appalachia and the Lower Mississippi Valley) for improved clarity and ease of explanation. Sixth, we used a single threshold for defining low-income counties, whereas the original study used race-specific thresholds.

### Data sources and analysis

Data sources for variables used in defining the ten Americas are described in the appendix (pp 6–8).

Deaths data were from the Multiple Cause of Death Files from the National Vital Statistics System.^30^ These files contain individual-level records for all deaths occurring within the USA and have detail on the decedent's date of death, county of residence, age at death, sex, race, and ethnicity. In 2003, the National Center for Health Statistics of the US Centers for Disease Control and Prevention released a revised standard death certificate that included updates to the race variable to allow for multiple races to be selected, in accordance with revised standards set forth in 1997 by the Office of Management and Budget.^31^ This revision was adopted at different times by different states and was not fully implemented across all states until partway through 2017.^32^ Consequently, the data for our study period include a mix of records where only a single race could be reported and those where multiple races could be reported. For consistency throughout the study period, we used the bridged (ie, single) race imputed by the National Center for Health Statistics for individuals with multiple races reported.^33^ The National Center for Health Statistics discontinued race bridging after 2020, so for 2021, we implemented the same bridging methodology. We tabulated deaths by year, county, age, sex, and race and ethnicity.

Race and ethnicity are recorded on death certificates by funeral directors, ideally based on information from an informant such as a family member; however, race and ethnicity are sometimes misclassified relative to how a decedent self-identified, possibly due to funeral directors relying on observation or an informant supplying incorrect information. This misclassification creates an inconsistency between the deaths (numerator) and the population estimates (denominator) for calculating mortality rates and ultimately leads to bias.^34^ To address this issue, we leveraged previously published misclassification ratios,^34^ which were calculated using linked survey (ie, self-reported) and death certificate data. Separate misclassification ratios were available for each race and ethnicity; the numerator was the number of deaths in a given race and ethnicity group when tabulated based on self-report, and the denominator was the number of deaths in a given race and ethnicity group when tabulated based on the race and ethnicity recorded on death certificates. The ratios were defined in this way so that they could be multiplied by mortality rates where deaths are tabulated based on the race and ethnicity recorded on the death certificate, and provide a population-level (not individual-level) correction for the net effect of misclassification. Misclassification ratios were available by age and sex, census region (n=4), and co-ethnic density, a measure of the concentration of the Latino or AIAN population within a given county. To represent uncertainty in these ratios, we generated 1000 simulations from a log-normal distribution with mean matching the reported misclassification ratio and SD matching the associated SE. We combined simulated misclassification ratios across different dimensions (age and sex, region, and co-ethnic density) using previously described methods,^3^ and then multiplied the reported number of deaths in each year, county, age, sex, and race and ethnicity by the corresponding 1000 simulations of the misclassification ratio. Finally, we scaled the resulting corrected number of deaths within each year, county, age, sex, and simulation to ensure that this adjustment did not change the overall number of deaths recorded.

We merged the tabulated and adjusted deaths with population estimates by year, county, age, sex, and race and ethnicity. For 2000–20, we used bridged race population estimates from the National Center for Health Statistics.^26^, ^35^ For 2020–21, we additionally used population estimates from the US Census Bureau.^36^ The latter used a different categorisation of race and ethnicity that includes a separate Two or More Races group. To align with the race and ethnicity categorisation used in this analysis, we split this population into bridged races, which were added to the totals for each single race group (appendix p 9). Intercensal population estimates have not yet been released for 2010–20, and the postcensal estimates for 2010–20 based on the 2010 census are, in many cases, discordant with the postcensal estimates for 2020–21 based on the 2020 census. To address this discontinuity, we applied an adjustment to the 2010–20 population estimates based on the methodology the US Census Bureau applies to create intercensal population estimates (appendix pp 9–11).^37^

After combining the simulated deaths and population data, we assigned each county and race and ethnicity to one of the ten Americas using the definitions described above and summed up deaths and population by year, America, age, sex, and simulation. To represent the stochastic variation in the number of deaths observed in a given stratum, we simulated from a Poisson distribution with rate equal to the simulated number of deaths. We then used these simulated deaths to calculate mortality rates for each year, America, age, sex, and simulation. From the age-specific mortality rates, we constructed abridged life tables for each year, America, sex, and simulation using standard life table techniques, and the Horiuchi and Coale method for estimating life expectancy in the terminal age group (appendix pp 12–13).^38^, ^39^ We calculated point estimates from the mean and 95% uncertainty intervals from the 2·5th to the 97·5th percentile of the 1000 simulations. These uncertainty intervals reflect uncertainty in the estimates for each America due to the misclassification correction and stochastic variation; they tend to be larger when the misclassification correction is larger and when the population is smaller. We focus primarily on life expectancy at birth but also report partial life expectancy for five age ranges (0–4, 5–24, 25–44, 45–64, and 65–84 years) and life expectancy at age 85 years in order to examine differences by age. Partial life expectancy (also known as temporary life expectancy) is the mean number of years lived within each age range, and has a maximum value equal to the length of the age range (ie, if everyone survived from birth to age 5 years, the partial life expectancy in age group 0–4 years would be 5 years).^40^

For additional context, we also considered trends in each of the ten Americas for three socioeconomic variables: income per capita, the proportion of the population aged 25 years and older that had graduated from high school, and the proportion of the population aged 25 years and older that had graduated from college. We used county-level estimates of these three variables based on data from the 2000 decennial census and the America Community Survey (ACS) 5-year tabulated data files and smoothed using a small area estimation model (appendix pp 14–19). The most recent available data for these variables are in the 2018–22 ACS 5-year file, which we used to inform estimates for 2020 (the mid-year). We therefore examined these variables for a slightly shorter timeframe, 2000–20, compared with the life expectancy analysis.

This study complies with the Guidelines for Accurate and Transparent Health Estimates Reporting (appendix p 3). This research received institutional review board approval from the University of Washington (Seattle, WA, USA). No primary data were collected for this study and we had no contact with human subjects.

### Role of the funding source

The funders had no role in study design, data collection, data analysis, or writing of the report.

## Results

**Figure 1:**
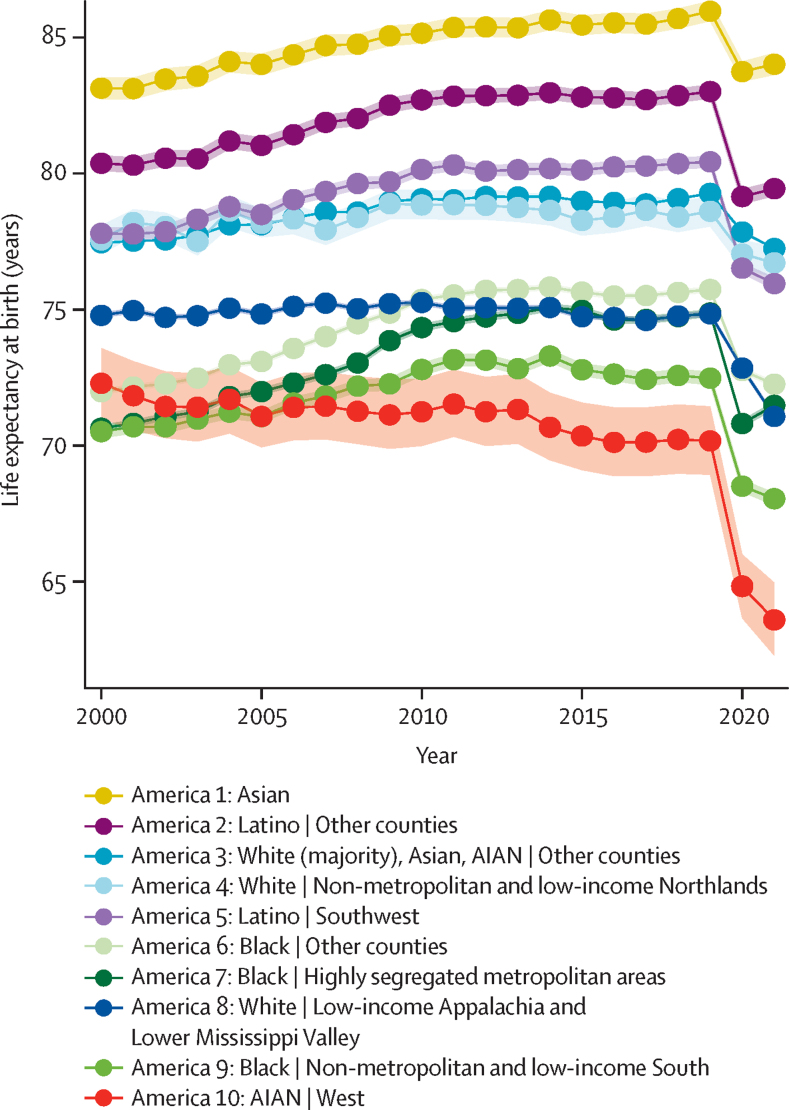
Life expectancy at birth in the ten Americas, 2000–21 Shaded areas indicate 95% uncertainty intervals. AIAN=American Indian or Alaska Native.

In 2000, life expectancy was lowest for Black Americans in non-metropolitan and low-income counties in the South (America 9, 70·5 years [95% uncertainty interval 70·3–70·7]), Black Americans in highly segregated metropolitan areas (America 7, 70·6 years [70·5–70·8]), Black Americans in other counties (America 6, 72·0 years [71·9–72·1]), and AIAN individuals in the West (America 10, 72·3 years [71·0–73·6]; figure 1). Life expectancy among White Americans in low-income counties in Appalachia and the Lower Mississippi Valley (America 8) was the next lowest (74·8 years [74·7–74·9]) but was notably higher than among Black and AIAN individuals (2·5 years [1·2–3·8] higher than America 10). White Americans in non-metropolitan, low-income counties in the Northlands (America 4) had similar life expectancy to America 3, which is mostly White Americans in other counties (77·6 years [77·0–78·1] *vs* 77·5 years [77·4–77·5]), and both had notably higher life expectancy than White Americans in America 8 (by 2·7 years [2·6–2·8] for America 3). Latino Americans in the Southwest (America 5) had slightly higher life expectancy than Americas 3 and 4 (77·8 years [77·6–78·0]), whereas Latino Americans elsewhere (America 2) had substantially higher life expectancy (80·4 years [80·1–80·7], 2·6 years [2·4–2·8] higher than America 5). Asian Americans (America 1) had the highest life expectancy (83·1 years [82·7–83·5]), 2·8 years (2·2–3·3) higher than the next highest group, Latino Americans in other counties (America 2), and fully 12·6 years (12·2–13·1) higher than the group with the lowest life expectancy, Black Americans in non-metropolitan and low-income counties in the South (America 9; table 2).

**Table 2:** Life expectancy at birth in the ten Americas

	**Life expectancy, 2000**	**Change, 2000–10**	**Life expectancy, 2010**	**Change, 2010–19**	**Life expectancy, 2019**	**Change, 2019–20**	**Life expectancy, 2020**	**Change, 2020–21**	**Life expectancy, 2021**	**Change, 2000–19**	**Change, 2019–21**
America 1: Asian	83·1 (82·7–83·5)	2·0 (1·8 to 2·2)*	85·2 (84·8–85·5)	0·8 (0·7 to 1·0)*	86·0 (85·6–86·3)	−2·2 (−2·3 to −2·1)*	83·7 (83·4–84·1)	0·3 (0·2 to 0·4)*	84·0 (83·6–84·4)	2·8 (2·6 to 3·0)*	−1·9 (−2·1 to −1·8)*
America 2: Latino | Other counties	80·4 (80·1–80·7)	2·3 (2·2 to 2·5)*	82·7 (82·4–83·0)	0·3 (0·2 to 0·4)*	83·0 (82·8–83·3)	−3·9 (−4·0 to −3·8)*	79·2 (78·9–79·4)	0·3 (0·2 to 0·4)*	79·4 (79·2–79·7)	2·6 (2·5 to 2·8)*	−3·6 (−3·7 to −3·5)*
America 3: White (majority), Asian, AIAN | Other counties	77·5 (77·4–77·5)	1·6 (1·6 to 1·6)*	79·1 (79·0–79·1)	0·2 (0·2 to 0·2)*	79·3 (79·2–79·3)	−1·4 (−1·5 to −1·4)*	77·9 (77·8–77·9)	−0·6 (−0·6 to −0·6)*	77·2 (77·2–77·3)	1·8 (1·8 to 1·8)*	−2·0 (−2·1 to −2·0)*
America 4: White | Non-metropolitan and low-income Northlands	77·6 (77·0–78·1)	1·3 (0·6 to 2·0)*	78·8 (78·3–79·4)	−0·2 (−1·0 to 0·5)	78·6 (78·1–79·2)	−1·6 (−2·3 to −0·8)*	77·0 (76·5–77·6)	−0·3 (−1·0 to 0·4)	76·7 (76·2–77·3)	1·0 (0·3 to 1·8)*	−1·9 (−2·7 to −1·1)*
America 5: Latino | Southwest	77·8 (77·6–78·0)	2·4 (2·2 to 2·5)*	80·1 (79·9–80·4)	0·3 (0·1 to 0·4)*	80·4 (80·2–80·6)	−3·9 (−4·0 to −3·8)*	76·5 (76·3–76·7)	−0·6 (−0·7 to −0·4)*	76·0 (75·7–76·2)	2·6 (2·5 to 2·8)*	−4·5 (−4·6 to −4·3)*
America 6: Black | Other counties	72·0 (71·9–72·1)	3·4 (3·3 to 3·5)*	75·4 (75·3–75·5)	0·4 (0·3 to 0·5)*	75·7 (75·6–75·8)	−3·0 (−3·1 to −2·9)*	72·8 (72·6–72·9)	−0·5 (−0·6 to −0·4)*	72·3 (72·1–72·4)	3·8 (3·7 to 3·9)*	−3·5 (−3·6 to −3·4)*
America 7: Black | Highly segregated metropolitan areas	70·6 (70·5–70·8)	3·7 (3·5 to 3·9)*	74·3 (74·2–74·5)	0·5 (0·4 to 0·7)*	74·9 (74·7–75·0)	−4·1 (−4·2 to −3·9)*	70·8 (70·7–70·9)	0·7 (0·5 to 0·8)*	71·5 (71·3–71·6)	4·2 (4·1 to 4·4)*	−3·4 (−3·5 to −3·2)*
America 8: White | Low-income Appalachia and Lower Mississippi Valley	74·8 (74·7–74·9)	0·5 (0·3 to 0·6)*	75·3 (75·2–75·4)	−0·4 (−0·5 to −0·3)*	74·8 (74·7–74·9)	−2·0 (−2·1 to −1·9)*	72·8 (72·7–72·9)	−1·8 (−1·9 to −1·6)*	71·1 (71·0–71·2)	0·1 (−0·1 to 0·2)	−3·8 (−3·9 to −3·6)*
America 9: Black | Non-metropolitan and low-income South	70·5 (70·3–70·7)	2·3 (2·0 to 2·6)*	72·8 (72·6–73·0)	−0·3 (−0·6 to 0·0)*	72·5 (72·2–72·7)	−4·0 (−4·3 to −3·6)*	68·5 (68·3–68·7)	−0·5 (−0·8 to −0·1)*	68·0 (67·8–68·3)	2·0 (1·6 to 2·3)*	−4·4 (−4·7 to −4·1)*
America 10: AIAN | West	72·3 (71·0–73·6)	−1·0 (−1·5 to −0·6)*	71·2 (70·0–72·4)	−1·1 (−1·5 to −0·6)*	70·2 (68·9–71·4)	−5·3 (−5·8 to −4·9)*	64·8 (63·7–66·0)	−1·2 (−1·7 to −0·8)*	63·6 (62·3–65·0)	−2·1 (−2·6 to −1·6)*	−6·6 (−7·0 to −6·1)*

Data are mean (95% uncertainty interval). AIAN=American Indian or Alaska Native.

* Indicates that the uncertainty bounds do not encompass 0.

Between 2000 and 2010, life expectancy increased for every America except for AIAN individuals in the West (America 10), which experienced a 1·0-year (95% uncertainty interval 0·6–1·5) decline in life expectancy over this period (figure 1). The increases were the largest for two of the three Black Americas (3·7 years [3·5–3·9] for America 7 and 3·4 years [3·3–3·5] for America 6), followed by the two Latino Americas (2·4 years [2·2–2·5] for America 5 and 2·3 years [2·2–2·5] for America 2), the remaining Black America (2·3 years [2·0–2·6] for America 9), Asian America (2·0 years [1·8–2·2] for America 1), and White, Asian, and AIAN individuals in other counties (1·6 years [1·6–1·6] for America 3). The two entirely White Americas had the smallest increases, at 1·3 years (0·6–2·0) for White Americans in non-metropolitan, low-income counties in the Northlands (America 4) and 0·5 years (0·3–0·6) for White Americans in low-income counties in Appalachia and the Lower Mississippi Valley (America 8). These differential trends led to important changes in the ranking among Americas. Americas 1, 2, and 5 retained the top three spots; however, whereas America 5 had only slightly higher life expectancy than Americas 3 and 4 in 2000, this gap had widened by 2010. In the bottom half of the rankings, AIAN individuals in the West (America 10) fell to the lowest spot, being overtaken by the three Black Americas (Americas 6, 7, and 9), and Black Americans in other counties (America 6) caught up with White Americans in low-income counties in Appalachia and the Lower Mississippi Valley (America 8). The gap in life expectancy overall between the lowest (America 10) and highest (America 1) groups increased slightly to 13·9 years (12·6–15·2; table 2).

Between 2010 and 2019, life expectancy declined again for AIAN individuals in the West (America 10, 1·1 years [95% uncertainty interval 0·6 to 1·5]) and also declined, albeit by a smaller amount, for White Americans in low-income counties in Appalachia and the Lower Mississippi Valley (America 8, 0·4 years [0·3 to 0·5]), Black Americans in non-metropolitan and low-income counties in the South (America 9, 0·3 years [0·0 to 0·6]), and White Americans in non-metropolitan and low-income counties in the Northlands (America 4, 0·2 years [−1·0 to 0·5]; figure 1). Life expectancy increased in the other six Americas, but by substantially smaller amounts compared with the previous decade: 0·2 years (0·2–0·2) among White, Asian, and AIAN individuals in other counties (America 3), 0·3 years (0·1–0·4) among Latino individuals in the Southwest (America 5), 0·3 years (0·2–0·4) among Latino individuals elsewhere (America 2), 0·4 years (0·3–0·5) among Black individuals in other counties (America 6), 0·5 years (0·4–0·7) among Black individuals in highly segregated metropolitan areas (America 7), and 0·8 years (0·7–1·0) among Asian individuals (America 1). These relatively small changes meant that the rankings remained effectively the same from 2010 to 2019. However, the fact that the largest gain was in the group with the highest life expectancy, and the largest decline was in the group with the lowest life expectancy, meant that the overall gap in life expectancy from lowest to highest increased substantially to 15·8 years (14·4–17·1; table 2) in 2019.

Life expectancy declined substantially for all Americas during the first year of the COVID-19 pandemic, but the amount of decline varied dramatically, from 1·4 years (95% uncertainty interval 1·4–1·5) for White, Asian, and AIAN individuals in other counties (America 3) to 5·3 years (4·9–5·8) for AIAN individuals in the West (America 10; figure 1). Notably, the very large declines in the two Latino Americas (3·9 years [3·8–4·0] in America 2 and 3·9 years [3·8–4·0] in America 5) caused life expectancy in America 5 to drop below both Americas 3 and 4, which are predominantly White. The larger decline for Black individuals in highly segregated metropolitan areas (America 7, 4·1 years [3·9–4·2]) compared with White individuals in low-income counties in Appalachia and the Lower Mississippi Valley (America 8, 2·0 years [1·9–2·1]) led to America 8 regaining the advantage. The exceedingly large decline in America 10 and the comparatively modest decline among Asian individuals (America 1) led again to an increase in the overall gap in life expectancy to 18·9 years (17·7–20·2; table 2) in 2020.

From 2020 to 2021, life expectancy rebounded slightly for Black Americans in highly segregated metropolitan areas (America 7, 0·7 years [95% uncertainty interval 0·5–0·8]), Asian Americans (America 1, 0·3 years [0·2–0·4]), and Latino Americans in other counties (America 2, 0·3 years [0·2–0·4]), but continued to decline for the other Americas, although in the case of White Americans in non-metropolitan and low-income counties in the Northlands (America 4), the estimated change was highly uncertain and the uncertainty interval encompassed 0 (figure 1). White Americans in low-income Appalachia and the Lower Mississippi Valley (America 8) had the largest decline (1·8 years [1·6–1·9]). As a result of this decline, as well as the increase in life expectancy among Black Americans in highly segregated metropolitan areas (America 7), life expectancy in 2021 was higher in America 7 than America 8, a reversal from the situation in 2020. AIAN individuals in the West (America 10) also had a relatively large decline (1·2 years [0·8–1·7]) in life expectancy. This decline in the group with the lowest life expectancy (America 10), coupled with the increase in the group with the highest life expectancy (America 1), further widened the gap in life expectancy between the lowest and highest groups to 20·4 years (19·0–21·8; table 2).

**Figure 2:**
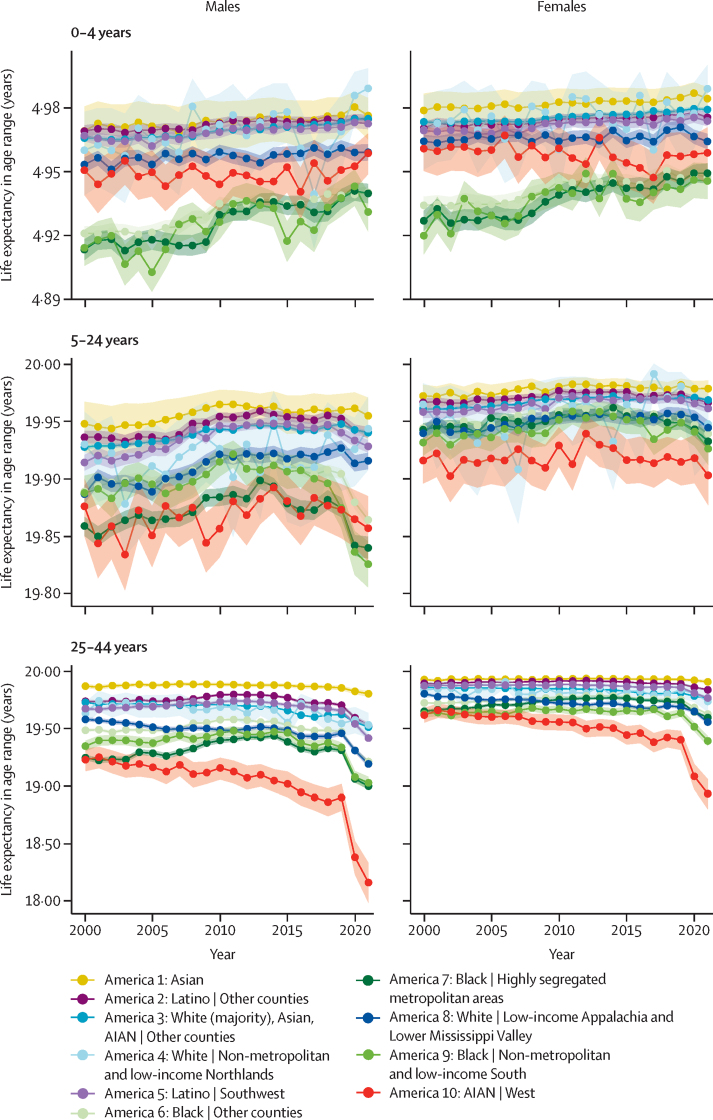
Life expectancy by age and sex in the ten Americas, ages 0–44 years, 2000–21 Shaded areas indicate 95% uncertainty intervals. Life expectancy in age range is the partial life expectancy for age groups 0–4, 5–24, and 25–44 years. AIAN=American Indian or Alaska Native.

**Figure 3:**
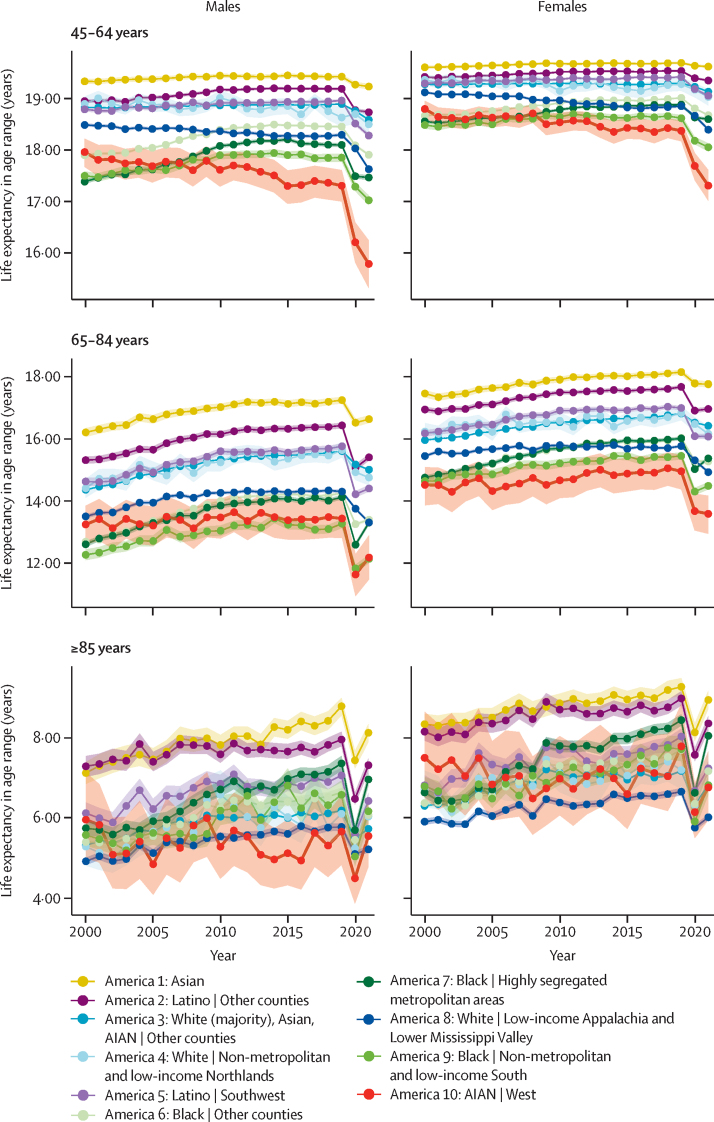
Life expectancy by age and sex in the ten Americas, ages 45 years and older, 2000–21 Shaded areas indicate 95% uncertainty intervals. Life expectancy in age range is the partial life expectancy for age groups 45–64 and 65–84 years and is the remaining life expectancy for age group 85 years and older. AIAN=American Indian or Alaska Native.

In some cases, the disparities among Americas differed in important ways when examined separately by age and sex (Figure 2, Figure 3). Among children under 5 years of age, partial life expectancy was lowest for the three Black Americas (Americas 6, 7, and 9) throughout 2000–21, in contrast to life expectancy overall, which was lowest for AIAN individuals in the West (America 10) from approximately 2006 onwards. Among male children, adolescents, and young adults aged 5–24 years, life expectancy was lowest in nearly half the years among Black individuals in highly segregated metropolitan areas (America 7). Focusing on the pre-COVID pandemic era, relatively little improvement was made in partial life expectancy for males or females in the age 25–44 years range; the same was true in the age 45–64 years range except in the three Black Americas (Americas 6, 7, and 9), which saw some improvement from 2000 to 2010, mimicking the trend in life expectancy at birth. For AIAN individuals in the West (America 10), the partial life expectancy declined notably in these two age groups but increased or held relatively steady in both younger and older ages, making these age groups likely to be the primary contributor to the declines observed in life expectancy at birth. In contrast, partial life expectancy increased substantially in the 65–84 years and 85 years and older age ranges for both males and females (except for AIAN individuals in the West [America 10] ages 85 years and older for males), with Black Americans in other counties (America 6) and Black Americans in highly segregated metropolitan areas (America 7) having the largest improvements in most cases. However, in most cases the majority of the improvement was from 2000 to 2010, with a relatively steady trend after 2010. Differences in the trend from 2019 to 2021 by age and sex were also apparent. Partial life expectancy declined in both 2020 and 2021 for males and females aged 25–44 years and 45–64 years in every America except White individuals in non-metropolitan and low-income counties in the Northlands (America 4, which had very uncertain estimates due to the group's small population size), where there was a slight increase in 2020 for males. In general, the larger declines from 2019 to 2021 in these age groups were in the Americas that had lower partial life expectancy to begin with. A different pattern was observed for ages 85 years and older, where both males and females in all ten Americas experienced an incomplete rebound in life expectancy in 2021. In contrast, different Americas had different patterns for age group 65–84 years: both males and females in the three White or predominantly White Americas (3, 4, and 8), as well as female AIAN individuals in the West (America 10), saw continued declines in partial life expectancy in 2021, whereas males and females in other Americas experienced either minimal change or incomplete rebounds in partial life expectancy in 2021.

**Figure 4:**
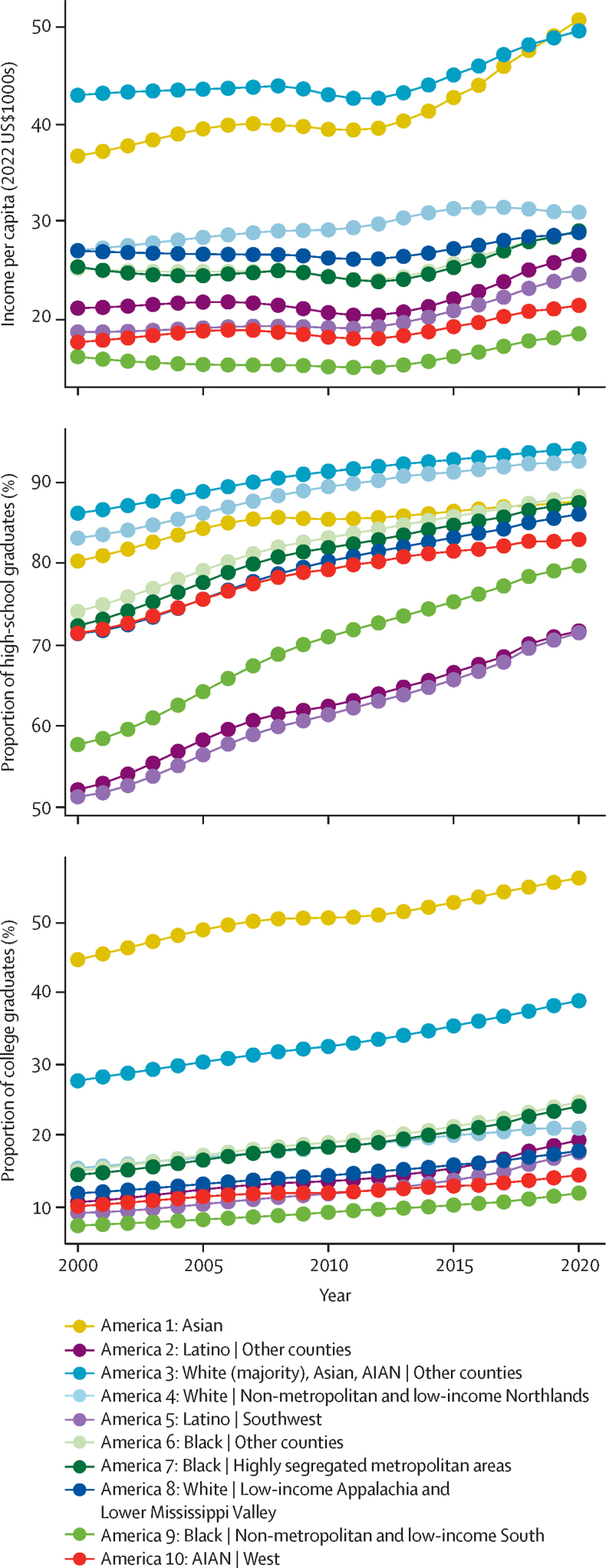
Income per capita and proportions of high-school and college graduates in the ten Americas, 2000–20 AIAN=American Indian or Alaska Native.

Figure 4 shows trends in three socioeconomic variables for each of the ten Americas. In some cases, the differences between Americas were similar for the socioeconomic variables compared with life expectancy. For example, Black individuals in non-metropolitan and low-income counties in the South (America 9) and AIAN individuals in the West (America 10) had the lowest income per capita and proportion of college graduates, and had the lowest life expectancy in most years. At the other extreme, Asian Americans (America 1) were ranked in the top three in all three measures in most years, including having by far the highest proportion of college graduates, and were similarly ranked first in terms of life expectancy. There were also some prominent exceptions. The two Latino Americas (Americas 2 and 5) stand out for having relatively high life expectancy, but among the lowest levels of income and educational attainment, including the lowest proportion of high-school graduates by a substantial margin. America 3, which represents mostly White Americans, is notable for having the highest income in most years, the highest proportion of high-school graduates, and the second highest percentage of college graduates, but only the fourth or fifth highest life expectancy before 2020. The contrast with White individuals in non-metropolitan and low-income counties in the Northlands (America 4) is also notable; compared with America 3, America 4 had considerably fewer college graduates and substantially lower income, but nonetheless had only slightly lower life expectancy.

## Discussion

Revisiting the Eight Americas study two decades later has provided important insights into where progress has (or has not) occurred, and how an inter-related set of contextual factors have contributed to the USA failing so many Americans and falling behind so many of its peer nations.^6^, ^7^, ^8^, ^9^ At the beginning of the 21st century, there was already a 12·6-year gap in life expectancy among Americas, but this gap grew even larger during the 2000s and 2010s and accelerated to 20·4 years after the first 2 years of the COVID-19 pandemic. Other alarming trends were observed in many of the Americas. Before the pandemic, White Americans in low-income counties in Appalachia and the Lower Mississippi Valley (America 8) experienced no increase in life expectancy, whereas AIAN individuals in the West (America 10) experienced a substantial decline. Although the other Americas experienced meaningful increases in life expectancy from 2000 to 2010—with relatively large increases in the three Black Americas (6, 7, and 9), which had the lowest life expectancy in 2000—there was little to no further progress from 2010 to 2019. Life expectancy declined in all ten Americas in 2020, and rebounded partly in only three Americas in 2021, with the Americas that were worst off before the pandemic seeing some of the largest overall losses from 2019 to 2021. These persistent inequities among ten different Americas warrant urgent attention. We hope these estimates inspire action and serve as one tool (among many) for guiding the development of interventions to reduce and ultimately eliminate these inequities by focusing on the health of the most marginalised Americans. By addressing these disparities head-on, we can take an important step towards achieving health equity and improving the wellbeing of all Americans.

We highlight five sets of observations that could help guide future US interventions. First, one way in which our present analysis differs from the original Eight Americas study is in underscoring the health crisis in America 10, the 1·3 million AIAN individuals living in the West. America 10 was the only group in which life expectancy declined over the two-decade period preceding the COVID-19 pandemic. This result confirms studies examining life expectancy and cause-specific mortality by county and racial and ethnic group, which found that AIAN populations experience substantially lower life expectancy as well as higher mortality for almost all leading causes of death and in most counties compared with White, Black, and Latino populations in the USA.^3^, ^14^ Our analysis also confirms the dramatic toll of COVID-19 on the AIAN population.^41^, ^42^ The striking disparities faced by AIAN individuals require immediate action. AIAN individuals have particularly low rates of health insurance coverage in the Southern Plains (23%), Southwest (19%), and Northern Plains (16%).^43^ Low rates of health insurance and chronic underfunding of the Indian Health Service^44^ are barriers to the AIAN population in this America accessing health care. Higher rates of unemployment, lower rates of educational attainment, and historical legacy of systemic discrimination against AIAN people and cultures have contributed to higher rates of excessive alcohol consumption, tobacco use, injuries, and dietary risk factors.^45^, ^46^ It is crucial that we amplify AIAN voices in political and health debates, ensuring that their expertise and insight into what AIAN individuals in America 10 and throughout the USA need guides actions to redress current inequities. Moreover, improvements in education and employment opportunities are essential to alleviating health disparities and fostering socioeconomic growth. Education plays a pivotal role in health disparities, as it directly influences an individual's ability to access and use the resources, care, and opportunities necessary for maintaining good health.^47^ Moreover, education often correlates with income, which can further affect an individual's ability to afford necessary treatments, medications, and nutritious food. Additionally, we must address institutional and interpersonal discrimination against AIAN individuals.^48^ With effort, we can dismantle the systemic barriers—rooted in a history of colonialism, genocide, and forced assimilation^49^—that have perpetuated health inequities for AIAN individuals.

Second, our results highlight that some of the most substantial progress in improving life expectancy in the USA during this study period was in the three Black Americas during the first decade of the century. The gap between life expectancy for Black and White Americans might never have been narrower than it was in the mid-2010s.^5^, ^50^ This is particularly striking when comparing two Americas that are both lower-income and occupy similar geographical locations; the gap between Black individuals in America 9 and White individuals in America 8 was 2·0 years (95% uncertainty interval 1·7–2·2) in 2015, down from 4·3 years (4·0–4·5) in 2000, the result of improving longevity for America 9 and stagnating progress for America 8 over this period. The literature on the relationship between education and health suggests that long-term improvements in the quantity and quality of education available to Black children and young adults over recent decades might be one possible contributor to improved longevity and reduced life expectancy disparities spread broadly over young and middle-aged adults (ages 20–64 years).^51^, ^52^ Reductions in HIV/AIDS mortality and homicide rates over this period—causes of death that, due to structural factors, have disproportionately affected Black communities—are also likely to have contributed.^5^, ^53^, ^54^ In the subsequent years before the COVID-19 pandemic, however, the improvement in life expectancy for the three Black Americas—as in other Americas—largely stalled. An increase in overdose deaths—which initially were far greater among White Americans, but increased rapidly among Black Americans in the 2010s^55^—has contributed, as has an increase in homicide.^56^ Additionally, the slowdown in reductions in cardiovascular disease mortality after decades of improvements^57^—likely to be related to increases in obesity^58^—has probably also contributed to stagnating trends in life expectancy among Black Americans (and likely also among White Americans).^56^ Nonetheless, more population health research is needed to fully understand what drove the initial increase as well as the later stagnation in Americas 6, 7, and 9, as this could inform future efforts to spur further improvements.

Third, by adding two Americas (Americas 2 and 5), this update highlights the unique trends for Latino Americans. Consistent with a large body of previous research comparing life expectancy for Latino and non-Latino White Americans,^59^, ^60^ we found that Latino Americans in other counties (America 2) had substantially longer life expectancy compared with White Americans in Americas 3, 4, and especially 8. Moreover, whereas Latino Americans in the Southwest (America 5) had relatively similar life expectancy to the mostly White population of Americas 3 and 4 in 2000, by 2019, the larger life expectancy increases in America 5 had resulted in a meaningful advantage over Americas 3 and 4. The longer life expectancy for Latino compared with non-Latino White Americans has been largely attributed to the higher life expectancy specifically among foreign-born Latino individuals.^61^, ^62^, ^63^ However, this study also highlights regional differences within the Latino population, specifically that Latino Americans in the Southwest (America 5) have notably lower life expectancy than Latino Americans elsewhere (America 2) and also have lower levels of income and educational attainment, albeit by a relatively small margin. Previous studies have observed higher rates of uninsurance in Southwestern states, especially among the Latino population.^64^ Access to health care, especially preventive and screening, is another determinant of health disparities, with individuals in rural or low-income communities^65^ and those with limited English language skills^66^ facing substantial barriers to obtaining timely, high-quality care. Further research is also needed to fully explain these geographic differences in life expectancy and could help to identify other opportunities for improving health among Latino Americans.

Fourth, our analysis affirms the major role of systemic racism and the resulting racial inequities in the COVID-19 pandemic,^67^, ^68^ with the largest declines in life expectancy during the first year of the pandemic experienced by Americas 2, 5, 6, 7, 9, and 10 (comprising AIAN, Black, and Latino Americans). Other than Latino Americans in other counties (America 2) and Black Americans in highly segregated metropolitan areas (America 7), life expectancy in these same groups continued to fall from 2020 to 2021, particularly among AIAN individuals in the West (America 10). Greater investment in strategies for navigating US racial and ethnic divides during national health emergencies are needed.^69^, ^70^ Previous studies have observed that essential workers in the pandemic were disproportionately AIAN, Black, and Latino; were more likely to live in multigenerational households, where SARS-CoV-2 spreads more easily; and were more likely to face systemic discrimination and socioeconomic disadvantages in accessing health-care services.^71^, ^72^ Individuals deemed essential workers had, on average, lower incomes and education levels and were less likely to be able to work remotely and maintain physical distancing in this pandemic.^73^, ^74^ Many of the worst performing states and territories in a recent study of US interstate differences in COVID-19 outcomes were those with the highest populations of people identifying as Black, Latino, or AIAN.^75^ Moreover, many states were slow to fund outreach to minoritised racial and ethnic communities as part of their initial COVID-19 vaccine rollout plans, despite ample research showing that these communities have historical reasons to mistrust public health campaigns.^76^, ^77^, ^78^

Fifth, this study highlights inequalities among White Americans by geographical location and income level. The contrast between White Americans in non-metropolitan and low-income counties in the Northlands (America 4) and those in other counties (America 3) is interesting because despite the higher income per capita and greater prevalence of college graduates in America 3, life expectancy is very similar between these two groups. This is in stark contrast to White Americans in low-income counties in Appalachia and the Lower Mississippi Valley (America 8), who had substantially lower life expectancy in 2000 and experienced no improvement in life expectancy in the pre-pandemic era. This lack of progress in the pre-pandemic era is especially notable because America 8 had the largest decline in life expectancy during the second year of the COVID-19 pandemic. Consequently, the gap in life expectancy between America 8 and America 4—both White and lower income—grew from 2·8 years (95% uncertainty interval 2·3–3·3) in 2000 to 3·8 years (3·2–4·3) in 2019, and to 5·6 years (5·1–6·2) in 2021. Part of this contrast might be explained by differences in income and educational attainment; although Americas 4 and 8 are both lower income, the income per capita of America 4 is nonetheless somewhat higher and increased more during this period. America 4 also had substantially higher rates of high-school and college graduation.

Previous research has pointed to multiple contributors to the stagnating trends in life expectancy in the USA preceding the COVID-19 pandemic, including rising rates of obesity and skyrocketing death rates due to drug overdoses.^11^, ^58^, ^79^, ^80^ In particular, rising mortality in midlife (ages 25–64 years) has contributed to this trend, and has been attributed in large part to drug overdoses and cardiometabolic diseases;^81^ alcohol abuse and suicide have also been implicated,^82^ but the size of their contribution to stagnating life expectancy is debated.^56^, ^79^ Although access to medical care plays a crucial role in addressing these issues, it alone cannot resolve these population health problems.^83^ Population-level interventions are needed to tackle prominent risks such as poor diet, insufficient physical activity, drug use, excessive alcohol use, and high blood pressure. A comprehensive approach that includes preventive measures, public health initiatives, and societal efforts is required.^84^, ^85^

This study has several limitations. First, there is known to be substantial misreporting of race and ethnicity on death certificates, and although our approach incorporates a correction for this misclassification, it is necessarily somewhat crude and increases the uncertainty associated with our estimates, particularly for America 10. Second, the underlying data from the ACS and the 2000 Census on income per capita and educational attainment are tabulated using different racial and ethnic groups compared with those used in this analysis, such that there is a mismatch for three groups (AIAN, combined Asian and NHPI, and Black); in this analysis, these groups refer to non-Latino individuals with this race as their primary racial identity, whereas in tabulated ACS and Census data, these groups include both Latino and non-Latino individuals with this race as their only racial identity. If income or educational attainment are systematically different between Latino and non-Latino or multiracial versus non-multiracial individuals who identify as AIAN, Asian, NHPI, or Black, this mismatch could lead to bias in the estimates of income and educational attainment for these groups and the corresponding Americas. Third, due to challenges related to how race and ethnicity were reported in vital statistics data for much of the study period, and the absence of information on misclassification for NHPI and Asian populations separately, we did not include separate Americas for NHPI individuals. Previous research in Hawaii has found that NHPI Americans have substantially shorter life expectancy compared with Asian and White Americans.^86^, ^87^ By including NHPI Americans in America 1 (with a much larger Asian population) and America 3 (with a much larger White population), our results mask the disadvantage experienced by this group and likely large inequities between this group and the other Americas. Similarly, AIAN Americans living outside of parts of the West USA (ie, outside of America 10) were also included in America 3. These individuals are also likely to have lower mortality compared with the White population of America 3,^3^ which is masked in our results. Fourth, although this analysis shows that there are large disparities in life expectancy between the ten Americas, it is important to recognise that there are also large disparities within these Americas; for example, previous research has found considerable differences in life expectancy between counties (including counties grouped together in this analysis), even for the same racial and ethnic group,^3^ and it is reasonable to expect further variation within counties.^88^ Fifth, the grouping of county and race and ethnicity combinations into Americas in both the original Eight Americas study and in the present analysis is inherently subjective, and different choices would lead to different results; nonetheless, we strongly believe this is useful as a way of highlighting disparities along several axes yet keeping to a manageable number of groups. Future analyses could build on this framework and consider more structured ways of identifying the different Americas, for example using clustering methodologies.^89^ Finally, although we define the Americas using data from a single point in time, some of the characteristics on which these definitions are based are time-varying (eg, income per capita). Thus, it is likely that some county and race and ethnicity combinations would be included in different Americas, were we to use data from a different year to define the Americas.

The extent and magnitude of health disparities in the USA are truly alarming. In a country with the wealth and resources of the USA, it is intolerable that so many are living in conditions and with health outcomes akin to those of an entirely different country. These disparities reflect the unequal and unjust distribution of resources and opportunities and have profound consequences for the wellbeing and longevity of marginalised populations. It is time for us to take collective action; to invest in equitable health care, education, and employment opportunities; and to challenge the systemic barriers that create and perpetuate these disparities. A comprehensive, coordinated approach that transcends isolated efforts and political divides and fosters collaboration and accountability between state, local, and national entities is required. Only by transcending the current state of the debate can we hope to create a more equitable and healthier society for all the Americas—and all Americans.

### Contributors

### Data sharing

Estimated life expectancy by America, sex, and year are available for download from the Global Health Data Exchange (https://ghdx.healthdata.org/record/ihme-data/us-ten-americas-life-expectancy-disparities-2000-2021). The code used for this analysis is available on GitHub (https://github.com/ihmeuw/USHD).

## Declaration of interests

We declare no competing interests.
