# Supplementary material for: Ten Americas: a systematic analysis of life expectancy disparities in the USA
Source: Lancet. 2024 Dec 7;404(10469):2299–313. doi: 10.1016/S0140-6736(24)01495-8 (PMC11694013; doi:10.1016/S0140-6736(24)01495-8)
Supplement: Supplementary appendix [file mmc1.pdf]

THE LANCET

Supplementary appendix

This appendix formed part of the original submission and has been peer reviewed. We post it as supplied by the authors.

Supplement to: Dwyer-Lindgren L, Baumann MM, Li Z, et al. Ten Americas: a systematic analysis of life expectancy disparities in the USA. *Lancet* 2024; published online Nov 21. https://doi.org/10.1016/S0140-6736(24)01495-8.

Ten Americas: a systematic analysis of life expectancy disparities in the USA

Supplemental Appendix

Contents

GATHER Checklist 3
10 Americas Definitions Map 4
10 Americas Definitions Flow Diagram 5
Data Sources 6
Population Data Processing 9
  Race bridging 9
  Intercensal estimation 9
Life Tables 12
Income and Education Small Area Estimation Models 14
References 20
Supplemental Results 22
  Figure S1: Partial life expectancy, ages 0–4 years, males 22
  Table S1: Partial life expectancy, ages 0–4 years, males 23
  Figure S2: Partial life expectancy, ages 0–4 years, females 25
  Table S2: Partial life expectancy, ages 0–4 years, females 26
  Figure S3: Partial life expectancy, ages 5–24 years, males 28
  Table S3: Partial life expectancy, ages 5–24 years, males 29
  Figure S4: Partial life expectancy, ages 5–24 years, females 31
  Table S4: Partial life expectancy, ages 5–24 years, females 32
  Figure S5: Partial life expectancy, ages 25–44 years, males 34
  Table S5: Partial life expectancy, ages 25–44 years, males 35
  Figure S6: Partial life expectancy, ages 25–44 years, females 37
  Table S6: Partial life expectancy, ages 25–44 years, females 38
  Figure S7: Partial life expectancy, ages 45–64 years, males 40

Table S7: Partial life expectancy, ages 45–64 years, males 41
Figure S8: Partial life expectancy, ages 45–64 years, females........ 43
Table S8: Partial life expectancy, ages 45–64 years, females 44
Figure S9: Partial life expectancy, ages 65–84 years, males........ 46
Table S9: Partial life expectancy, ages 65–84 years, males 47
Figure S10: Partial life expectancy, ages 65–84 years, females 49
Table S10: Partial life expectancy, ages 65–84 years, females 50
Figure S11: Remaining life expectancy, age 85 years, males 52
Table S11: Remaining life expectancy, age 85 years, males 53
Figure S12: Remaining life expectancy, age 85 years, females 55
Table S12: Remaining life expectancy, age 85 years, females 56

GATHER Checklist

Item #	Checklist item	Description of Compliance
Objectives and Funding		
1	Define the indicator(s), populations (including age, sex, and geographic entities), and time period(s) for which estimates were made.	Methods section
2	List the funding sources for the work.	Article information
Data Inputs		
For all data inputs from multiple sources that are synthesized as part of the study:		
3	Describe how the data were identified and how the data were accessed.	Methods section
4	Specify the inclusion and exclusion criteria. Identify all ad-hoc exclusions.	N/A
5	Provide information on all included data sources and their main characteristics. For each data source used, report reference information or contact name/institution, population represented, data collection method, year(s) of data collection, sex and age range, diagnostic criteria or measurement method, and sample size, as relevant.	Methods section, Supplemental Appendix
6	Identify and describe any categories of input data that have potentially important biases (e.g., based on characteristics listed in item 5).	Methods section
For data inputs that contribute to the analysis but were not synthesized as part of the study:		
7	Describe and give sources for any other data inputs.	N/A
For all data inputs:		
8	Provide all data inputs in a file format from which data can be efficiently extracted (e.g., a spreadsheet rather than a PDF), including all relevant meta-data listed in item 5. For any data inputs that cannot be shared because of ethical or legal reasons, such as third-party ownership, provide a contact name or the name of the institution that retains the right to the data.	GHDx
Data Analysis		
9	Provide a conceptual overview of the data analysis method. A diagram may be helpful.	Methods section
10	Provide a detailed description of all steps of the analysis, including mathematical formulae. This description should cover, as relevant, data cleaning, data pre-processing, data adjustments and weighting of data sources, and mathematical or statistical model(s).	Methods section, Supplemental Appendix
11	Describe how candidate models were evaluated and how the final model(s) were selected.	N/A
12	Provide the results of an evaluation of model performance, if done, as well as the results of any relevant sensitivity analysis.	N/A
13	Describe methods for calculating uncertainty of the estimates. State which sources of uncertainty were, and were not, accounted for in the uncertainty analysis.	Methods section
14	State how analytic or statistical source code used to generate estimates can be accessed.	GitHub
Results and Discussion		
15	Provide published estimates in a file format from which data can be efficiently extracted.	GHDx
16	Report a quantitative measure of the uncertainty of the estimates (e.g. uncertainty intervals).	Results section
17	Interpret results in light of existing evidence. If updating a previous set of estimates, describe the reasons for changes in estimates.	Introduction, Discussion sections
18	Discuss limitations of the estimates. Include a discussion of any modelling assumptions or data limitations that affect interpretation of the estimates.	Discussion section

10 Americas Definitions Map

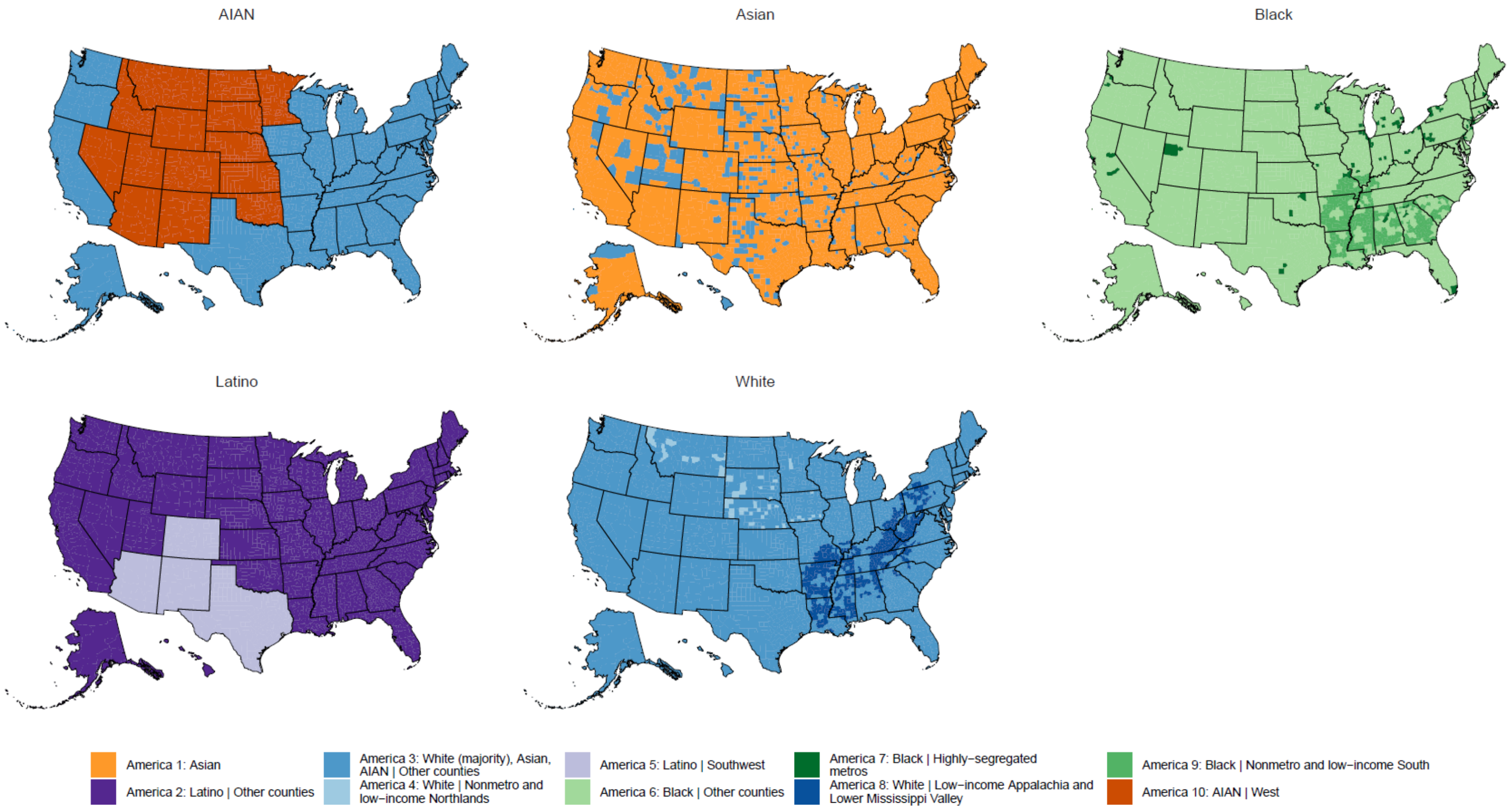

10 Americas Definitions Flow Diagram

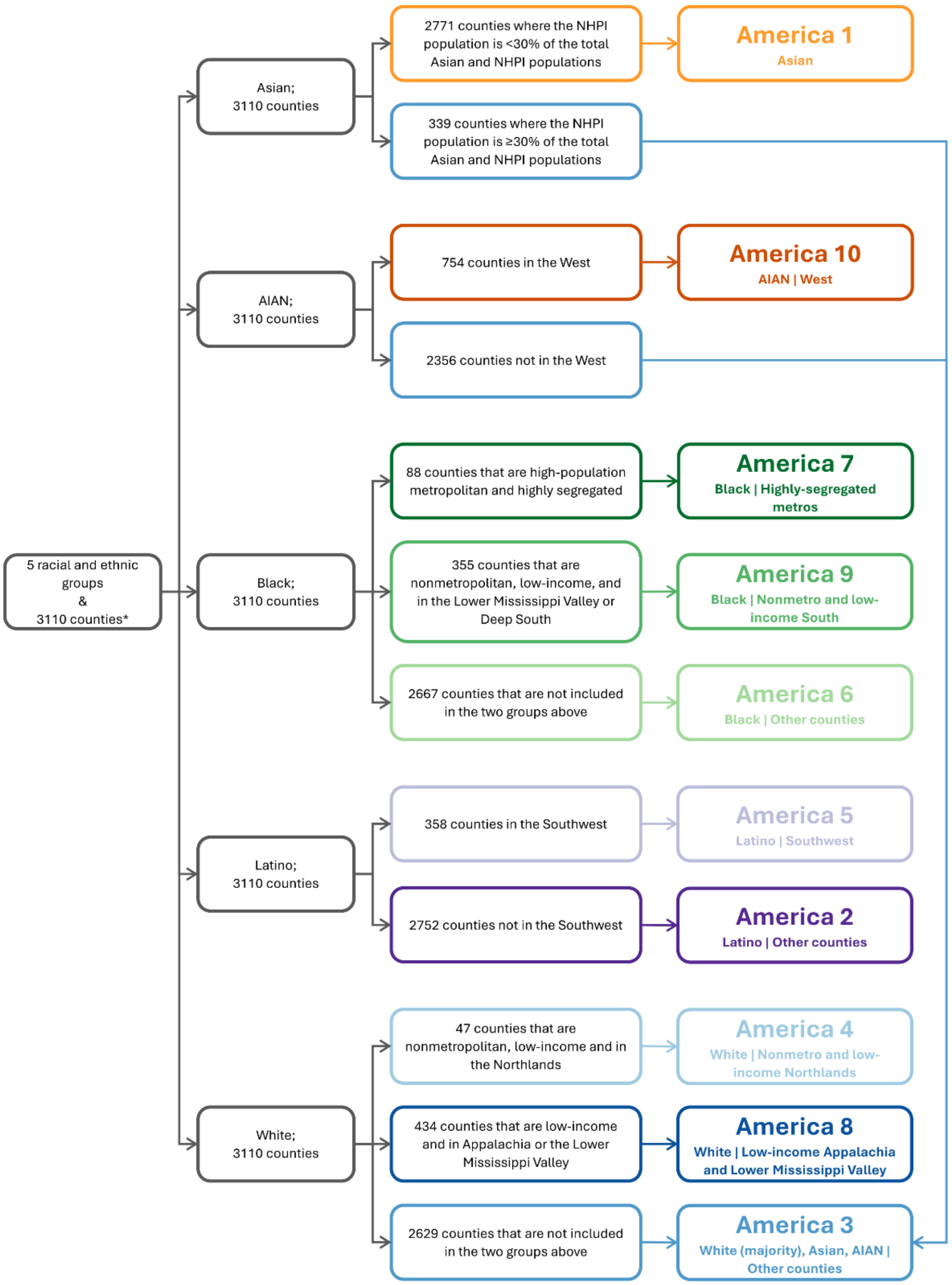

*Some county boundaries changed during the study period. To create historically stable geographical units for analysis, a small number of counties were combined. This reduces the number of units analyzed from 3143 counties to 3110 counties or groups of counties.

Data Sources

Variable	Source(s)	Data Processing	Citations
Proportion of the Asian and NHPI population that is NHPI	2020 Decennial Census Table P2 [1]	Proportion defined as Asian Alone or in Combination / (Asian Alone or in Combination + NHPI Alone or in combination).	[1] U.S. Census Bureau. "HISPANIC OR LATINO, AND NOT HISPANIC OR LATINO BY RACE." Decennial Census, DEC Redistricting Data (PL 94-171), Table P2, 2020, https://data.census.gov/table/DECENNIALPL2020.P2?g=160XX00US2228345. Accessed on January 7, 2024.
Per capita income	2000 Census [2]; 2009–22 ACS [3]; 2000–22 Bureau of Labor Statistics Consumer Price Index [4]	ACS estimates for the AIAN, Asian, Black, and NHPI populations were not available stratified by Latino ethnicity and were used as proxies for non-Latino AIAN, non-Latino Asian, non-Latino Black, and non-Latino NHPI estimates, respectively. Imputation via a small area estimation model (described below) was used to smooth the data and impute missing values.	[2] Minnesota Population Center. 2000 Census Summary File 4, Table NPCT130A. IPUMS National Historical Geographic Information System: Version 15.0. Minneapolis, MN: IPUMS 2020. https://www.nhgis.org/. Accessed October 8, 2020.<br><br>[3] US Census Bureau. American Community Survey, 2009–22 American Community Survey 5-Year Estimates, Tables B19301A-B19301I; using Census data portal; https://data.census.gov/cedsci/. Accessed January 7, 2024.<br><br>[4] US Bureau of Labor Statistics. Consumer Price Index: All Urban Consumers History, All Items 1913–2023. https://www.bls.gov/data/. Accessed March 26, 2024.

Variable	Source(s)	Data Processing	Citations
		Data were adjusted for inflation using the consumer price index. County-level estimates for all racial and ethnic populations combined were generated by calculating the population-weighted average of the race and ethnicity specific estimates within each county.	
Metropolitan	2013 Rural-Urban Continuum Codes [5]; 2023 Rural-Urban Continuum Codes [6]	Metropolitan counties were defined as counties with RUCC code 1 ("Counties in metro areas of 1 million population or more"). Nonmetropolitan counties were defined as counties with RUCC codes 4–9. Connecticut counties used the 2013 RUCC codes due to the change from	[5] U.S. Department of Agriculture, Economic Research Service. Rural-Urban Continuum Codes 2013. January 2024.<br><br>[6] U.S. Department of Agriculture, Economic Research Service. Rural-Urban Continuum Codes 2023. January 2024.

Variable	Source(s)	Data Processing	Citations
		counties to planning regions, all other counties used the 2023 RUCC codes.	
Black-white residential segregation	2024 County Health Rankings & Road Maps [7]	Highly segregated counties defined as counties having a Black-White residential segregation index of 60 or higher.	[7] University of Wisconsin Population Health Institute. County Health Rankings & Roadmaps 2024. 2024 County Health Release National Data. www.countyhealthrankings.org.
Proportion of the population with a HS diploma or college degree	2000 census [8]; 2010–20 ACS [9]	ACS estimates for the AIAN, Asian, Black, and NHPI populations were not available stratified by Latino ethnicity and were used as proxies for non-Latino AIAN, non-Latino Asian, non-Latino Black, and non-Latino NHPI estimates, respectively. Imputation via a small area estimation model was used to smooth data and impute missing values.	[8] Minnesota Population Center. 2000 Census Summary File 4, Table NPCT064C. IPUMS National Historical Geographic Information System: Version 15.0. Minneapolis, MN: IPUMS 2020. https://www.nhgis.org/. Accessed August 25, 2020.<br><br>[9] US Census Bureau. American Community Survey, 2010–20 American Community Survey 5-Year Estimates, Tables C15002A–C15002I; using Census data portal; https://data.census.gov/cedsci/. Accessed October 5, 2022.

Population Data Processing

For this analysis, we needed population estimates by single (bridged) race for 2000–21. Intercensal bridged-race estimates for 2000–09 are available directly from the National Center for Health Statistics (NCHS),[1] as are postcensal bridged-race estimates for 2010-19 (based on Census 2010),[2] which we adjusted to approximate the intercensal estimates for this time span; our methodology for this is outlined below. For 2020–21 we use the US Census Bureau's Census Population Estimates Program (PEP) postcensal estimates (based on Census 2020)[3] but perform our own race-bridging to align them with the 1977 OMB standard racial and ethnic groups;[4] this process is also detailed below.

Race bridging

As of 2021, NCHS stopped producing bridged-race population estimates. To fill this gap, we used population information from 2020 for bridged racial and ethnic groups to derive such estimates for 2021.

Using the bridged-race estimates from 2020 and the 2020 Census PEP estimates, we determined the number of non-Latino multiracial individuals who were bridged to any given single-race group (eg, subtracting the Black alone population estimate from the Black bridged population estimate gives the number of people who identified as multiracial and were bridged to Black). For each county-age-sex-year stratum, we converted this number to a proportion of the total multiracial population, which we multiplied by the 2021 "Two or more races" population estimates, giving an approximate breakdown of the single-race totals within this group. Finally, we added these estimates to their respective single-race population estimates.

For strata where the total multiracial population was 0, we successively pooled observations until proportions could be determined; strata were first aggregated to combine males and females, followed by 20-year age groups, all ages, then states.

Intercensal estimation

Because the Census Bureau has not yet released 2010–19 intercensal population estimates, we approximated them using the documented methodology for calculating the 2000–10 estimates.[5] We made this adjustment after race-bridging the 2021 Census population estimates so the strata between

2020 and 2021 would properly align. In most cases, we used a modification of the Das Gupta method, a function of time and postcensal estimates:

$$P_t = Q_t(P_{3653}/Q_{3653})^{(t-3653)}$$

where t = time in days since July 1, 2010;

P_t = intercensal population estimate at time t;

Q_t = postcensal estimate at time t based on Census 2010;

P_{3653} = July 1, 2020 postcensal estimate based on Census 2020; and

Q_{3653}= July 1, 2020 postcensal estimate based on Census 2010.

The postcensal estimates based on Census 2010 do not include the April 2020 Census count, leading us to revise the Census method slightly and substitute July postcensal estimates for P_{3653} and Q_{3653}. Additionally, there was an extra leap day that occurred between 2010 and 2020, compared to 2000 to 2010, so we incremented the maximum value of t to be 3653 instead of 3652.

For some edge cases, the above method does not work. Consistent with the Census Bureau's approach in these cases, we used linear interpolation for observations that met at least one of the below criteria:

$Q_t = 0$; or

$Q_{3653} = 0 \ or \ 1$; or

$P_{3653} = 0 \ or \ 1$; or

$Q_{3653} < 0.5 \cdot P_{3653}$

The equation for these cases is:

$$P_t = P_{3653} \cdot \left(\frac{t}{3653}\right) + P_0 \cdot \left(\frac{3653-t}{3653}\right),$$

where t = time in days since July 1, 2010;

P_t = intercensal population estimate at time t;

P_{3653} = July 1, 2020 postcensal estimate based on Census 2020; and

P_0 = July 1, 2010 postcensal estimate based on Census 2010.

Using postcensal information based on Census 2020 in our approximation of the 2010–19 intercensal estimates resulted in more realistic population trends between 2019 and 2020–21, mitigating the discontinuities we previously observed during this time period.

Life Tables

Life tables describe the distribution of deaths by age for a cohort of individuals. In a period life table, this cohort is entirely hypothetical: we consider what would happen to a cohort of individuals born in a given year, *if* the age-specific mortality rates occurring in that year were constant throughout the cohort's existence. As age-specific mortality rates are rarely constant over long periods of time, life expectancy at birth from period life table should not be interpreted as a prediction of how long individuals born in a given year will live, but rather as a way of summarizing mortality conditions at a given point in time.

A detailed explanation of how to construct an abridged period life table, including the intuition behind each step, is available from Preston et al.[6] Here, we briefly summarize how we applied these calculations in this analysis:

1. We used the age-specific mortality rates calculated using the procedure described in the main text. In demographic notation, these are denoted ${}_nm_x$, where x is the beginning of the age interval and n is the length of the age interval (eg, ${}_5m_{15}$ is the age-specific mortality rate for age group 15–19).
2. We estimated the mean number of person-years lived in each age interval by individuals in the hypothetical cohort dying in the interval (${}_na_x$) using a three step-process. First, we calculated ${}_na_x$ using the formulas described by Preston et al.[6] (Table 3.3, p 48) for the infant (age 0) and childhood (age 1–4) age intervals, and we set ${}_na_x$ equal to $n/2$, ie, half the length of the age interval, for other age intervals. Second, we calculated the probability of dying in each age interval (${}_nq_x$) as:

$${}_nq_x = \frac{n \cdot {}_nm_x}{1 + (n - {}_na_x) \cdot {}_nm_x}$$

   Third, we used a process called graduation to revise the estimates of ${}_na_x$ and ${}_nq_x$ for most age groups; this process provides improved estimates of ${}_na_x$ based on the observed slope of ${}_nm_x$.[7]
3. We calculated the proportion of the hypothetical cohort surviving to age x (l_x) as:

$$l_0 = 1$$

$$l_{x+n} = l_x \cdot (1 - {}_nq_x)$$

4. We calculated the number of deaths in the hypothetical cohort occurring in each age interval (${}_nd_x$) as:

$${}_nd_x = l_x \cdot {}_nq_x$$

5. For all age groups except the terminal age group (85+), we calculated the number of person-years lived in the interval by the hypothetical cohort (${}_nL_x$) as:

$$ {}_nL_x = n \cdot l_{x+n} + {}_na_x \cdot {}_nd_x $$

6. For the terminal age group (85+) only, we calculated life expectancy (e_{85}) using the method proposed by Horiuchi and Coale:[8]

$$ e_{85} = \left(\frac{1}{{}_\infty m_{85}}\right) \cdot \exp(-\beta_{85} \cdot r \cdot {}_\infty m_{85}{}^{-\alpha_{85}}) $$

   where $\beta_{85} = 0.095$ and $\alpha_{85} = 1.4$, and r is the population growth rate for the 85+ age group calculated as the mean of the annual growth rates over the preceding ten years. We then back-calculated ${}_\infty L_{85}$ as:

$$ {}_\infty L_{85} = e_{85} \cdot l_{85} $$

7. We calculated the number of person-years lived in above age x in the hypothetical cohort (T_x) as:

$$ T_x = \Sigma_{a=x}^{\infty} {}_nL_x $$

8. Finally, we calculated life expectancy at each age x in the hypothetical cohort (e_x) as:

$$ e_x = T_x / l_x $$

We are able to extract life expectancy at birth (e_0), and life expectancy at age 85 (e_{85}) directly from the lifetables. We calculated partial life expectancy for broader age groups 0–4, 5–24, 25–44, 45–64, and 65–84 as the sum of the ${}_nL_x$ values for each of the shorter age ranges within the broader group, divided by l_x for the first age within the broader group. For example, partial life expectancy for age group 5–24 was calculated as:

$$ {}_{20}e_5 = ({}_5L_5 + {}_5L_{10} + {}_5L_{15} + {}_5L_{20}) / l_5 $$

Income and Education Small Area Estimation Models

We used small area estimation models to estimate income per capita, the percentages of the age 25+ population that have obtained a high school diploma (HS grads), and the percentage of the age 25+ population that have obtained a bachelor's degree or higher (college grads), using tabulated data from the 2000 decennial population census and the American Community Survey (ACS, 2007–20).

Educational Attainment

Estimates and Standard Errors for Decennial Census Data (2000)

We aggregated census data on educational attainment across sex to obtain summed counts (populations with a given education level and their total population denominators) for 2000 by county and racial and ethnic population. We similarly combined estimates for the Asian and NHPI populations using summed counts.

The long-form decennial census questionnaire was completed by a 17% sample of the population in 2000.[9] We therefore assumed sample sizes ($\widehat{\mathrm{N}}$) to be 17% of the total population for each combination of county, year, and racial and ethnic population in 2000. As the decennial census data did not include estimates of uncertainty, we derived standard errors for the 2000 educational attainment data using the relationship among estimated sample size, the population proportion, $\hat{p}$, and its standard error, $\mathrm{SE}(\hat{p})$:[10]

$$\mathrm{SE}(\hat{p}) = \sqrt{\frac{\hat{p}(1-\hat{p})}{\widehat{\mathrm{N}}}} \quad (1)$$

As strata with observed proportions of 0 or 1 produce standard error estimates of 0, we first transformed the reported population fractions ($\hat{p}$) by an empirical logit transformation,[11] and then inverse-transformed by use of the standard inverse logit function, prior to calculating $\mathrm{SE}(\hat{p})$ for these strata:

$$\hat{p}_{emp} = \mathrm{logit}^{-1}\left(\log\left(\frac{\hat{p}+\frac{\varepsilon}{2}}{1-\hat{p}+\frac{\varepsilon}{2}}\right)\right) \quad (2)$$

where $\hat{p}_{emp}$ is the empirical logit-transformed proportion, $\mathbf{logit}^{-1}$ is the standard inverse logit function, and ε is the smallest absolute difference from zero or one among proportions in the data set.

Estimates and Standard Errors for American Community Survey Data (2007–2020)

ACS educational attainment counts for years 2007–20 were aggregated as described above for the 2000 census data. We obtained margins of error (MOEs) for each variable, which ACS provides at a confidence level of 90%. Per guidance from the Census Bureau,[12] we calculated variances as:

$$\mathrm{Var}(\widehat{\mathrm{X}}) = \left(\frac{\mathrm{MOE}(\widehat{\mathrm{X}})}{1.645}\right)^2 \tag{3}$$

where $\widehat{\mathrm{X}}$ is the estimated count from ACS. We calculated the variances for summed values (ie, when combining sexes, educational attainment groups, or the Asian and NHPI populations) as the sum of the corresponding variances, assuming independence:

$$\mathrm{Var}(\widehat{\mathrm{X}}_1 + \widehat{\mathrm{X}}_2) = \mathrm{Var}(\widehat{\mathrm{X}}_1) + \mathrm{Var}(\widehat{\mathrm{X}}_2) \tag{4}$$

We then back-transformed the combined variances for educational attainment to MOEs using the inverse of equation 3.

We next calculated the MOEs of the educational attainment proportions, following published guidance from the Census Bureau:

$$\mathrm{MOE}(\hat{p}) = \frac{1}{\widehat{\mathrm{Y}}}\left(\sqrt{[\mathrm{MOE}(\widehat{\mathrm{X}})]^2 - \hat{p}^2[\mathrm{MOE}(\widehat{\mathrm{Y}})]^2}\right) \tag{5}$$

where $\widehat{\mathrm{X}}$ is the ACS estimate of individuals with a given level of educational attainment, $\widehat{\mathrm{Y}}$ is the ACS estimate of total population for the stratum, and $\hat{p}$ is the proportion of individuals with that level of educational attainment (ie, $\widehat{\mathrm{X}}$ / $\widehat{\mathrm{Y}}$). We used equation 5 to derive MOEs for proportions of HS grads and college grads and then calculated variances and standard errors from these MOEs as in equation 3.

Small Area Estimation of Education Proportions

We modelled educational attainment proportions (separately for HS grads and college grads) using small area estimation models in R-INLA v.24.02.09[13] and R v4.2.2:[14]

$$\hat{p}_{j,t,r} \sim \text{Normal}\left(\eta_{j,t,r}, \sigma^2 \cdot \text{Var}\left(\hat{p}_{j,t,r}\right)\right) \tag{6}$$

$$\eta_{j,t,r} = \boldsymbol{\beta}_0 + \gamma_{1,r} + \gamma_{2,j} + \gamma_{3,t,r} + \gamma_{4,j,t,r} \tag{7}$$

where $\hat{p}_{j,r,t}$ is the proportion of the population in county *j,* racial and ethnic population *r*, and year *t*, that has graduated high school or college. Model terms consist of:

- σ^2 is the variance of the error term, scaled for model-fitting by the variance estimated for each stratum;
- $\boldsymbol{\beta}_0$ is a global intercept;
- $\gamma_{1,r}$ is a random intercept for race, with an independent-and-identically-distributed (IID) Gaussian prior;
- $\gamma_{2,j}$ is a random intercept for county with a Besag-York-Mollie-type prior (BYM2), combining a conditional autoregressive distribution for spatial autocorrelation, based on county adjacency, with an IID Gaussian prior;
- $\gamma_{3,t,r}$ and $\gamma_{4,j,t,r}$ are second-order autoregressive (AR[2]) terms for year, replicated by race or by race and county, respectively.

We used a relatively uninformative Normal(mean = 0.0, precision = 0.1) prior for the global intercept. We used default INLA hyperpriors for the hyperparameters because we considered them suitably uninformative. The BYM2 parameterisation includes a parameter, φ, that indicates the contribution of the structured spatial effect to the marginal variance; $\mathbf{logit}(\varphi)$ had a penalised complexity (PC) prior corresponding to $\Pr(\varphi < 0.5) = 0.5$, and the log precision of the BYM2 model had a PC prior corresponding to $\Pr(\sigma > 1.0) = 0.1$. The IID term had a Gamma(shape = 1.0, inverse-scale = 5×10^{-5}) prior on the log precision. The AR(2) terms had PC priors corresponding to $\Pr(\sigma > 3.0) = 0.01$ for the log precision, $\Pr(\rho_1 > 0.5) = 0.5$ for the 1-year lagged correlation (ρ_1) and $\Pr(\rho_2 > 0.5) = 0.4$ for the 2-year lagged correlation (ρ_2). A Gaussian approximation strategy, empirical Bayes integration strategy, and step-length for hyperparameter gradient calculations of 1×10^{-3} were used for model fitting. The default Low-Rank Variational Bayes correction[15] was turned off for the AR(2) terms due to excessive memory requirements. In comparisons using data for a subset of states, this correction had no noticeable impact on model predictions.

Income per Capita

Estimates and Standard Errors for Decennial Census Data (2000)

We combined census data for the Asian and NHPI populations, and derived population-weighted mean income per capita by county and racial and ethnic population. Income per capita was adjusted for inflation to year 2022 dollars. As with educational attainment, we assumed sample sizes to be 17% of the total population for each combination of county and racial and ethnic population in 2000.

In order to obtain uncertainty estimates for the 2000 income data, we modelled standard deviations of income per capita on the log scale using a generalized linear mixed model in R-INLA, where the model was trained on the 2007–20 income per capita data from ACS (see next section):

$$\mathrm{SD}_{j,t,r}(\log(\hat{x})) \sim \mathrm{Normal}(\eta_{j,t,r}, \sigma^2) \tag{8}$$

$$\eta_{j,t,r} = \boldsymbol{\beta}_0 + \gamma_1 + \gamma_{2,j,r} + \gamma_{3,j,t,r} \tag{9}$$

where $\mathrm{SD}_{j,t,r}(\log(\hat{x}))$ is the standard deviation of log income per capita for county *j* and racial and ethnic population *r*. Model terms differed from the education small area INLA models in the following ways (otherwise as described for those models):

- γ_1 is a second-order random walk (RW2) model on income per capita to accommodate non-linearity in the relationship between income per capita and its standard deviation on the log scale; income is discretized into 25 bins of equal interval, and effects were constrained to sum to 0;
- $\gamma_{2,j,r}$ and $\gamma_{3,j,t,r}$ are random intercepts for county and race or county, year and race, respectively, with IID Gaussian priors.

The priors and model-fitting approach were as described previously for the education INLA models, with the addition of a Gamma(shape = 1.0, inverse-scale = 5×10^{-5}) prior on the log precision of the RW2 term. Mean predictions from this model were used to estimate the SE of log income per capita for the year 2000 census data, as:

$$\mathrm{SE}(\log(\hat{x})) = \frac{\mathrm{SD}(\log(\hat{x}))}{\sqrt{\hat{\mathrm{N}}}} \tag{10}$$

Estimates and Standard Errors for American Community Survey Data (2007–2020)

Analogously with the decennial census data, we derived estimated sample sizes for ACS income data by applying the national annual sampling rates for the ACS survey to the total population for each stratum. We derived MOEs for combined groups (combining Asian and NHPI populations) as for the educational attainment covariates, except that the calculation of MOEs for proportions was not relevant for income.

Standard errors for log income per capita were produced for ACS data via delta transformation of the standard errors for the untransformed income estimates:

$$\mathrm{SE}(\log(\hat{x})) = \sqrt{\left(\frac{1}{\hat{x}}\right)^2 \mathrm{SE}(\hat{x})} \tag{11}$$

where $\hat{x}$ is income per capita, $\mathrm{SE}(\hat{x})$ is the standard error of income on the untransformed scale, and $\mathrm{SE}(\log(\hat{x}))$ is the standard error in log space. We then estimated standard deviations of log income as:

$$\mathrm{SD}(\log(\hat{x})) = \mathrm{SE}(\log(\hat{x}))\sqrt{\widehat{\mathrm{N}}} \tag{12}$$

where $\widehat{\mathrm{N}}$ is the estimated sample size. These standard deviation estimates were used to estimate standard deviations for the 2000 census income data (see previous section).

Small Area Estimation of Income per Capita

We modelled income per capita in R-INLA using a small area model that was similar to those used for educational attainment:

$$\log(\hat{x}_{j,t,r}) \sim \mathrm{Normal}\big(\eta_{j,t,r}, \sigma^2 \cdot \mathrm{Var}(\log(\hat{x}))\big) \tag{13}$$

$$\eta_{j,t,r} = \boldsymbol{\beta}_0 + \gamma_{1,r} + \gamma_{2,j} + \gamma_{3,t} + \gamma_{4,t,r} + \gamma_{5,j,t,r} \tag{14}$$

where $\log(\hat{x}_{j,t,r})$ is log-transformed income per capita for county *j,* year *t*, and racial and ethnic population *r*. Model terms are as described for educational attainment, with the following differences:

- the Gaussian precision was scaled during model-fitting by the $\mathrm{Var}(\log(\hat{x}))$ values estimated for each stratum;
- $\gamma_{3,t}$ is a second-order random walk model (RW2) for year;

- $\gamma_{4,t,r}$ is a second-order random walk model (RW2) for year, replicated by race;
- $\gamma_{5,j,t,r}$ is a second-order autoregressive model (AR[2]) for year, replicated by race and county.

The priors and model-fitting approach were as described previously for the education INLA models. The prior on the RW2 terms was as in the income standard deviation model.

References

1 National Center for Health Statistics, Centers for Disease Control and Prevention, US Census Bureau. United States Bridged-Race Intercensal Population Estimates 2000-2009. Hyattsville, United States: National Center for Health Statistics, Centers for Disease Control and Prevention, 2012 https://www.cdc.gov/nchs/nvss/bridged_race.htm (accessed Oct 30, 2012).

2 National Center for Health Statistics, Centers for Disease Control and Prevention, US Census Bureau. United States Vintage 2020 Bridged-Race Postcensal Population Estimates 2010-2020. Hyattsville, United States: National Center for Health Statistics, Centers for Disease Control and Prevention, 2020 https://www.cdc.gov/nchs/nvss/bridged_race.htm (accessed Feb 17, 2022).

3 US Census Bureau (2022). United States Population and Housing Unit Postcensal Estimates, Vintage 2021. Census.gov. https://www.census.gov/data/datasets/time-series/demo/popest/2020s-counties-detail.html (accessed Dec 12, 2022).

4 Office of Management and Budget. Race and ethnic standards for federal statistics and administrative reporting: OMB Directive No. 15. Washington DC, 1977 https://wonder.cdc.gov/wonder/help/populations/bridged-race/directive15.html (accessed Oct 25, 2023).

5 US Census Bureau. Methodology for the Intercensal Population and Housing Unit Estimates: 2000 to 2010. 2012. www2.census.gov/programs-surveys/popest/technical-documentation/methodology/intercensal/2000-2010-intercensal-estimates-methodology.pdf (accessed Nov 17, 2023).

6 Preston S, Heuveline P, Guillot M. Demography: measuring and modeling population processes. Malden, MA: Blackwell Publishers, 2001.

7 Keyfitz N. A life table that agrees with the data. *J Am Stat Assoc* 1966; **61**: 305–12.

8 Horiuchi S, Coale AJ. A simple equation for estimating the expectation of life at old ages. *Popul Stud* 1982; **36**: 317–26.

9 US Census Bureau CHS. Overview- History- U.S. Census Bureau. https://www.census.gov/history/www/through_the_decades/overview/ (accessed Feb 27, 2023).

10 Franco C, Little RJA, Louis TA, Slud EV. Comparative study of confidence intervals for proportions in complex sample surveys. *J Surv Stat Methodol* 2019; **7**: 334–64.

11 Warton DI, Hui FKC. The arcsine is asinine: the analysis of proportions in ecology. *Ecology* 2011; **92**: 3–10.

12 US Census Bureau. Understanding and Using American Community Survey Data: What All Data Users Need to Know. Washington DC: US Government Publishing Office, 2020 https://www.census.gov/programs-surveys/acs/library/handbooks/general.html (accessed May 6, 2024).

13 Rue H, Martino S, Chopin N. Approximate Bayesian inference for latent Gaussian models by using integrated nested Laplace approximations. *J R Stat Soc Ser B Stat Methodol* 2009; **71**: 319–92.

14 R Core Team. A language and environment for statistical computing. 2024. https://www.R-project.org/.

15 Niekerk J, Rue H. Low-rank Variational Bases correction to the Laplace method. stat.ME. 2023. https://arxiv.org/pdf/2111.12945.

Supplemental Results

Figure S1: Partial life expectancy, ages 0–4 years, males

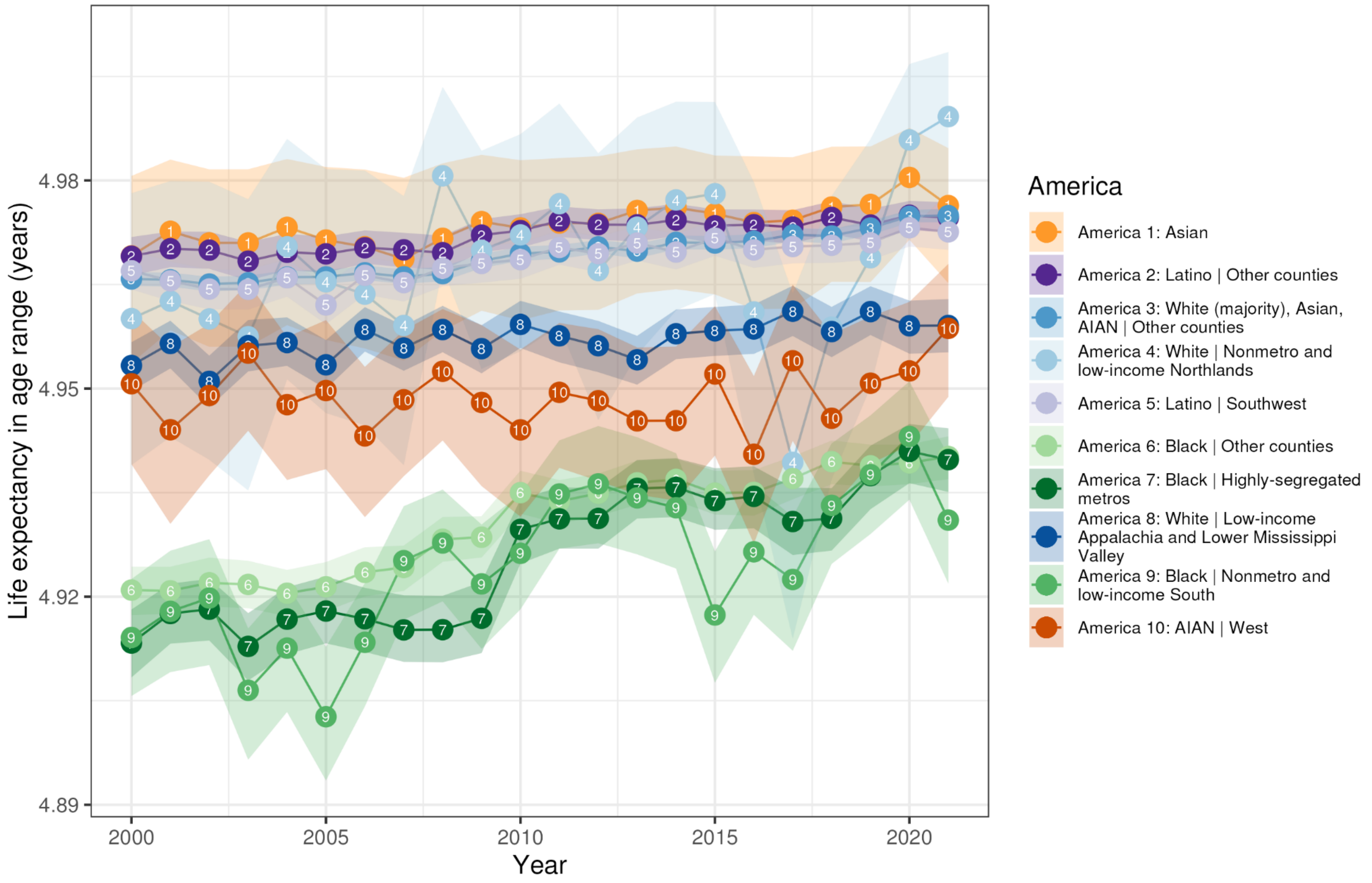

Table S1: Partial life expectancy, ages 0–4 years, males

America	Life expectancy, 2000	Change, 2000–10	Life expectancy, 2010	Change, 2010–19	Life expectancy, 2019	Change, 2019–20	Life expectancy, 2020	Change, 2020–21	Life expectancy, 2021	Change, 2000–19	Change, 2019–21
America 1: Asian	5.0 (5.0–5.0)	0.0 (0.0 to 0.0)*	5.0 (5.0–5.0)	0.0 (0.0 to 0.0)*	5.0 (5.0–5.0)	0.0 (0.0 to 0.0)*	5.0 (5.0–5.0)	0.0 (0.0 to 0.0)*	5.0 (5.0–5.0)	0.0 (0.0 to 0.0)*	0.0 (0.0 to 0.0)
America 2: Latino | Other counties	5.0 (5.0–5.0)	0.0 (0.0 to 0.0)*	5.0 (5.0–5.0)	0.0 (0.0 to 0.0)	5.0 (5.0–5.0)	0.0 (0.0 to 0.0)	5.0 (5.0–5.0)	0.0 (0.0 to 0.0)	5.0 (5.0–5.0)	0.0 (0.0 to 0.0)*	0.0 (0.0 to 0.0)
America 3: White (majority), Asian, AIAN | Other counties	5.0 (5.0–5.0)	0.0 (0.0 to 0.0)*	5.0 (5.0–5.0)	0.0 (0.0 to 0.0)*	5.0 (5.0–5.0)	0.0 (0.0 to 0.0)*	5.0 (5.0–5.0)	0.0 (0.0 to 0.0)	5.0 (5.0–5.0)	0.0 (0.0 to 0.0)*	0.0 (0.0 to 0.0)*
America 4: White | Nonmetro and low-income Northlands	5.0 (4.9–5.0)	0.0 (0.0 to 0.0)	5.0 (5.0–5.0)	0.0 (0.0 to 0.0)	5.0 (5.0–5.0)	0.0 (0.0 to 0.0)	5.0 (5.0–5.0)	0.0 (0.0 to 0.0)	5.0 (5.0–5.0)	0.0 (0.0 to 0.0)	0.0 (0.0 to 0.0)*
America 5: Latino | Southwest	5.0 (5.0–5.0)	0.0 (0.0 to 0.0)	5.0 (5.0–5.0)	0.0 (0.0 to 0.0)	5.0 (5.0–5.0)	0.0 (0.0 to 0.0)	5.0 (5.0–5.0)	0.0 (0.0 to 0.0)	5.0 (5.0–5.0)	0.0 (0.0 to 0.0)*	0.0 (0.0 to 0.0)
America 6: Black | Other counties	4.9 (4.9–4.9)	0.0 (0.0 to 0.0)*	4.9 (4.9–4.9)	0.0 (0.0 to 0.0)*	4.9 (4.9–4.9)	0.0 (0.0 to 0.0)	4.9 (4.9–4.9)	0.0 (0.0 to 0.0)	4.9 (4.9–4.9)	0.0 (0.0 to 0.0)*	0.0 (0.0 to 0.0)
America 7: Black | Highly-segregated metros	4.9 (4.9–4.9)	0.0 (0.0 to 0.0)*	4.9 (4.9–4.9)	0.0 (0.0 to 0.0)*	4.9 (4.9–4.9)	0.0 (0.0 to 0.0)	4.9 (4.9–4.9)	0.0 (0.0 to 0.0)	4.9 (4.9–4.9)	0.0 (0.0 to 0.0)*	0.0 (0.0 to 0.0)
America 8: White | Low-income Appalachia and Lower	5.0 (4.9–5.0)	0.0 (0.0 to 0.0)*	5.0 (5.0–5.0)	0.0 (0.0 to 0.0)	5.0 (5.0–5.0)	0.0 (0.0 to 0.0)	5.0 (5.0–5.0)	0.0 (0.0 to 0.0)	5.0 (5.0–5.0)	0.0 (0.0 to 0.0)*	0.0 (0.0 to 0.0)

Mississippi Valley											
America 9: Black | Nonmetro and low-income South	4.9 (4.9–4.9)	0.0 (0.0 to 0.0)*	4.9 (4.9–4.9)	0.0 (0.0 to 0.0)	4.9 (4.9–4.9)	0.0 (0.0 to 0.0)	4.9 (4.9–5.0)	0.0 (0.0 to 0.0)	4.9 (4.9–4.9)	0.0 (0.0 to 0.0)*	0.0 (0.0 to 0.0)
America 10: AIAN | West	5.0 (4.9–5.0)	0.0 (0.0 to 0.0)	4.9 (4.9–5.0)	0.0 (0.0 to 0.0)	5.0 (4.9–5.0)	0.0 (0.0 to 0.0)	5.0 (4.9–5.0)	0.0 (0.0 to 0.0)	5.0 (4.9–5.0)	0.0 (0.0 to 0.0)	0.0 (0.0 to 0.0)

Numbers in parentheses are 95% uncertainty intervals.
*Indicates that the uncertainty bounds do not encompass 0.

Figure S2: Partial life expectancy, ages 0–4 years, females

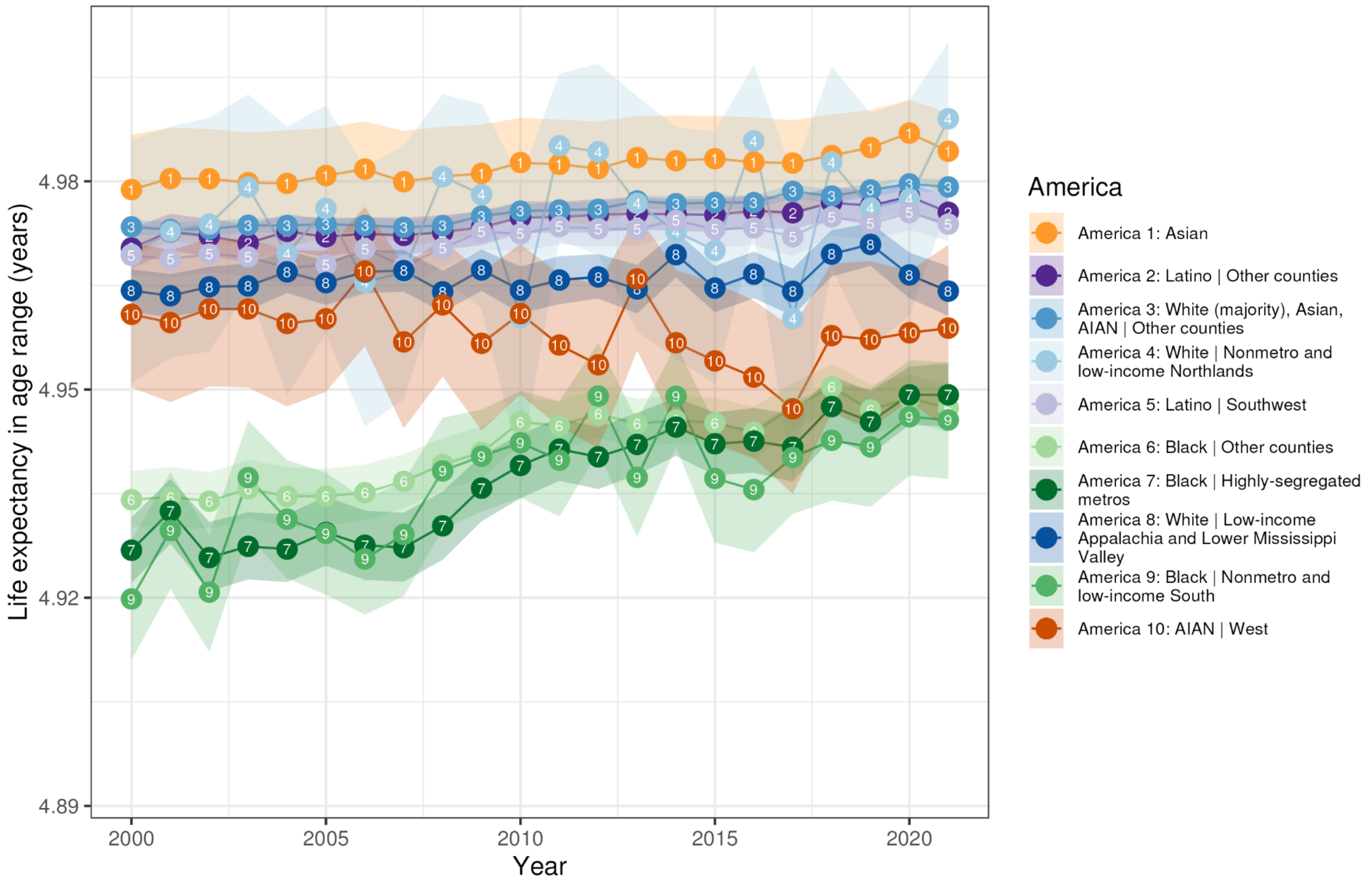

Table S2: Partial life expectancy, ages 0–4 years, females

America	Life expectancy, 2000	Change, 2000–10	Life expectancy, 2010	Change, 2010–19	Life expectancy, 2019	Change, 2019–20	Life expectancy, 2020	Change, 2020–21	Life expectancy, 2021	Change, 2000–19	Change, 2019–21
America 1: Asian	5.0 (5.0–5.0)	0.0 (0.0 to 0.0)*	5.0 (5.0–5.0)	0.0 (0.0 to 0.0)	5.0 (5.0–5.0)	0.0 (0.0 to 0.0)	5.0 (5.0–5.0)	0.0 (0.0 to 0.0)*	5.0 (5.0–5.0)	0.0 (0.0 to 0.0)*	0.0 (0.0 to 0.0)
America 2: Latino | Other counties	5.0 (5.0–5.0)	0.0 (0.0 to 0.0)*	5.0 (5.0–5.0)	0.0 (0.0 to 0.0)*	5.0 (5.0–5.0)	0.0 (0.0 to 0.0)	5.0 (5.0–5.0)	0.0 (0.0 to 0.0)*	5.0 (5.0–5.0)	0.0 (0.0 to 0.0)*	0.0 (0.0 to 0.0)
America 3: White (majority), Asian, AIAN | Other counties	5.0 (5.0–5.0)	0.0 (0.0 to 0.0)*	5.0 (5.0–5.0)	0.0 (0.0 to 0.0)*	5.0 (5.0–5.0)	0.0 (0.0 to 0.0)	5.0 (5.0–5.0)	0.0 (0.0 to 0.0)	5.0 (5.0–5.0)	0.0 (0.0 to 0.0)*	0.0 (0.0 to 0.0)
America 4: White | Nonmetro and low-income Northlands	5.0 (5.0–5.0)	0.0 (0.0 to 0.0)	5.0 (4.9–5.0)	0.0 (0.0 to 0.0)	5.0 (5.0–5.0)	0.0 (0.0 to 0.0)	5.0 (5.0–5.0)	0.0 (0.0 to 0.0)	5.0 (5.0–5.0)	0.0 (0.0 to 0.0)	0.0 (0.0 to 0.0)
America 5: Latino | Southwest	5.0 (5.0–5.0)	0.0 (0.0 to 0.0)*	5.0 (5.0–5.0)	0.0 (0.0 to 0.0)	5.0 (5.0–5.0)	0.0 (0.0 to 0.0)	5.0 (5.0–5.0)	0.0 (0.0 to 0.0)	5.0 (5.0–5.0)	0.0 (0.0 to 0.0)*	0.0 (0.0 to 0.0)
America 6: Black | Other counties	4.9 (4.9–4.9)	0.0 (0.0 to 0.0)*	4.9 (4.9–4.9)	0.0 (0.0 to 0.0)	4.9 (4.9–5.0)	0.0 (0.0 to 0.0)	4.9 (4.9–5.0)	0.0 (0.0 to 0.0)	4.9 (4.9–5.0)	0.0 (0.0 to 0.0)*	0.0 (0.0 to 0.0)
America 7: Black | Highly-segregated metros	4.9 (4.9–4.9)	0.0 (0.0 to 0.0)*	4.9 (4.9–4.9)	0.0 (0.0 to 0.0)*	4.9 (4.9–4.9)	0.0 (0.0 to 0.0)	4.9 (4.9–5.0)	0.0 (0.0 to 0.0)	4.9 (4.9–5.0)	0.0 (0.0 to 0.0)*	0.0 (0.0 to 0.0)
America 8: White | Low-income Appalachia and Lower	5.0 (5.0–5.0)	0.0 (0.0 to 0.0)	5.0 (5.0–5.0)	0.0 (0.0 to 0.0)*	5.0 (5.0–5.0)	0.0 (0.0 to 0.0)	5.0 (5.0–5.0)	0.0 (0.0 to 0.0)	5.0 (5.0–5.0)	0.0 (0.0 to 0.0)*	0.0 (0.0 to 0.0)*

Mississippi Valley											
America 9: Black | Nonmetro and low-income South	4.9 (4.9–4.9)	0.0 (0.0 to 0.0)*	4.9 (4.9–4.9)	0.0 (0.0 to 0.0)	4.9 (4.9–5.0)	0.0 (0.0 to 0.0)	4.9 (4.9–5.0)	0.0 (0.0 to 0.0)	4.9 (4.9–5.0)	0.0 (0.0 to 0.0)*	0.0 (0.0 to 0.0)
America 10: AIAN | West	5.0 (5.0–5.0)	0.0 (0.0 to 0.0)	5.0 (4.9–5.0)	0.0 (0.0 to 0.0)	5.0 (4.9–5.0)	0.0 (0.0 to 0.0)	5.0 (4.9–5.0)	0.0 (0.0 to 0.0)	5.0 (4.9–5.0)	0.0 (0.0 to 0.0)	0.0 (0.0 to 0.0)

Numbers in parentheses are 95% uncertainty intervals.
*Indicates that the uncertainty bounds do not encompass 0.

Figure S3: Partial life expectancy, ages 5–24 years, males

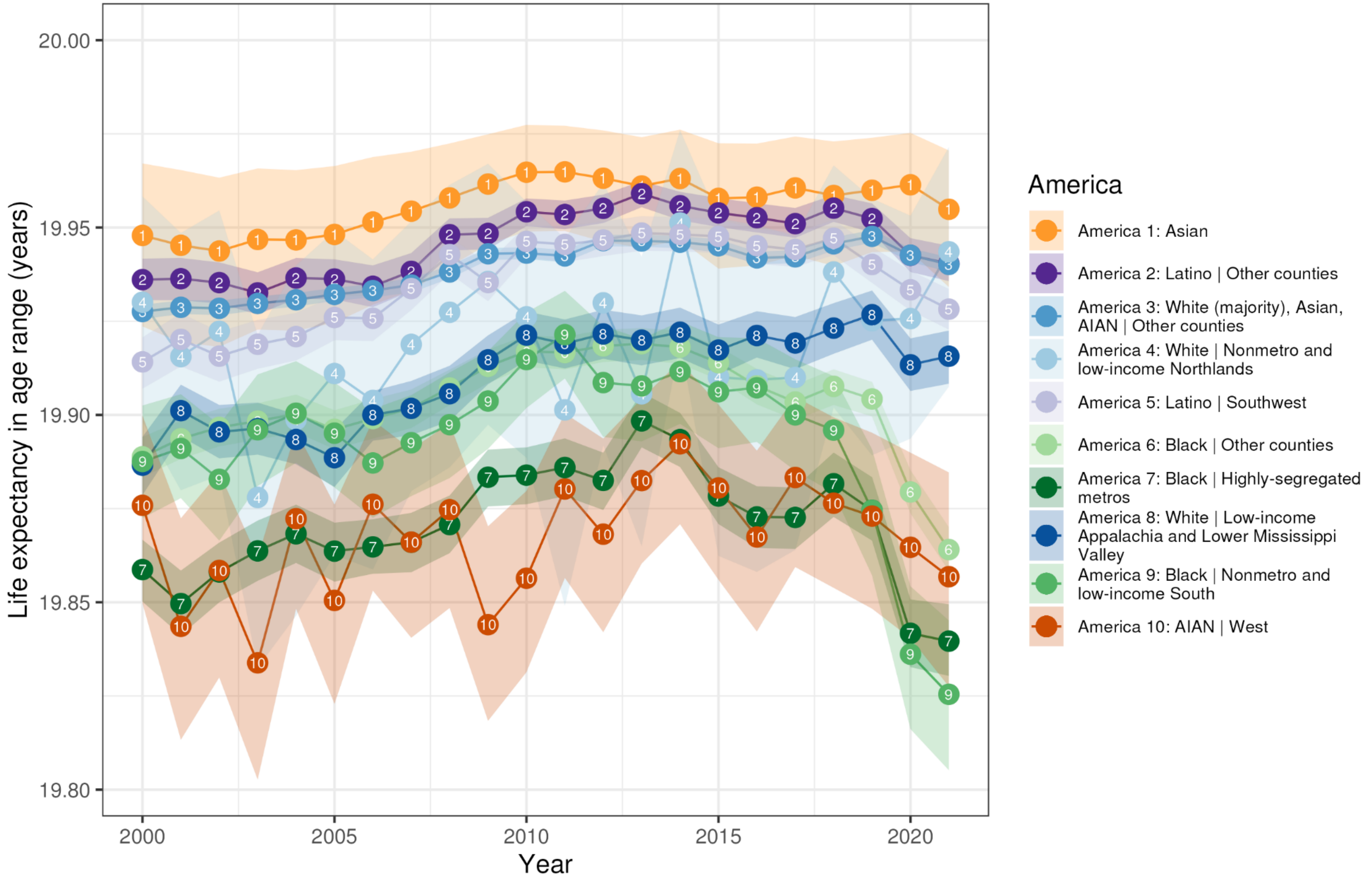

Table S3: Partial life expectancy, ages 5–24 years, males

America	Life expectancy, 2000	Change, 2000–10	Life expectancy, 2010	Change, 2010–19	Life expectancy, 2019	Change, 2019–20	Life expectancy, 2020	Change, 2020–21	Life expectancy, 2021	Change, 2000–19	Change, 2019–21
America 1: Asian	19.9 (19.9–20.0)	0.0 (0.0 to 0.0)*	20.0 (19.9–20.0)	0.0 (0.0 to 0.0)	20.0 (19.9–20.0)	0.0 (0.0 to 0.0)	20.0 (19.9–20.0)	0.0 (0.0 to 0.0)*	20.0 (19.9–20.0)	0.0 (0.0 to 0.0)*	0.0 (0.0 to 0.0)
America 2: Latino | Other counties	19.9 (19.9–19.9)	0.0 (0.0 to 0.0)*	20.0 (20.0–20.0)	0.0 (0.0 to 0.0)	20.0 (19.9–20.0)	0.0 (0.0 to 0.0)*	19.9 (19.9–19.9)	0.0 (0.0 to 0.0)	19.9 (19.9–19.9)	0.0 (0.0 to 0.0)*	0.0 (0.0 to 0.0)*
America 3: White (majority), Asian, AIAN | Other counties	19.9 (19.9–19.9)	0.0 (0.0 to 0.0)*	19.9 (19.9–19.9)	0.0 (0.0 to 0.0)*	19.9 (19.9–19.9)	0.0 (0.0 to 0.0)*	19.9 (19.9–19.9)	0.0 (0.0 to 0.0)*	19.9 (19.9–19.9)	0.0 (0.0 to 0.0)*	0.0 (0.0 to 0.0)*
America 4: White | Nonmetro and low-income Northlands	19.9 (19.9–20.0)	0.0 (−0.1 to 0.0)	19.9 (19.9–20.0)	0.0 (0.0 to 0.0)	19.9 (19.9–20.0)	0.0 (0.0 to 0.0)	19.9 (19.9–20.0)	0.0 (0.0 to 0.1)	19.9 (19.9–20.0)	0.0 (−0.1 to 0.0)	0.0 (0.0 to 0.1)
America 5: Latino | Southwest	19.9 (19.9–19.9)	0.0 (0.0 to 0.0)*	19.9 (19.9–20.0)	0.0 (0.0 to 0.0)*	19.9 (19.9–19.9)	0.0 (0.0 to 0.0)*	19.9 (19.9–19.9)	0.0 (0.0 to 0.0)	19.9 (19.9–19.9)	0.0 (0.0 to 0.0)*	0.0 (0.0 to 0.0)*
America 6: Black | Other counties	19.9 (19.9–19.9)	0.0 (0.0 to 0.0)*	19.9 (19.9–19.9)	0.0 (0.0 to 0.0)*	19.9 (19.9–19.9)	0.0 (0.0 to 0.0)*	19.9 (19.9–19.9)	0.0 (0.0 to 0.0)*	19.9 (19.9–19.9)	0.0 (0.0 to 0.0)*	0.0 (0.0 to 0.0)*
America 7: Black | Highly-segregated metros	19.9 (19.9–19.9)	0.0 (0.0 to 0.0)*	19.9 (19.9–19.9)	0.0 (0.0 to 0.0)	19.9 (19.9–19.9)	0.0 (0.0 to 0.0)*	19.8 (19.8–19.9)	0.0 (0.0 to 0.0)	19.8 (19.8–19.8)	0.0 (0.0 to 0.0)*	0.0 (0.0 to 0.0)*
America 8: White | Low-income Appalachia and Lower	19.9 (19.9–19.9)	0.0 (0.0 to 0.0)*	19.9 (19.9–19.9)	0.0 (0.0 to 0.0)	19.9 (19.9–19.9)	0.0 (0.0 to 0.0)*	19.9 (19.9–19.9)	0.0 (0.0 to 0.0)	19.9 (19.9–19.9)	0.0 (0.0 to 0.1)*	0.0 (0.0 to 0.0)*

Mississippi Valley											
America 9: Black | Nonmetro and low-income South	19.9 (19.9–19.9)	0.0 (0.0 to 0.0)*	19.9 (19.9–19.9)	0.0 (–0.1 to 0.0)*	19.9 (19.9–19.9)	0.0 (–0.1 to 0.0)*	19.8 (19.8–19.9)	0.0 (0.0 to 0.0)	19.8 (19.8–19.8)	0.0 (0.0 to 0.0)	0.0 (–0.1 to 0.0)*
America 10: AIAN | West	19.9 (19.8–19.9)	0.0 (0.0 to 0.0)	19.9 (19.8–19.9)	0.0 (0.0 to 0.0)	19.9 (19.8–19.9)	0.0 (0.0 to 0.0)	19.9 (19.8–19.9)	0.0 (0.0 to 0.0)	19.9 (19.8–19.9)	0.0 (0.0 to 0.0)	0.0 (0.0 to 0.0)

Numbers in parentheses are 95% uncertainty intervals.
*Indicates that the uncertainty bounds do not encompass 0.

Figure S4: Partial life expectancy, ages 5–24 years, females

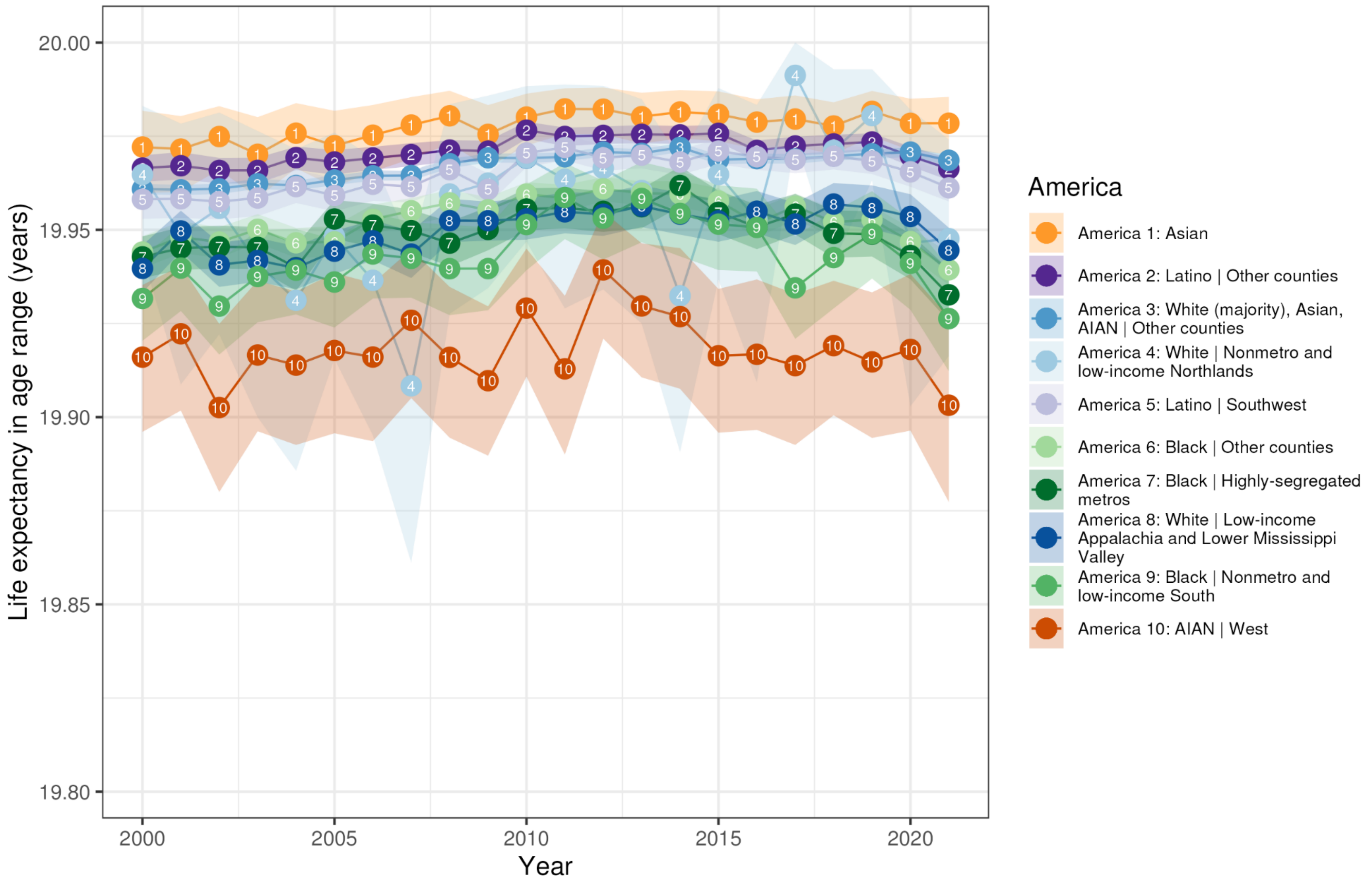

Table S4: Partial life expectancy, ages 5–24 years, females

America	Life expectancy, 2000	Change, 2000–10	Life expectancy, 2010	Change, 2010–19	Life expectancy, 2019	Change, 2019–20	Life expectancy, 2020	Change, 2020–21	Life expectancy, 2021	Change, 2000–19	Change, 2019–21
America 1: Asian	20.0 (20.0–20.0)	0.0 (0.0 to 0.0)*	20.0 (20.0–20.0)	0.0 (0.0 to 0.0)	20.0 (20.0–20.0)	0.0 (0.0 to 0.0)	20.0 (20.0–20.0)	0.0 (0.0 to 0.0)	20.0 (20.0–20.0)	0.0 (0.0 to 0.0)*	0.0 (0.0 to 0.0)
America 2: Latino | Other counties	20.0 (20.0–20.0)	0.0 (0.0 to 0.0)*	20.0 (20.0–20.0)	0.0 (0.0 to 0.0)*	20.0 (20.0–20.0)	0.0 (0.0 to 0.0)*	20.0 (20.0–20.0)	0.0 (0.0 to 0.0)*	20.0 (20.0–20.0)	0.0 (0.0 to 0.0)*	0.0 (0.0 to 0.0)*
America 3: White (majority), Asian, AIAN | Other counties	20.0 (20.0–20.0)	0.0 (0.0 to 0.0)*	20.0 (20.0–20.0)	0.0 (0.0 to 0.0)	20.0 (20.0–20.0)	0.0 (0.0 to 0.0)	20.0 (20.0–20.0)	0.0 (0.0 to 0.0)*	20.0 (20.0–20.0)	0.0 (0.0 to 0.0)*	0.0 (0.0 to 0.0)*
America 4: White | Nonmetro and low-income Northlands	20.0 (19.9–20.0)	0.0 (0.0 to 0.0)	20.0 (19.9–20.0)	0.0 (0.0 to 0.0)	20.0 (20.0–20.0)	0.0 (−0.1 to 0.0)	19.9 (19.9–20.0)	0.0 (0.0 to 0.1)	19.9 (19.9–20.0)	0.0 (0.0 to 0.0)	0.0 (−0.1 to 0.0)*
America 5: Latino | Southwest	20.0 (20.0–20.0)	0.0 (0.0 to 0.0)*	20.0 (20.0–20.0)	0.0 (0.0 to 0.0)	20.0 (20.0–20.0)	0.0 (0.0 to 0.0)	20.0 (20.0–20.0)	0.0 (0.0 to 0.0)	20.0 (20.0–20.0)	0.0 (0.0 to 0.0)*	0.0 (0.0 to 0.0)*
America 6: Black | Other counties	19.9 (19.9–19.9)	0.0 (0.0 to 0.0)*	20.0 (20.0–20.0)	0.0 (0.0 to 0.0)*	20.0 (19.9–20.0)	0.0 (0.0 to 0.0)*	19.9 (19.9–20.0)	0.0 (0.0 to 0.0)*	19.9 (19.9–19.9)	0.0 (0.0 to 0.0)*	0.0 (0.0 to 0.0)*
America 7: Black | Highly-segregated metros	19.9 (19.9–19.9)	0.0 (0.0 to 0.0)*	20.0 (19.9–20.0)	0.0 (0.0 to 0.0)	19.9 (19.9–20.0)	0.0 (0.0 to 0.0)	19.9 (19.9–19.9)	0.0 (0.0 to 0.0)*	19.9 (19.9–19.9)	0.0 (0.0 to 0.0)	0.0 (0.0 to 0.0)*
America 8: White | Low-income Appalachia and Lower	19.9 (19.9–19.9)	0.0 (0.0 to 0.0)*	20.0 (19.9–20.0)	0.0 (0.0 to 0.0)	20.0 (20.0–20.0)	0.0 (0.0 to 0.0)	20.0 (19.9–20.0)	0.0 (0.0 to 0.0)*	19.9 (19.9–20.0)	0.0 (0.0 to 0.0)*	0.0 (0.0 to 0.0)*

Mississippi Valley											
America 9: Black | Nonmetro and low-income South	19.9 (19.9–19.9)	0.0 (0.0 to 0.0)*	20.0 (19.9–20.0)	0.0 (0.0 to 0.0)	19.9 (19.9–20.0)	0.0 (0.0 to 0.0)	19.9 (19.9–20.0)	0.0 (0.0 to 0.0)	19.9 (19.9–19.9)	0.0 (0.0 to 0.0)*	0.0 (0.0 to 0.0)*
America 10: AIAN | West	19.9 (19.9–19.9)	0.0 (0.0 to 0.0)	19.9 (19.9–19.9)	0.0 (0.0 to 0.0)	19.9 (19.9–19.9)	0.0 (0.0 to 0.0)	19.9 (19.9–19.9)	0.0 (0.0 to 0.0)	19.9 (19.9–19.9)	0.0 (0.0 to 0.0)	0.0 (0.0 to 0.0)

Numbers in parentheses are 95% uncertainty intervals.
*Indicates that the uncertainty bounds do not encompass 0.

Figure S5: Partial life expectancy, ages 25–44 years, males

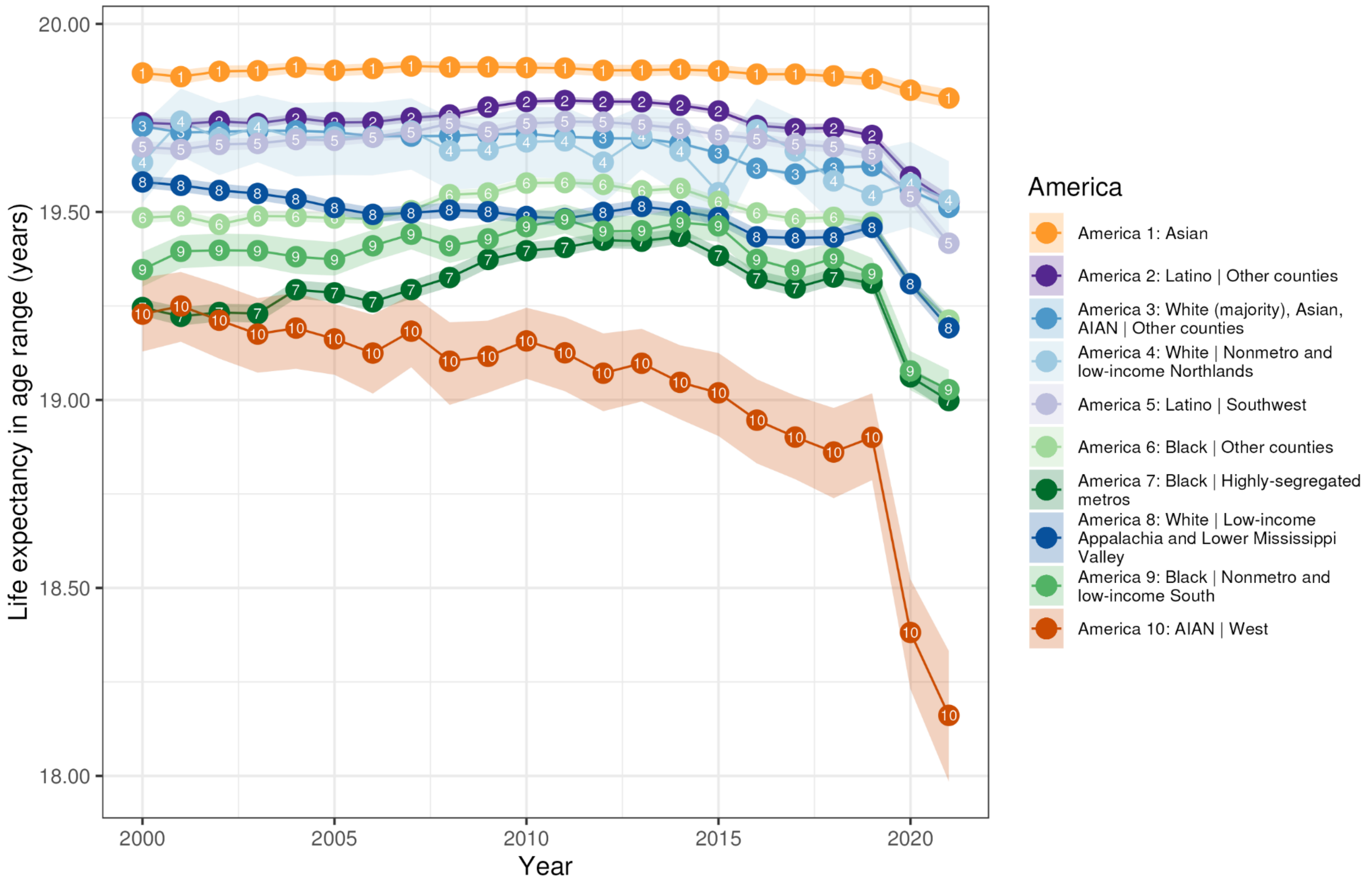

Table S5: Partial life expectancy, ages 25–44 years, males

America	Life expectancy, 2000	Change, 2000–10	Life expectancy, 2010	Change, 2010–19	Life expectancy, 2019	Change, 2019–20	Life expectancy, 2020	Change, 2020–21	Life expectancy, 2021	Change, 2000–19	Change, 2019–21
America 1: Asian	19.9 (19.9–19.9)	0.0 (0.0 to 0.0)*	19.9 (19.9–19.9)	0.0 (0.0 to 0.0)*	19.9 (19.8–19.9)	0.0 (0.0 to 0.0)*	19.8 (19.8–19.8)	0.0 (0.0 to 0.0)*	19.8 (19.8–19.8)	0.0 (0.0 to 0.0)*	−0.1 (−0.1 to 0.0)*
America 2: Latino | Other counties	19.7 (19.7–19.7)	0.1 (0.0 to 0.1)*	19.8 (19.8–19.8)	−0.1 (−0.1 to −0.1)*	19.7 (19.7–19.7)	−0.1 (−0.1 to −0.1)*	19.6 (19.6–19.6)	−0.1 (−0.1 to −0.1)*	19.5 (19.5–19.5)	0.0 (0.0 to 0.0)*	−0.2 (−0.2 to −0.2)*
America 3: White (majority), Asian, AIAN | Other counties	19.7 (19.7–19.7)	0.0 (0.0 to 0.0)*	19.7 (19.7–19.7)	−0.1 (−0.1 to −0.1)*	19.6 (19.6–19.6)	−0.1 (−0.1 to −0.1)*	19.6 (19.6–19.6)	0.0 (−0.1 to 0.0)*	19.5 (19.5–19.5)	−0.1 (−0.1 to −0.1)*	−0.1 (−0.1 to −0.1)*
America 4: White | Nonmetro and low-income Northlands	19.6 (19.5–19.7)	0.1 (−0.1 to 0.2)	19.7 (19.6–19.8)	−0.1 (−0.3 to 0.0)	19.5 (19.4–19.7)	0.0 (−0.1 to 0.2)	19.6 (19.5–19.7)	0.0 (−0.2 to 0.1)	19.5 (19.4–19.6)	−0.1 (−0.2 to 0.1)	0.0 (−0.2 to 0.1)
America 5: Latino | Southwest	19.7 (19.7–19.7)	0.1 (0.0 to 0.1)*	19.7 (19.7–19.7)	−0.1 (−0.1 to −0.1)*	19.7 (19.6–19.7)	−0.1 (−0.1 to −0.1)*	19.5 (19.5–19.6)	−0.1 (−0.1 to −0.1)*	19.4 (19.4–19.4)	0.0 (0.0 to 0.0)*	−0.2 (−0.3 to −0.2)*
America 6: Black | Other counties	19.5 (19.5–19.5)	0.1 (0.1 to 0.1)*	19.6 (19.6–19.6)	−0.1 (−0.1 to −0.1)*	19.5 (19.5–19.5)	−0.2 (−0.2 to −0.1)*	19.3 (19.3–19.3)	−0.1 (−0.1 to −0.1)*	19.2 (19.2–19.2)	0.0 (0.0 to 0.0)	−0.3 (−0.3 to −0.2)*
America 7: Black | Highly-segregated metros	19.2 (19.2–19.3)	0.2 (0.1 to 0.2)*	19.4 (19.4–19.4)	−0.1 (−0.1 to −0.1)*	19.3 (19.3–19.3)	−0.2 (−0.3 to −0.2)*	19.1 (19.0–19.1)	−0.1 (−0.1 to 0.0)*	19.0 (19.0–19.0)	0.1 (0.0 to 0.1)*	−0.3 (−0.3 to −0.3)*
America 8: White | Low-income Appalachia and Lower	19.6 (19.6–19.6)	−0.1 (−0.1 to −0.1)*	19.5 (19.5–19.5)	0.0 (−0.1 to 0.0)*	19.5 (19.4–19.5)	−0.2 (−0.2 to −0.1)*	19.3 (19.3–19.3)	−0.1 (−0.2 to −0.1)*	19.2 (19.2–19.2)	−0.1 (−0.1 to −0.1)*	−0.3 (−0.3 to −0.2)*

Mississippi Valley											
America 9: Black | Nonmetro and low-income South	19.3 (19.3–19.4)	0.1 (0.1 to 0.2)*	19.5 (19.4–19.5)	–0.1 (–0.2 to –0.1)*	19.3 (19.3–19.4)	–0.3 (–0.3 to –0.2)*	19.1 (19.0–19.1)	0.0 (–0.1 to 0.0)	19.0 (19.0–19.1)	0.0 (–0.1 to 0.1)	–0.3 (–0.4 to –0.2)*
America 10: AIAN | West	19.2 (19.1–19.3)	–0.1 (–0.2 to 0.0)	19.2 (19.1–19.2)	–0.3 (–0.4 to –0.2)*	18.9 (18.8–19.0)	–0.5 (–0.6 to –0.4)*	18.4 (18.2–18.5)	–0.2 (–0.3 to –0.1)*	18.2 (18.0–18.3)	–0.3 (–0.4 to –0.2)*	–0.7 (–0.9 to –0.6)*

Numbers in parentheses are 95% uncertainty intervals.
*Indicates that the uncertainty bounds do not encompass 0.

Figure S6: Partial life expectancy, ages 25–44 years, females

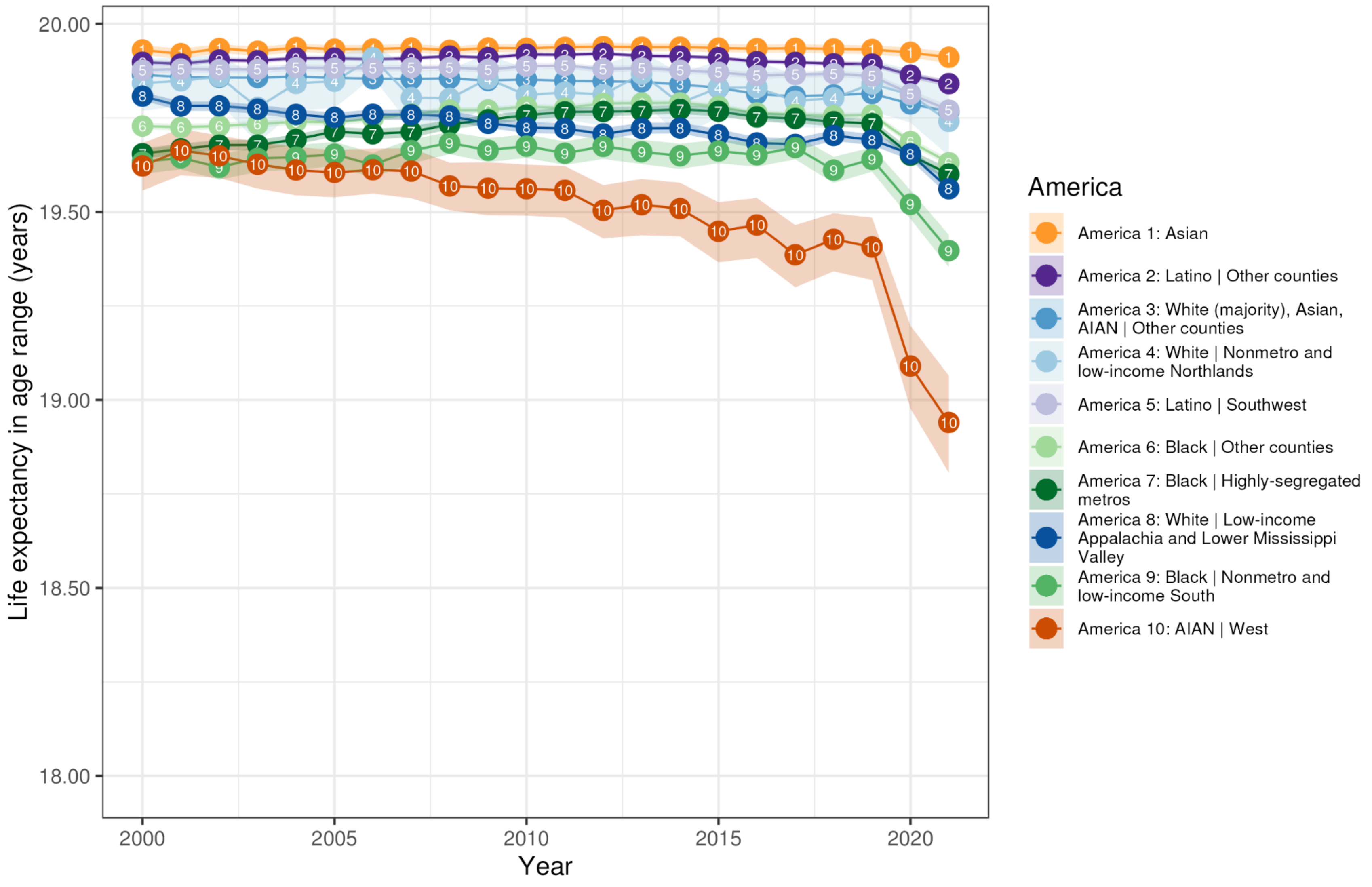

Table S6: Partial life expectancy, ages 25–44 years, females

America	Life expectancy, 2000	Change, 2000–10	Life expectancy, 2010	Change, 2010–19	Life expectancy, 2019	Change, 2019–20	Life expectancy, 2020	Change, 2020–21	Life expectancy, 2021	Change, 2000–19	Change, 2019–21
America 1: Asian	19.9 (19.9–19.9)	0.0 (0.0 to 0.0)	19.9 (19.9–19.9)	0.0 (0.0 to 0.0)	19.9 (19.9–19.9)	0.0 (0.0 to 0.0)*	19.9 (19.9–19.9)	0.0 (0.0 to 0.0)*	19.9 (19.9–19.9)	0.0 (0.0 to 0.0)	0.0 (0.0 to 0.0)*
America 2: Latino | Other counties	19.9 (19.9–19.9)	0.0 (0.0 to 0.0)*	19.9 (19.9–19.9)	0.0 (0.0 to 0.0)*	19.9 (19.9–19.9)	0.0 (0.0 to 0.0)*	19.9 (19.9–19.9)	0.0 (0.0 to 0.0)*	19.8 (19.8–19.8)	0.0 (0.0 to 0.0)	–0.1 (–0.1 to 0.0)*
America 3: White (majority), Asian, AIAN | Other counties	19.9 (19.9–19.9)	0.0 (0.0 to 0.0)*	19.9 (19.8–19.9)	0.0 (0.0 to 0.0)*	19.8 (19.8–19.8)	0.0 (0.0 to 0.0)*	19.8 (19.8–19.8)	0.0 (0.0 to 0.0)*	19.8 (19.8–19.8)	–0.1 (–0.1 to 0.0)*	0.0 (–0.1 to 0.0)*
America 4: White | Nonmetro and low-income Northlands	19.8 (19.8–19.9)	0.0 (–0.1 to 0.1)	19.8 (19.7–19.9)	0.0 (–0.1 to 0.1)	19.8 (19.8–19.9)	0.0 (–0.1 to 0.1)	19.8 (19.7–19.9)	–0.1 (–0.2 to 0.0)	19.7 (19.6–19.8)	0.0 (–0.1 to 0.1)	–0.1 (–0.2 to 0.0)
America 5: Latino | Southwest	19.9 (19.9–19.9)	0.0 (0.0 to 0.0)	19.9 (19.9–19.9)	0.0 (0.0 to 0.0)*	19.9 (19.9–19.9)	0.0 (–0.1 to 0.0)*	19.8 (19.8–19.8)	0.0 (–0.1 to 0.0)*	19.8 (19.8–19.8)	0.0 (0.0 to 0.0)*	–0.1 (–0.1 to –0.1)*
America 6: Black | Other counties	19.7 (19.7–19.7)	0.1 (0.0 to 0.1)*	19.8 (19.8–19.8)	0.0 (0.0 to 0.0)*	19.8 (19.8–19.8)	–0.1 (–0.1 to –0.1)*	19.7 (19.7–19.7)	–0.1 (–0.1 to 0.0)*	19.6 (19.6–19.6)	0.0 (0.0 to 0.0)*	–0.1 (–0.1 to –0.1)*
America 7: Black | Highly-segregated metros	19.7 (19.6–19.7)	0.1 (0.1 to 0.1)*	19.8 (19.7–19.8)	0.0 (0.0 to 0.0)*	19.7 (19.7–19.7)	–0.1 (–0.1 to –0.1)*	19.7 (19.6–19.7)	–0.1 (–0.1 to 0.0)*	19.6 (19.6–19.6)	0.1 (0.1 to 0.1)*	–0.1 (–0.2 to –0.1)*
America 8: White | Low-income Appalachia and Lower	19.8 (19.8–19.8)	–0.1 (–0.1 to –0.1)*	19.7 (19.7–19.7)	0.0 (–0.1 to 0.0)*	19.7 (19.7–19.7)	0.0 (–0.1 to 0.0)*	19.7 (19.6–19.7)	–0.1 (–0.1 to –0.1)*	19.6 (19.5–19.6)	–0.1 (–0.1 to –0.1)*	–0.1 (–0.2 to –0.1)*

Mississippi Valley											
America 9: Black | Nonmetro and low-income South	19.6 (19.6–19.7)	0.0 (0.0 to 0.1)	19.7 (19.6–19.7)	0.0 (–0.1 to 0.0)	19.6 (19.6–19.7)	–0.1 (–0.2 to –0.1)*	19.5 (19.5–19.6)	–0.1 (–0.2 to –0.1)*	19.4 (19.4–19.4)	0.0 (0.0 to 0.1)	–0.2 (–0.3 to –0.2)*
America 10: AIAN | West	19.6 (19.6–19.7)	–0.1 (–0.1 to 0.0)	19.6 (19.5–19.6)	–0.2 (–0.2 to –0.1)*	19.4 (19.3–19.5)	–0.3 (–0.4 to –0.2)*	19.1 (19.0–19.2)	–0.1 (–0.2 to –0.1)*	18.9 (18.8–19.1)	–0.2 (–0.3 to –0.1)*	–0.5 (–0.6 to –0.4)*

Numbers in parentheses are 95% uncertainty intervals.
*Indicates that the uncertainty bounds do not encompass 0.

Figure S7: Partial life expectancy, ages 45–64 years, males

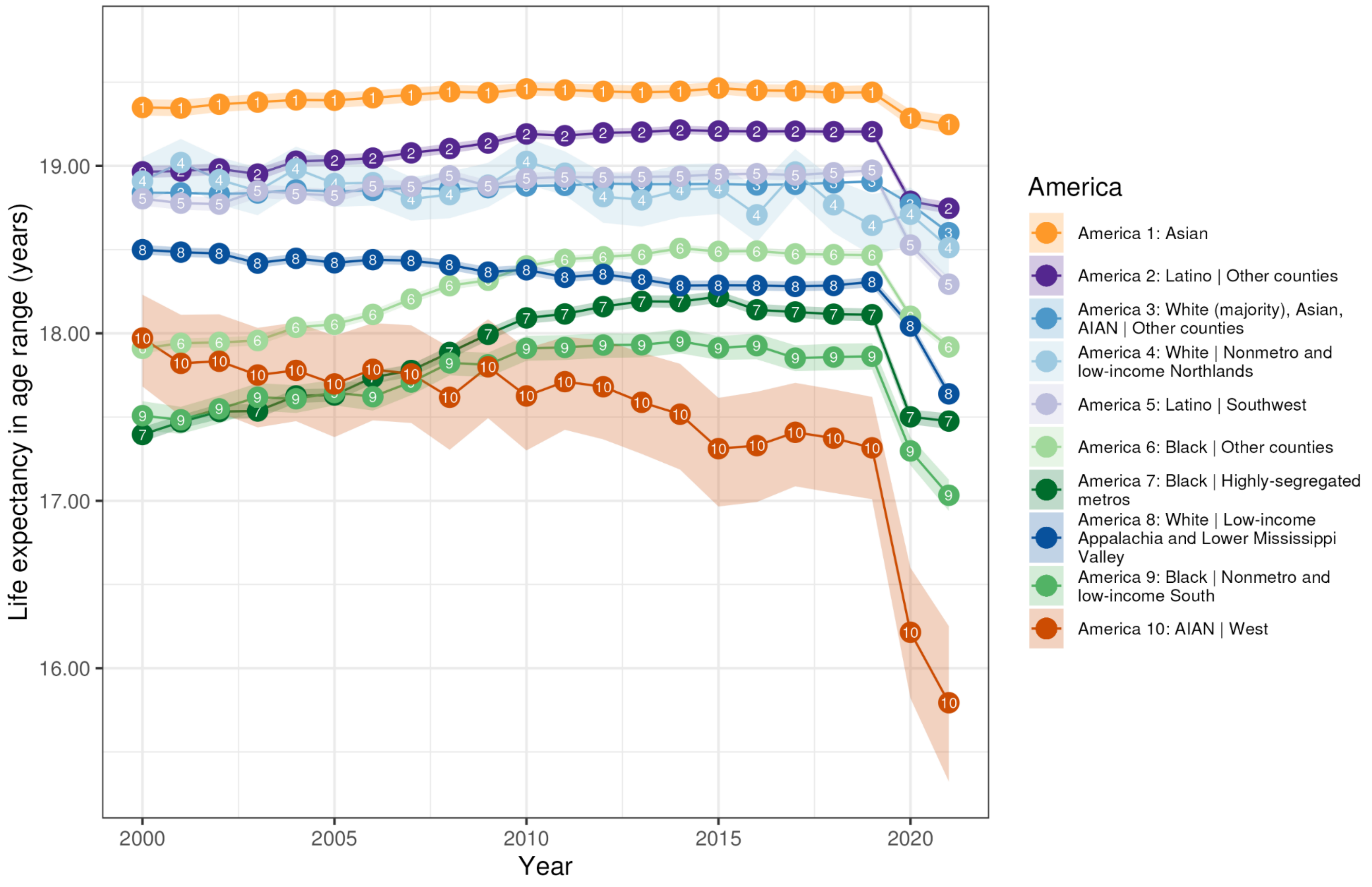

Table S7: Partial life expectancy, ages 45–64 years, males

America	Life expectancy, 2000	Change, 2000–10	Life expectancy, 2010	Change, 2010–19	Life expectancy, 2019	Change, 2019–20	Life expectancy, 2020	Change, 2020–21	Life expectancy, 2021	Change, 2000–19	Change, 2019–21
America 1: Asian	19.3 (19.3–19.4)	0.1 (0.1 to 0.1)*	19.5 (19.4–19.5)	0.0 (0.0 to 0.0)*	19.4 (19.4–19.5)	−0.2 (−0.2 to −0.1)*	19.3 (19.2–19.3)	0.0 (−0.1 to 0.0)*	19.2 (19.2–19.3)	0.1 (0.1 to 0.1)*	−0.2 (−0.2 to −0.2)*
America 2: Latino | Other counties	19.0 (18.9–19.0)	0.2 (0.2 to 0.3)*	19.2 (19.2–19.2)	0.0 (0.0 to 0.0)	19.2 (19.2–19.2)	−0.4 (−0.4 to −0.4)*	18.8 (18.8–18.8)	0.0 (−0.1 to 0.0)*	18.7 (18.7–18.8)	0.2 (0.2 to 0.3)*	−0.5 (−0.5 to −0.4)*
America 3: White (majority), Asian, AIAN | Other counties	18.8 (18.8–18.8)	0.0 (0.0 to 0.0)*	18.9 (18.9–18.9)	0.0 (0.0 to 0.0)*	18.9 (18.9–18.9)	−0.1 (−0.1 to −0.1)*	18.8 (18.8–18.8)	−0.2 (−0.2 to −0.2)*	18.6 (18.6–18.6)	0.1 (0.1 to 0.1)*	−0.3 (−0.3 to −0.3)*
America 4: White | Nonmetro and low-income Northlands	18.9 (18.8–19.1)	0.1 (−0.1 to 0.3)	19.0 (18.9–19.2)	−0.4 (−0.6 to −0.1)*	18.6 (18.5–18.8)	0.1 (−0.2 to 0.3)	18.7 (18.5–18.9)	−0.2 (−0.4 to 0.1)	18.5 (18.3–18.7)	−0.3 (−0.5 to 0.0)*	−0.1 (−0.4 to 0.1)
America 5: Latino | Southwest	18.8 (18.8–18.8)	0.1 (0.1 to 0.2)*	18.9 (18.9–19.0)	0.0 (0.0 to 0.1)*	19.0 (18.9–19.0)	−0.4 (−0.5 to −0.4)*	18.5 (18.5–18.6)	−0.2 (−0.3 to −0.2)*	18.3 (18.3–18.3)	0.2 (0.1 to 0.2)*	−0.7 (−0.7 to −0.6)*
America 6: Black | Other counties	17.9 (17.9–17.9)	0.5 (0.5 to 0.5)*	18.4 (18.4–18.4)	0.1 (0.0 to 0.1)*	18.5 (18.4–18.5)	−0.4 (−0.4 to −0.3)*	18.1 (18.1–18.1)	−0.2 (−0.2 to −0.2)*	17.9 (17.9–17.9)	0.6 (0.5 to 0.6)*	−0.5 (−0.6 to −0.5)*
America 7: Black | Highly-segregated metros	17.4 (17.4–17.4)	0.7 (0.6 to 0.8)*	18.1 (18.1–18.1)	0.0 (0.0 to 0.1)	18.1 (18.1–18.1)	−0.6 (−0.7 to −0.6)*	17.5 (17.5–17.5)	0.0 (−0.1 to 0.0)	17.5 (17.4–17.5)	0.7 (0.7 to 0.8)*	−0.6 (−0.7 to −0.6)*
America 8: White | Low-income Appalachia and Lower	18.5 (18.5–18.5)	−0.1 (−0.2 to −0.1)*	18.4 (18.3–18.4)	−0.1 (−0.1 to 0.0)*	18.3 (18.3–18.3)	−0.3 (−0.3 to −0.2)*	18.0 (18.0–18.1)	−0.4 (−0.5 to −0.4)*	17.6 (17.6–17.7)	−0.2 (−0.2 to −0.2)*	−0.7 (−0.7 to −0.6)*

Mississippi Valley											
America 9: Black | Nonmetro and low-income South	17.5 (17.4–17.6)	0.4 (0.3 to 0.5)*	17.9 (17.8–18.0)	0.0 (−0.2 to 0.1)	17.9 (17.8–17.9)	−0.6 (−0.7 to −0.4)*	17.3 (17.2–17.4)	−0.3 (−0.4 to −0.1)*	17.0 (16.9–17.1)	0.4 (0.2 to 0.5)*	−0.8 (−1.0 to −0.7)*
America 10: AIAN | West	18.0 (17.7–18.2)	−0.3 (−0.5 to −0.2)*	17.6 (17.3–17.9)	−0.3 (−0.5 to −0.2)*	17.3 (17.0–17.6)	−1.1 (−1.3 to −0.9)*	16.2 (15.8–16.6)	−0.4 (−0.6 to −0.2)*	15.8 (15.3–16.3)	−0.7 (−0.8 to −0.5)*	−1.5 (−1.7 to −1.3)*

Numbers in parentheses are 95% uncertainty intervals.
*Indicates that the uncertainty bounds do not encompass 0.

Figure S8: Partial life expectancy, ages 45–64 years, females

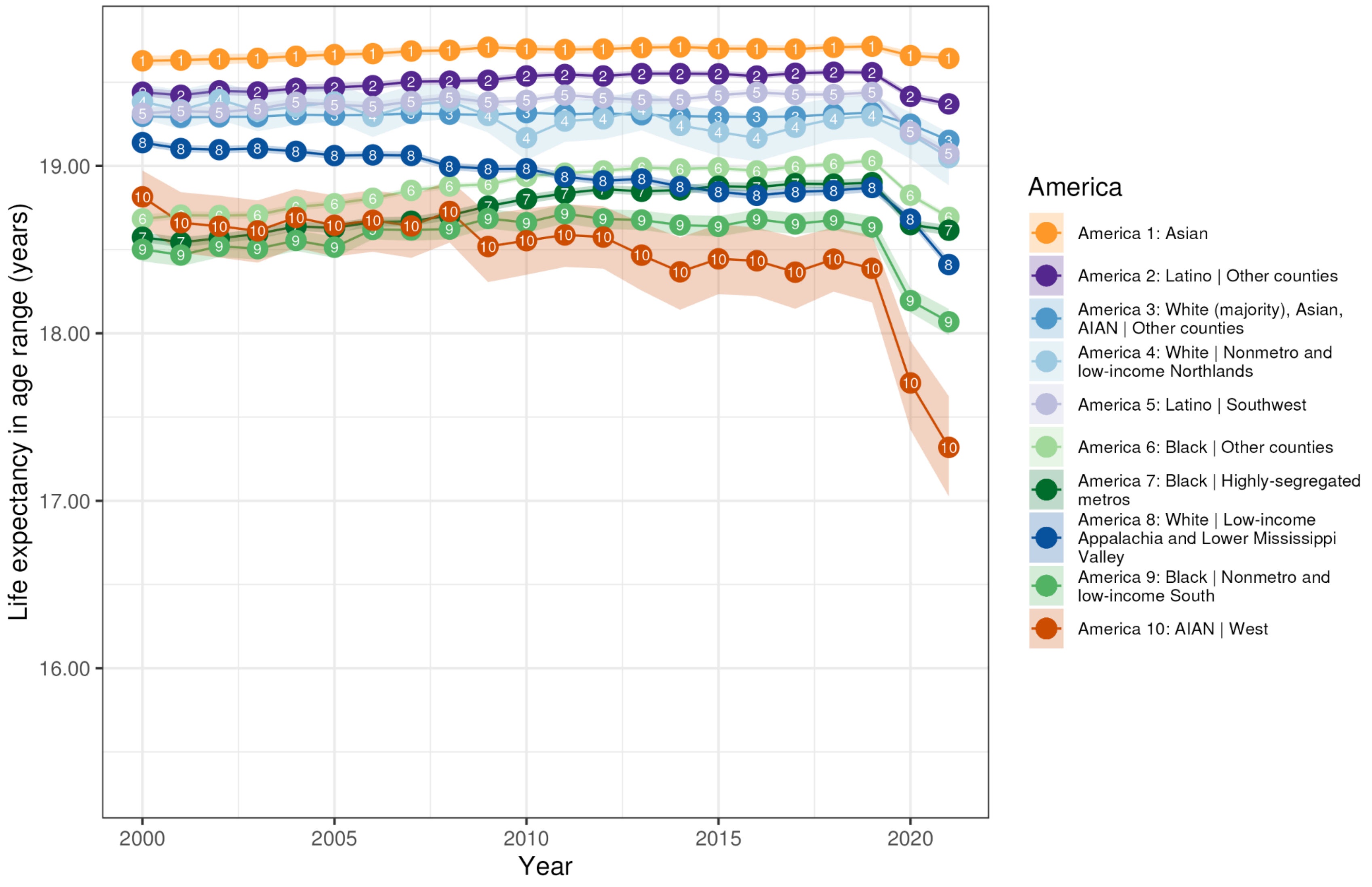

Table S8: Partial life expectancy, ages 45–64 years, females

America	Life expectancy, 2000	Change, 2000–10	Life expectancy, 2010	Change, 2010–19	Life expectancy, 2019	Change, 2019–20	Life expectancy, 2020	Change, 2020–21	Life expectancy, 2021	Change, 2000–19	Change, 2019–21
America 1: Asian	19.6 (19.6–19.7)	0.1 (0.1 to 0.1)*	19.7 (19.7–19.7)	0.0 (0.0 to 0.0)*	19.7 (19.7–19.7)	−0.1 (−0.1 to 0.0)*	19.7 (19.6–19.7)	0.0 (0.0 to 0.0)*	19.6 (19.6–19.7)	0.1 (0.1 to 0.1)*	−0.1 (−0.1 to −0.1)*
America 2: Latino | Other counties	19.4 (19.4–19.5)	0.1 (0.1 to 0.1)*	19.5 (19.5–19.6)	0.0 (0.0 to 0.0)*	19.6 (19.5–19.6)	−0.1 (−0.2 to −0.1)*	19.4 (19.4–19.4)	0.0 (−0.1 to 0.0)*	19.4 (19.3–19.4)	0.1 (0.1 to 0.1)*	−0.2 (−0.2 to −0.2)*
America 3: White (majority), Asian, AIAN | Other counties	19.3 (19.3–19.3)	0.0 (0.0 to 0.0)*	19.3 (19.3–19.3)	0.0 (0.0 to 0.0)	19.3 (19.3–19.3)	−0.1 (−0.1 to −0.1)*	19.2 (19.2–19.3)	−0.1 (−0.1 to −0.1)*	19.2 (19.1–19.2)	0.0 (0.0 to 0.0)*	−0.2 (−0.2 to −0.2)*
America 4: White | Nonmetro and low-income Northlands	19.4 (19.3–19.5)	−0.2 (−0.4 to −0.1)*	19.2 (19.0–19.3)	0.1 (0.0 to 0.3)	19.3 (19.2–19.4)	−0.1 (−0.3 to 0.1)	19.2 (19.0–19.3)	−0.1 (−0.4 to 0.1)	19.0 (18.9–19.2)	−0.1 (−0.3 to 0.1)	−0.2 (−0.5 to 0.0)*
America 5: Latino | Southwest	19.3 (19.3–19.3)	0.1 (0.0 to 0.1)*	19.4 (19.4–19.4)	0.0 (0.0 to 0.1)*	19.4 (19.4–19.5)	−0.2 (−0.3 to −0.2)*	19.2 (19.2–19.2)	−0.1 (−0.2 to −0.1)*	19.1 (19.0–19.1)	0.1 (0.1 to 0.2)*	−0.4 (−0.4 to −0.3)*
America 6: Black | Other counties	18.7 (18.7–18.7)	0.3 (0.2 to 0.3)*	18.9 (18.9–19.0)	0.1 (0.1 to 0.1)*	19.0 (19.0–19.0)	−0.2 (−0.2 to −0.2)*	18.8 (18.8–18.8)	−0.1 (−0.2 to −0.1)*	18.7 (18.7–18.7)	0.3 (0.3 to 0.4)*	−0.3 (−0.4 to −0.3)*
America 7: Black | Highly-segregated metros	18.6 (18.5–18.6)	0.2 (0.2 to 0.3)*	18.8 (18.8–18.8)	0.1 (0.1 to 0.1)*	18.9 (18.9–18.9)	−0.3 (−0.3 to −0.2)*	18.6 (18.6–18.7)	0.0 (−0.1 to 0.0)	18.6 (18.6–18.6)	0.3 (0.3 to 0.4)*	−0.3 (−0.3 to −0.2)*
America 8: White | Low-income Appalachia and Lower	19.1 (19.1–19.2)	−0.2 (−0.2 to −0.1)*	19.0 (19.0–19.0)	−0.1 (−0.1 to −0.1)*	18.9 (18.8–18.9)	−0.2 (−0.2 to −0.1)*	18.7 (18.7–18.7)	−0.3 (−0.3 to −0.2)*	18.4 (18.4–18.4)	−0.3 (−0.3 to −0.2)*	−0.5 (−0.5 to −0.4)*

Mississippi Valley											
America 9: Black | Nonmetro and low-income South	18.5 (18.4–18.6)	0.2 (0.1 to 0.3)*	18.7 (18.6–18.7)	0.0 (–0.1 to 0.1)	18.6 (18.6–18.7)	–0.4 (–0.5 to –0.3)*	18.2 (18.1–18.3)	–0.1 (–0.2 to 0.0)*	18.1 (18.0–18.1)	0.1 (0.0 to 0.2)*	–0.6 (–0.7 to –0.5)*
America 10: AIAN | West	18.8 (18.6–19.0)	–0.3 (–0.4 to –0.1)*	18.6 (18.4–18.7)	–0.2 (–0.3 to 0.0)*	18.4 (18.2–18.6)	–0.7 (–0.8 to –0.5)*	17.7 (17.4–18.0)	–0.4 (–0.6 to –0.2)*	17.3 (17.0–17.6)	–0.4 (–0.6 to –0.3)*	–1.1 (–1.2 to –0.9)*

Numbers in parentheses are 95% uncertainty intervals.
*Indicates that the uncertainty bounds do not encompass 0.

Figure S9: Partial life expectancy, ages 65–84 years, males

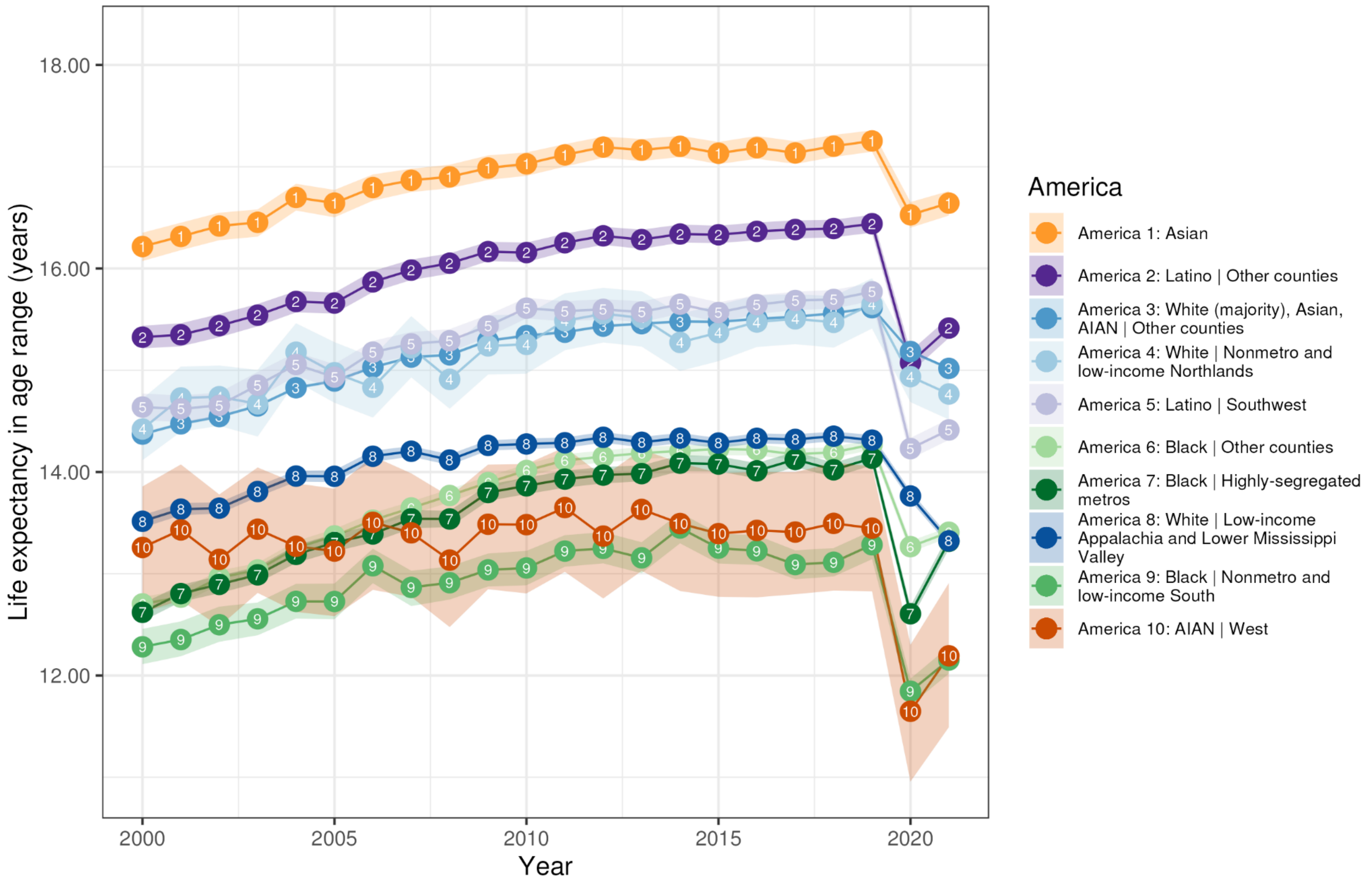

Table S9: Partial life expectancy, ages 65–84 years, males

America	Life expectancy, 2000	Change, 2000–10	Life expectancy, 2010	Change, 2010–19	Life expectancy, 2019	Change, 2019–20	Life expectancy, 2020	Change, 2020–21	Life expectancy, 2021	Change, 2000–19	Change, 2019–21
America 1: Asian	16.2 (16.1–16.4)	0.8 (0.7 to 0.9)*	17.0 (16.9–17.1)	0.2 (0.2 to 0.3)*	17.3 (17.1–17.4)	−0.7 (−0.8 to −0.7)*	16.5 (16.4–16.7)	0.1 (0.1 to 0.2)*	16.6 (16.5–16.8)	1.0 (0.9 to 1.1)*	−0.6 (−0.7 to −0.6)*
America 2: Latino | Other counties	15.3 (15.2–15.4)	0.8 (0.7 to 0.9)*	16.2 (16.1–16.3)	0.3 (0.2 to 0.3)*	16.4 (16.4–16.5)	−1.4 (−1.4 to −1.3)*	15.1 (15.0–15.2)	0.3 (0.3 to 0.4)*	15.4 (15.3–15.5)	1.1 (1.0 to 1.2)*	−1.0 (−1.1 to −1.0)*
America 3: White (majority), Asian, AIAN | Other counties	14.4 (14.4–14.4)	1.0 (0.9 to 1.0)*	15.3 (15.3–15.4)	0.3 (0.3 to 0.3)*	15.6 (15.6–15.6)	−0.4 (−0.4 to −0.4)*	15.2 (15.2–15.2)	−0.2 (−0.2 to −0.2)*	15.0 (15.0–15.0)	1.2 (1.2 to 1.3)*	−0.6 (−0.6 to −0.6)*
America 4: White | Nonmetro and low-income Northlands	14.4 (14.1–14.7)	0.8 (0.4 to 1.2)*	15.3 (15.0–15.5)	0.4 (0.0 to 0.8)*	15.6 (15.4–15.9)	−0.7 (−1.0 to −0.4)*	14.9 (14.7–15.2)	−0.2 (−0.5 to 0.2)	14.8 (14.5–15.0)	1.2 (0.9 to 1.6)*	−0.9 (−1.2 to −0.5)*
America 5: Latino | Southwest	14.6 (14.5–14.8)	1.0 (0.8 to 1.1)*	15.6 (15.5–15.7)	0.2 (0.1 to 0.3)*	15.8 (15.7–15.9)	−1.5 (−1.6 to −1.5)*	14.2 (14.1–14.3)	0.2 (0.1 to 0.3)*	14.4 (14.3–14.5)	1.1 (1.0 to 1.3)*	−1.4 (−1.4 to −1.3)*
America 6: Black | Other counties	12.7 (12.6–12.8)	1.3 (1.2 to 1.4)*	14.0 (14.0–14.1)	0.3 (0.2 to 0.3)*	14.3 (14.2–14.3)	−1.0 (−1.1 to −0.9)*	13.3 (13.2–13.3)	0.1 (0.1 to 0.2)*	13.4 (13.4–13.5)	1.6 (1.5 to 1.6)*	−0.9 (−0.9 to −0.8)*
America 7: Black | Highly-segregated metros	12.6 (12.5–12.7)	1.2 (1.1 to 1.4)*	13.9 (13.8–13.9)	0.3 (0.2 to 0.4)*	14.1 (14.1–14.2)	−1.5 (−1.6 to −1.4)*	12.6 (12.5–12.7)	0.7 (0.6 to 0.8)*	13.3 (13.2–13.4)	1.5 (1.4 to 1.6)*	−0.8 (−0.9 to −0.7)*
America 8: White | Low-income Appalachia and Lower	13.5 (13.5–13.6)	0.8 (0.7 to 0.8)*	14.3 (14.2–14.3)	0.0 (0.0 to 0.1)	14.3 (14.3–14.4)	−0.5 (−0.6 to −0.5)*	13.8 (13.7–13.8)	−0.4 (−0.5 to −0.4)*	13.3 (13.3–13.4)	0.8 (0.7 to 0.9)*	−1.0 (−1.1 to −0.9)*

Mississippi Valley											
America 9: Black | Nonmetro and low-income South	12.3 (12.1–12.5)	0.8 (0.5 to 1.0)*	13.1 (12.9–13.2)	0.2 (0.0 to 0.4)*	13.3 (13.1–13.4)	−1.4 (−1.6 to −1.3)*	11.8 (11.7–12.0)	0.3 (0.1 to 0.5)*	12.2 (12.0–12.3)	1.0 (0.8 to 1.2)*	−1.1 (−1.3 to −0.9)*
America 10: AIAN | West	13.3 (12.6–13.9)	0.2 (−0.1 to 0.6)	13.5 (12.8–14.1)	0.0 (−0.3 to 0.3)	13.4 (12.8–14.1)	−1.8 (−2.1 to −1.5)*	11.6 (11.0–12.3)	0.5 (0.3 to 0.8)*	12.2 (11.5–12.9)	0.2 (−0.2 to 0.5)	−1.3 (−1.5 to −1.0)*

Numbers in parentheses are 95% uncertainty intervals.
*Indicates that the uncertainty bounds do not encompass 0.

Figure S10: Partial life expectancy, ages 65–84 years, females

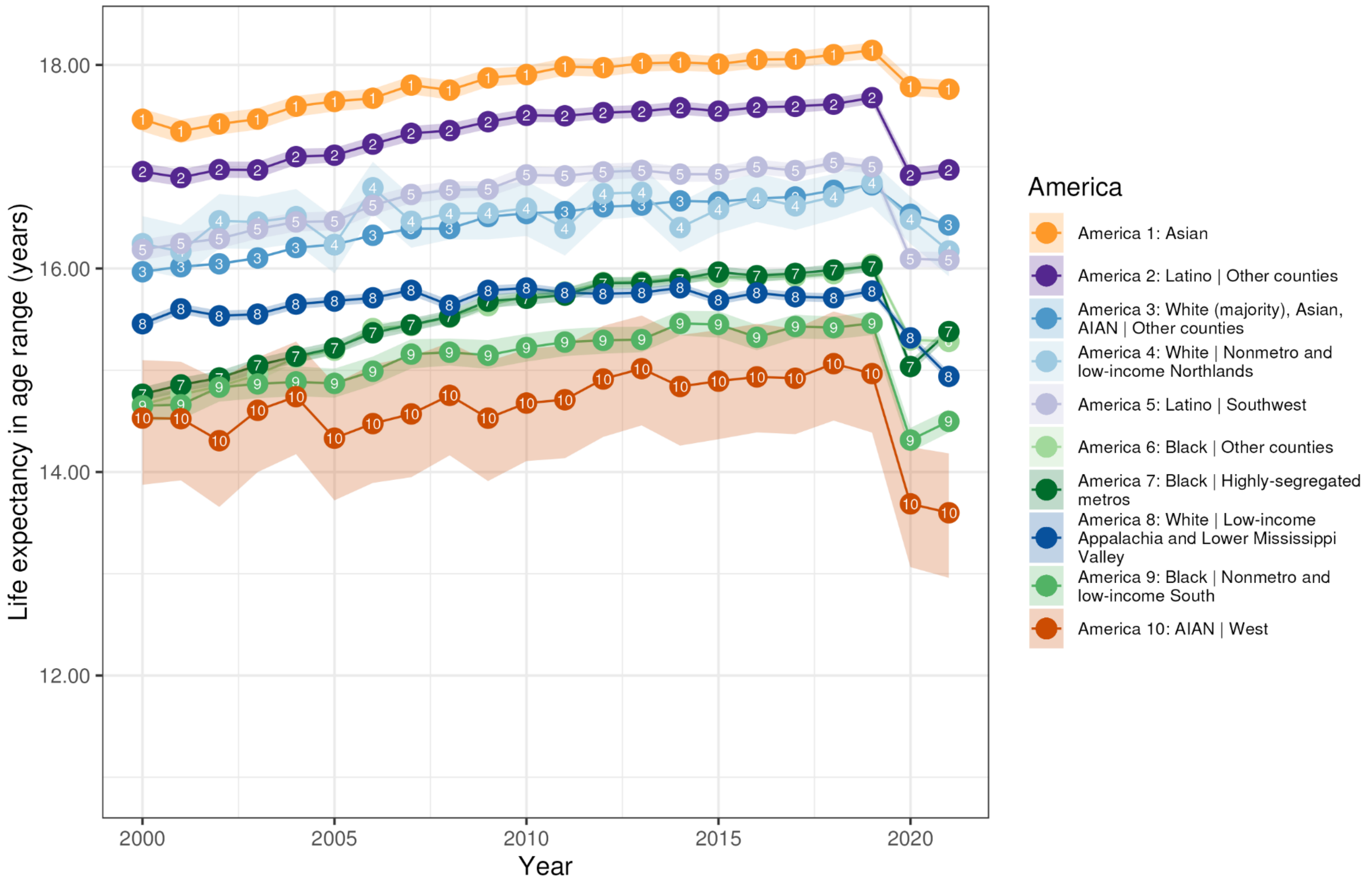

Table S10: Partial life expectancy, ages 65–84 years, females

America	Life expectancy, 2000	Change, 2000–10	Life expectancy, 2010	Change, 2010–19	Life expectancy, 2019	Change, 2019–20	Life expectancy, 2020	Change, 2020–21	Life expectancy, 2021	Change, 2000–19	Change, 2019–21
America 1: Asian	17.5 (17.3–17.6)	0.4 (0.4 to 0.5)*	17.9 (17.8–18.0)	0.2 (0.2 to 0.3)*	18.1 (18.1–18.2)	−0.4 (−0.4 to −0.3)*	17.8 (17.7–17.9)	0.0 (−0.1 to 0.0)	17.8 (17.7–17.9)	0.7 (0.6 to 0.7)*	−0.4 (−0.4 to −0.3)*
America 2: Latino | Other counties	17.0 (16.9–17.0)	0.6 (0.5 to 0.6)*	17.5 (17.4–17.6)	0.2 (0.1 to 0.2)*	17.7 (17.6–17.7)	−0.8 (−0.8 to −0.7)*	16.9 (16.8–17.0)	0.1 (0.0 to 0.1)*	17.0 (16.9–17.0)	0.7 (0.7 to 0.8)*	−0.7 (−0.8 to −0.7)*
America 3: White (majority), Asian, AIAN | Other counties	16.0 (16.0–16.0)	0.6 (0.6 to 0.6)*	16.5 (16.5–16.6)	0.3 (0.3 to 0.3)*	16.8 (16.8–16.8)	−0.3 (−0.3 to −0.3)*	16.5 (16.5–16.5)	−0.1 (−0.1 to −0.1)*	16.4 (16.4–16.4)	0.9 (0.8 to 0.9)*	−0.4 (−0.4 to −0.4)*
America 4: White | Nonmetro and low-income Northlands	16.2 (16.0–16.5)	0.4 (0.0 to 0.7)	16.6 (16.3–16.9)	0.2 (−0.1 to 0.6)	16.8 (16.6–17.1)	−0.4 (−0.7 to 0.0)*	16.5 (16.2–16.7)	−0.3 (−0.6 to 0.0)	16.2 (15.9–16.4)	0.6 (0.3 to 0.9)*	−0.7 (−1.0 to −0.3)*
America 5: Latino | Southwest	16.2 (16.1–16.3)	0.7 (0.6 to 0.8)*	16.9 (16.8–17.0)	0.1 (0.0 to 0.2)*	17.0 (16.9–17.1)	−0.9 (−1.0 to −0.8)*	16.1 (16.0–16.2)	0.0 (−0.1 to 0.1)	16.1 (16.0–16.2)	0.8 (0.7 to 0.9)*	−0.9 (−1.0 to −0.8)*
America 6: Black | Other counties	14.7 (14.6–14.7)	1.1 (1.0 to 1.1)*	15.7 (15.7–15.8)	0.3 (0.3 to 0.4)*	16.0 (16.0–16.1)	−0.7 (−0.8 to −0.7)*	15.3 (15.3–15.3)	0.0 (−0.1 to 0.0)	15.3 (15.2–15.3)	1.4 (1.3 to 1.4)*	−0.8 (−0.8 to −0.7)*
America 7: Black | Highly-segregated metros	14.8 (14.7–14.8)	0.9 (0.9 to 1.0)*	15.7 (15.6–15.8)	0.3 (0.2 to 0.4)*	16.0 (16.0–16.1)	−1.0 (−1.1 to −0.9)*	15.0 (15.0–15.1)	0.3 (0.3 to 0.4)*	15.4 (15.3–15.4)	1.3 (1.2 to 1.3)*	−0.6 (−0.7 to −0.6)*
America 8: White | Low-income Appalachia and Lower	15.5 (15.4–15.5)	0.4 (0.3 to 0.4)*	15.8 (15.8–15.9)	0.0 (−0.1 to 0.0)	15.8 (15.7–15.8)	−0.5 (−0.5 to −0.4)*	15.3 (15.3–15.4)	−0.4 (−0.4 to −0.3)*	14.9 (14.9–15.0)	0.3 (0.3* to 0.4)	−0.8 (−0.9 to −0.8)*

Mississippi Valley											
America 9: Black | Nonmetro and low-income South	14.7 (14.5–14.8)	0.6 (0.4 to 0.8)*	15.2 (15.1–15.4)	0.2 (0.1 to 0.4)*	15.5 (15.3–15.6)	−1.1 (−1.3 to −1.0)*	14.3 (14.2–14.4)	0.2 (0.0 to 0.4)*	14.5 (14.4–14.6)	0.8 (0.6 to 1.0)*	−1.0 (−1.1 to −0.8)*
America 10: AIAN | West	14.5 (13.9–15.1)	0.1 (−0.2 to 0.5)	14.7 (14.1–15.2)	0.3 (0.0 to 0.6)*	15.0 (14.4–15.5)	−1.3 (−1.5 to −1.0)*	13.7 (13.1–14.2)	−0.1 (−0.3 to 0.2)	13.6 (13.0–14.2)	0.4 (0.1 to 0.7)*	−1.4 (−1.6 to −1.1)*

Numbers in parentheses are 95% uncertainty intervals.
*Indicates that the uncertainty bounds do not encompass 0.

Figure S11: Remaining life expectancy, age 85 years, males

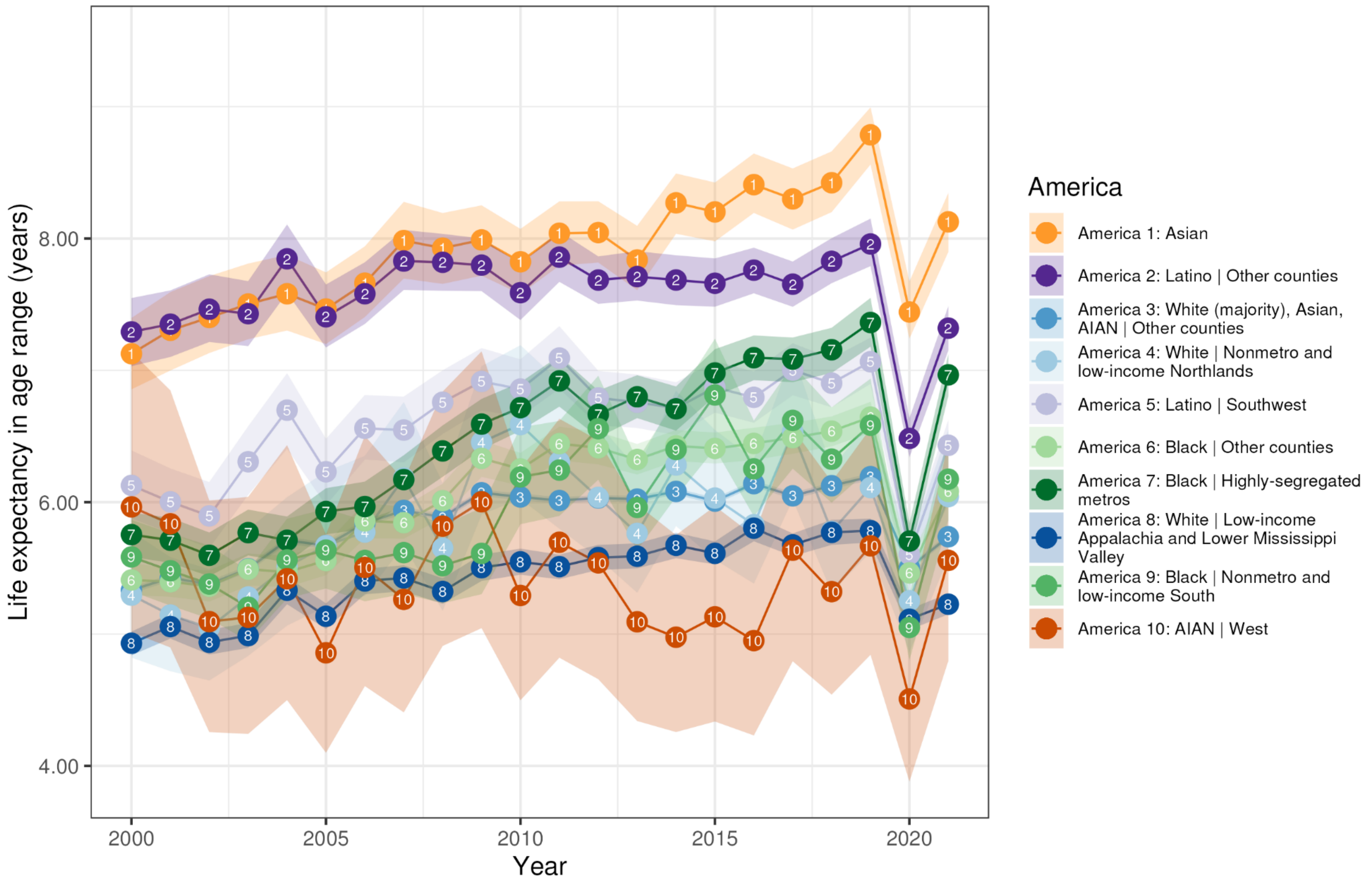

Table S11: Remaining life expectancy, age 85 years, males

America	Life expectancy, 2000	Change, 2000–10	Life expectancy, 2010	Change, 2010–19	Life expectancy, 2019	Change, 2019–20	Life expectancy, 2020	Change, 2020–21	Life expectancy, 2021	Change, 2000–19	Change, 2019–21
America 1: Asian	7.1 (6.9–7.4)	0.7 (0.4 to 1.0)*	7.8 (7.6–8.1)	1.0 (0.8 to 1.2)*	8.8 (8.6–9.0)	−1.3 (−1.5 to −1.2)*	7.4 (7.2–7.7)	0.7 (0.5 to 0.8)*	8.1 (7.9–8.3)	1.7 (1.4 to 1.9)*	−0.7 (−0.8 to −0.5)*
America 2: Latino | Other counties	7.3 (7.0–7.5)	0.3 (0.1 to 0.5)*	7.6 (7.4–7.8)	0.4 (0.2 to 0.5)*	8.0 (7.8–8.2)	−1.5 (−1.6 to −1.3)*	6.5 (6.3–6.6)	0.8 (0.7 to 1.0)*	7.3 (7.2–7.5)	0.7 (0.4 to 0.9)*	−0.6 (−0.8 to −0.5)*
America 3: White (majority), Asian, AIAN | Other counties	5.3 (5.3–5.4)	0.7 (0.7 to 0.7)*	6.0 (6.0–6.1)	0.2 (0.1 to 0.2)*	6.2 (6.2–6.2)	−0.7 (−0.7 to −0.7)*	5.5 (5.5–5.5)	0.2 (0.2 to 0.3)*	5.7 (5.7–5.8)	0.9 (0.8 to 0.9)*	−0.5 (−0.5 to −0.4)*
America 4: White | Nonmetro and low-income Northlands	5.3 (4.8–5.8)	1.3 (0.5 to 2.0)*	6.6 (6.0–7.2)	−0.5 (−1.3 to 0.3)	6.1 (5.6–6.6)	−0.9 (−1.5 to −0.2)*	5.3 (4.9–5.7)	0.8 (0.2 to 1.5)*	6.0 (5.6–6.5)	0.8 (0.1 to 1.5)*	−0.1 (−0.8 to 0.6)
America 5: Latino | Southwest	6.1 (5.9–6.4)	0.7 (0.4 to 1.0)*	6.9 (6.7–7.1)	0.2 (0.0 to 0.4)	7.1 (6.9–7.2)	−1.5 (−1.7 to −1.3)*	5.6 (5.5–5.7)	0.8 (0.7 to 1.0)*	6.4 (6.3–6.6)	0.9 (0.6 to 1.2)*	−0.6 (−0.8 to −0.4)*
America 6: Black | Other counties	5.4 (5.3–5.5)	0.8 (0.7 to 1.0)*	6.3 (6.1–6.4)	0.4 (0.2 to 0.5)*	6.6 (6.5–6.8)	−1.2 (−1.3 to −1.1)*	5.5 (5.4–5.6)	0.6 (0.5 to 0.7)*	6.1 (6.0–6.2)	1.2 (1.1 to 1.4)*	−0.6 (−0.7 to −0.4)*
America 7: Black | Highly-segregated metros	5.8 (5.6–5.9)	1.0 (0.7 to 1.2)*	6.7 (6.5–6.9)	0.6 (0.4 to 0.9)*	7.4 (7.2–7.5)	−1.7 (−1.9 to −1.4)*	5.7 (5.6–5.8)	1.3 (1.1 to 1.4)*	7.0 (6.8–7.1)	1.6 (1.4 to 1.8)*	−0.4 (−0.6 to −0.2)*
America 8: White | Low-income Appalachia and Lower	4.9 (4.8–5.0)	0.6 (0.5 to 0.7)*	5.5 (5.5–5.6)	0.2 (0.1 to 0.4)*	5.8 (5.7–5.9)	−0.7 (−0.8 to −0.5)*	5.1 (5.0–5.2)	0.1 (0.0 to 0.2)	5.2 (5.2–5.3)	0.9 (0.7 to 1.0)*	−0.6 (−0.7 to −0.4)*

Mississippi Valley											
America 9: Black | Nonmetro and low-income South	5.6 (5.3–5.9)	0.6 (0.1 to 1.1)*	6.2 (5.8–6.6)	0.4 (−0.1 to 0.9)	6.6 (6.3–6.9)	−1.5 (−2.0 to −1.1)*	5.1 (4.8–5.3)	1.1 (0.7 to 1.6)*	6.2 (5.9–6.5)	1.0 (0.5 to 1.4)*	−0.4 (−0.9 to 0.1)
America 10: AIAN | West	6.0 (5.0–7.1)	−0.7 (−1.5 to 0.2)	5.3 (4.5–6.3)	0.4 (−0.3 to 1.0)	5.7 (4.8–6.6)	−1.2 (−1.7 to −0.7)*	4.5 (3.9–5.2)	1.1 (0.6 to 1.6)*	5.6 (4.8–6.4)	−0.3 (−1.1 to 0.5)	−0.1 (−0.7 to 0.4)

Numbers in parentheses are 95% uncertainty intervals.
*Indicates that the uncertainty bounds do not encompass 0.

Figure S12: Remaining life expectancy, age 85 years, females

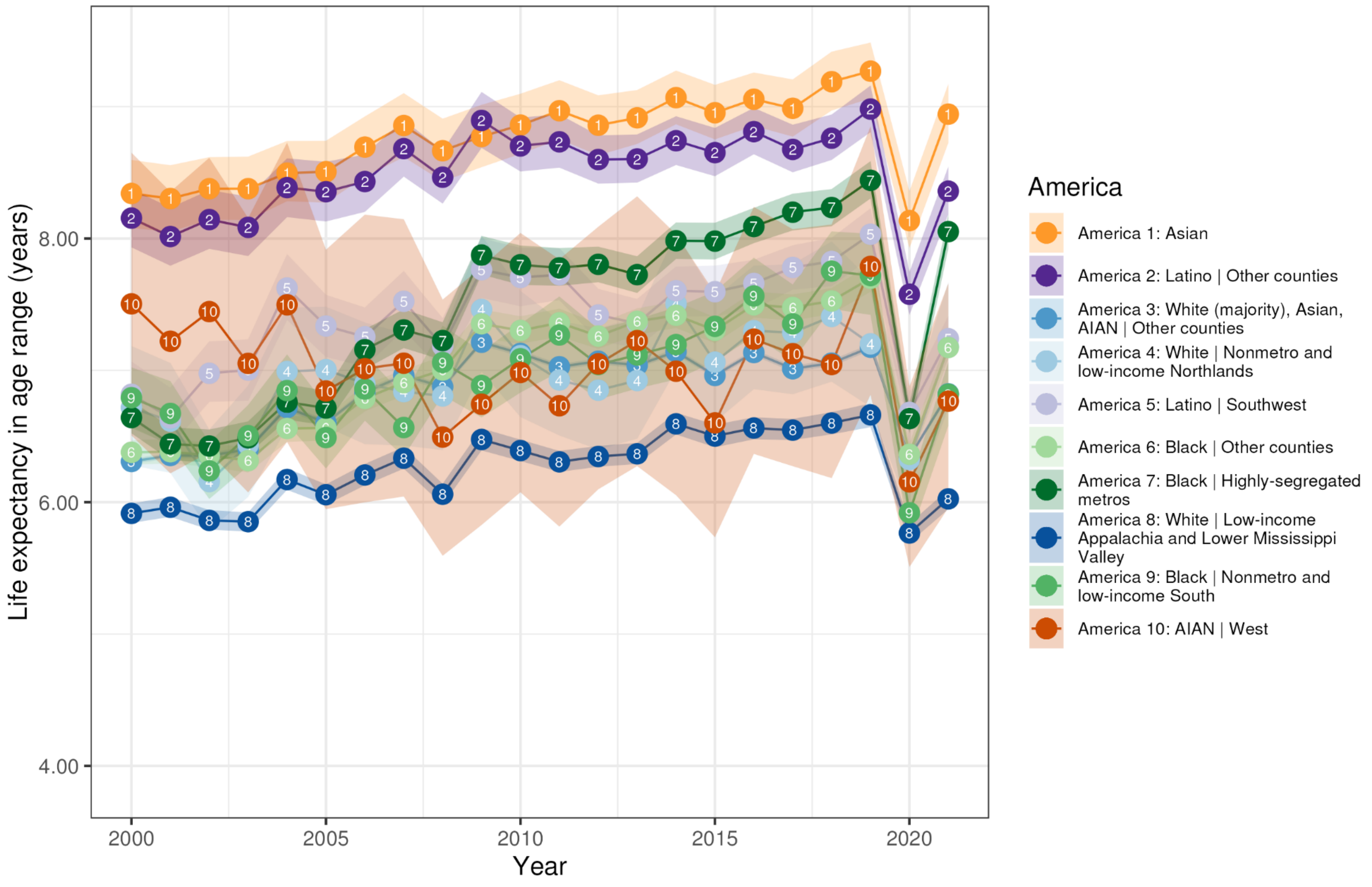

Table S12: Remaining life expectancy, age 85 years, females

America	Life expectancy, 2000	Change, 2000–10	Life expectancy, 2010	Change, 2010–19	Life expectancy, 2019	Change, 2019–20	Life expectancy, 2020	Change, 2020–21	Life expectancy, 2021	Change, 2000–19	Change, 2019–21
America 1: Asian	8.3 (8.1–8.6)	0.5 (0.3 to 0.8)*	8.9 (8.6–9.1)	0.4 (0.2 to 0.6)*	9.3 (9.1–9.5)	−1.1 (−1.3 to −1.0)*	8.1 (7.9–8.4)	0.8 (0.7 to 0.9)*	8.9 (8.7–9.2)	0.9 (0.7 to 1.1)*	−0.3 (−0.5 to −0.2)*
America 2: Latino | Other counties	8.2 (7.9–8.4)	0.5 (0.3 to 0.7)*	8.7 (8.5–8.9)	0.3 (0.1 to 0.4)*	9.0 (8.8–9.2)	−1.4 (−1.5 to −1.3)*	7.6 (7.4–7.7)	0.8 (0.7 to 0.9)*	8.4 (8.2–8.5)	0.8 (0.7 to 1.0)*	−0.6 (−0.7 to −0.5)*
America 3: White (majority), Asian, AIAN | Other counties	6.3 (6.3–6.3)	0.8 (0.8 to 0.8)*	7.1 (7.1–7.2)	0.0 (0.0 to 0.1)*	7.2 (7.2–7.2)	−0.8 (−0.9 to −0.8)*	6.3 (6.3–6.4)	0.5 (0.5 to 0.5)*	6.8 (6.8–6.8)	0.9 (0.8 to 0.9)*	−0.4 (−0.4 to −0.3)*
America 4: White | Nonmetro and low-income Northlands	6.7 (6.3–7.2)	0.4 (−0.3 to 1.0)	7.1 (6.6–7.6)	0.1 (−0.7 to 0.8)	7.2 (6.7–7.7)	−0.9 (−1.5 to −0.3)*	6.3 (5.9–6.7)	0.9 (0.2 to 1.5)*	7.2 (6.7–7.7)	0.5 (−0.1 to 1.1)	0.0 (−0.7 to 0.6)
America 5: Latino | Southwest	6.8 (6.6–7.1)	0.9 (0.6 to 1.2)*	7.7 (7.5–7.9)	0.3 (0.1 to 0.6)*	8.0 (7.9–8.2)	−1.4 (−1.5 to −1.2)*	6.7 (6.5–6.8)	0.6 (0.4 to 0.7)*	7.2 (7.1–7.4)	1.2 (1.0 to 1.4)*	−0.8 (−1.0 to −0.6)*
America 6: Black | Other counties	6.4 (6.3–6.5)	0.9 (0.8 to 1.1)*	7.3 (7.2–7.4)	0.4 (0.3 to 0.5)*	7.7 (7.6–7.8)	−1.3 (−1.4 to −1.2)*	6.4 (6.3–6.4)	0.8 (0.7 to 0.9)*	7.2 (7.1–7.3)	1.3 (1.2 to 1.4)*	−0.5 (−0.6 to −0.4)*
America 7: Black | Highly-segregated metros	6.6 (6.5–6.8)	1.2 (1.0 to 1.3)*	7.8 (7.6–7.9)	0.6 (0.5 to 0.8)*	8.4 (8.3–8.6)	−1.8 (−2.0 to −1.6)*	6.6 (6.5–6.7)	1.4 (1.3 to 1.6)*	8.1 (7.9–8.2)	1.8 (1.6 to 2.0)*	−0.4 (−0.6 to −0.2)*
America 8: White | Low-income Appalachia and Lower	5.9 (5.8–6.0)	0.5 (0.4 to 0.6)*	6.4 (6.3–6.5)	0.3 (0.2 to 0.4)*	6.7 (6.6–6.7)	−0.9 (−1.0 to −0.8)*	5.8 (5.7–5.8)	0.3 (0.2 to 0.4)*	6.0 (5.9–6.1)	0.7 (0.6 to 0.9)*	−0.6 (−0.8 to −0.5)*

Mississippi Valley											
America 9: Black | Nonmetro and low-income South	6.8 (6.6–7.0)	0.3 (0.0 to 0.6)	7.1 (6.8–7.4)	0.6 (0.2 to 1.0)*	7.7 (7.4–8.0)	–1.8 (–2.1 to –1.5)*	5.9 (5.7–6.1)	0.9 (0.6 to 1.2)*	6.8 (6.6–7.1)	0.9 (0.5 to 1.3)*	–0.9 (–1.3 to –0.5)*
America 10: AIAN | West	7.5 (6.5–8.7)	–0.5 (–1.3 to 0.3)	7.0 (6.1–8.0)	0.8 (0.1 to 1.5)*	7.8 (6.8–8.8)	–1.6 (–2.3 to –1.0)*	6.2 (5.5–6.9)	0.6 (0.1 to 1.1)*	6.8 (6.0–7.7)	0.3 (–0.5 to 1.0)	–1.0 (–1.7 to –0.4)*

Numbers in parentheses are 95% uncertainty intervals.
*Indicates that the uncertainty bounds do not encompass 0.
